# Combined transcriptome and metabolome analysis of *Polygonatum cyrtonema* Hua in response to *Botrytis deweyae* infection

**DOI:** 10.3389/fpls.2025.1617308

**Published:** 2025-07-29

**Authors:** Fuqiang Yin, Wanli Ma, Zhien Xiao, Yuxin Liu, Tiantian Guo, Yue Yuan, Shaotian Zhang, Guoli Li, Ming Liu

**Affiliations:** ^1^ College of Biological and Food Engineering, Chongqing Three Gorges University, Chongqing, China; ^2^ Guangdong Provincial Key Laboratory of Plant Molecular Breeding, South China Agricultural University, Guangzhou, China; ^3^ Department of Basic Medical Sciences, Chongqing Three Gorges Medical College, Chongqing, China

**Keywords:** *Botrytis deweyae*, metabolome, transcriptome, disease resistance, *Polygonatum cyrtonema* Hua

## Abstract

The fungal pathogen *Botrytis deweyae*, first identified as the causative agent of gray mold disease in China, has become a critical biotic constraint limiting the sustainable production of *Polygonatum cyrtonema* Hua in major cultivation regions. To investigate the physiological reactions and transcriptome gene changes of *P. cyrtonema* after *B. deweyae* infection, in this study, we investigated the defense enzyme activity, transcriptome differential genes (DEGs), and differential metabolites (DAMs) of *P. cyrtonema*. When *B. deweyae* invaded the leaves of *P. cyrtonema*, the activities of phenylalanine deaminase (PAL), catalase (CAT), and peroxidase (POD) increased. The most responsive Kyoto Encyclopedia of Genes and Genomes (KEGG) pathways in the transcriptome were plant-pathogen interaction, plant hormone signal transduction, the mitogen-activated protein kinase (MAPK) signaling pathway, and phenylpropanoid biosynthesis (phenylpropanoid biosynthesis) metabolic pathways. Among the DEGs, AP2 ERF-ERFs, WRKYs, and C2H2 were highly predictive of transcription factors (TFs), with WRKYs being important TFs in the *P. cyrtonema* MAPK pathway. In the metabolome, coumaric acid, α-linolenic acid, and jasmonic acid (JA) are important metabolites that respond to *B. deweyae* infection. Correlation analysis between the transcriptome and metabolome revealed that phenylpropanoid metabolism and α-linolenic acid metabolism pathways are associated with the most significant response of *P. cyrtonema* to *B. deweyae* infection, with phenylpyruvate being an important metabolite in the phenylpropanoid metabolic pathway. Additionally, the observed upregulation of α-linolenic acid and JA synthesis suggests potential activation of JA-dependent induced systemic resistance (ISR) against *B. deweyae*, possibly mediated through downstream MYC transcription factors. These findings indicate that JA signaling contributes significantly to *P. cyrtonema* defense response against fungal infection. Our findings provide foundational insights that may support the development of disease-resistant cultivars or biostimulant strategies for *P. cyrtonema* and related medicinal plants.

## 1 Introduction

*Polygonatum cyrtonema* Hua is a perennial herbaceous plant belonging to the family Asparagaceae (Qin et al., 2024). It is also an important economic crop that combines food and medicinal uses (Wang et al., 2019). In recent years, with the continuous expansion of planting areas, gray mold disease causing by *Botrytis.* spp has become quite severe in Wanzhou District, Chongqing. *B. cinerea* (the canonical gray mold pathogen) has been extensively characterized regarding its pathogenicity mechanisms on many hosts (Chen et al., 2021). Our recent research has revealed that *B. deweya* causes a significantly higher disease incidence—ranging from 30% to 45%—compared to its congeners (Ma et al., 2023). The field of this species has been severely impacted due to the scarcity of varieties that are resistant to *Botrytis*. Moreover, the wild type of *P. cyrtonema* has been classified as an endangered species (Suyal, 2024). Given these challenges, it is of utmost importance to identify and develop resources that are resistant to *Botrytis* in order to address the production issues and ensure the sustainable cultivation of *P. cyrtonema*.

When pathogen infection occurs, pathogen-related molecular patterns (PAMPs) and DAMPs can activate the plant PTI immune response (Couto and Zipfel, 2016). Within a short period, plants can undergo rapid defense responses, including the activation of the mitogen-activated protein kinase (MAPK) cascade, an increase in reactive oxygen species (ROS) levels, and the initiation of the salicylic acid (SA) and jasmonic acid (JA) signaling pathways (Yuan et al., 2021). ROS accumulation activates protein kinase-mediated programmed cell death (PCD) (Petrov et al., 2015). To avoid excessive ROS buildup, plants use a mechanism to maintain appropriate cellular ROS levels, thereby protecting normal tissues. Cysteine catalase (CAT), peroxidase (POD), and superoxide dismutase (SOD) are important protective enzymes for eliminating ROS in plants and play crucial roles in response to *B. cinerea* infection (Meng et al., 2022).

The main mechanism of the immune response against *B. cinerea* involves the activation of PTI by DAMPs (Lai and Mengiste, 2013), which is the product of pathogenic microorganisms degrading host cell components. The PTI pathway induced by DAMPs requires the participation of the genes MPK3 and MPK6 in the MAPK signaling pathway (Galletti et al., 2011). MPK3/MPK6 enhances the stability of these two transcription factors (TFs) by directly phosphorylating the ERF6 and WRKY33 TFs, thus increasing resistance to gray mold disease (Meng et al., 2013). The MAPK pathway of tobacco also increases resistance to gray mold disease through the WRKY transcription factor (Adachi et al., 2016). WRKY 33 can target genes involved in JA/ethylene (ET) signaling and phytohormone biosynthesis (Wang et al., 2020). Sorbic acid (SA), JA, and ET are considered to be the three most important regulators of the plant disease resistance signal transduction process. The SA signaling pathway can be used by plants to increase resistance to gray mold disease (Mishra et al., 2024). JA is involved in the basic resistance of gray mold fungus, which can induce the synthesis of plant alkaloids and phenolic acids. These substances can combine with pathogenic bacterial proteins to produce toxic effects, thus inhibiting their spread (Wang et al., 2020). Various plants contain antibacterial substances in their bodies (Mangalagiri et al., 2021); when pathogenic bacteria are perceived, the phenylpropionamide metabolic pathway quickly starts synthesizing phenols, mushrooms, and flavonoids (Gao et al., 2024; Ye et al., 2016). By disrupting the cell membrane structure of *B. cinerea* and reducing total lipid content, the artemether phenol in potatoes inhibits mycelial growth (Zhang et al., 2019). The expression of betaine in tobacco leaves significantly improved resistance to gray mold (Polturak et al., 2017). The accumulation of coumaric acid and malic acid in ginseng leaves inhibits the growth of *B. cinerea* (Liu et al., 2020).

During the long-term coevolution of plants and pathogens, due to the complex infection strategies of pathogenic bacteria, plants have evolved different strategies to cope with various pathogen invasions. Different plants exhibit different methods of resisting pathogen infections. Transcriptomics technologies help visualize gene expression differences through (Gene Ontology) (GO) enrichment and Kyoto Encyclopedia of Genes and Genomes (KEGG) enrichment, allowing the study of the molecular mechanisms underlying the response of host plants to pathogens (Zhang et al., 2013). Metabolomics is a method used to qualitatively and quantitatively analyze metabolites produced in the body through analytical methods such as mass spectrometry and chromatography (Meng et al., 2024). The correlation analysis of metabolomics and transcriptomics not only reveals changes in plant metabolites but also facilitates a deeper investigation into the causes of these changes, i.e., variations in gene transcription levels, thus helping elucidate the relevant mechanisms of interaction.

Previously, we first reported gray mold disease caused by *B. deweyae* in *P. cyrtonema* (Ma et al., 2023). To investigate the physiological responses and transcriptomic gene changes of *P. cyrtonema* under *B. deweyae* infection. In this study, through an integrated approach combining defense enzyme activity profiling, transcriptomic analysis, and metabolomic characterization, we systematically investigated the molecular response mechanisms of *P. cyrtonema* to *B. deweyae*. Our findings demonstrate that the MAPK signaling cascade activates pivotal transcription factors and defense enzyme system (POD, PAL, and CAT) orchestrate early defense responses, while α-linolenic acid dependent JA biosynthesis pathway coordinates phytohormone-mediated resistance through metabolic reprogramming. These pathogen-responsive mechanisms are further modulated by F-box E3 ubiquitin ligases via dynamic protein regulation. Our systematic deciphering of this defense network provides crucial insights for molecular-guided breeding of disease-resistant *P. cyrtonema* cultivars.

## 2 Results and discussion

### 2.1 Symptoms of gray mold in *Polygonatum cyrtonema*

**Figure 1:**
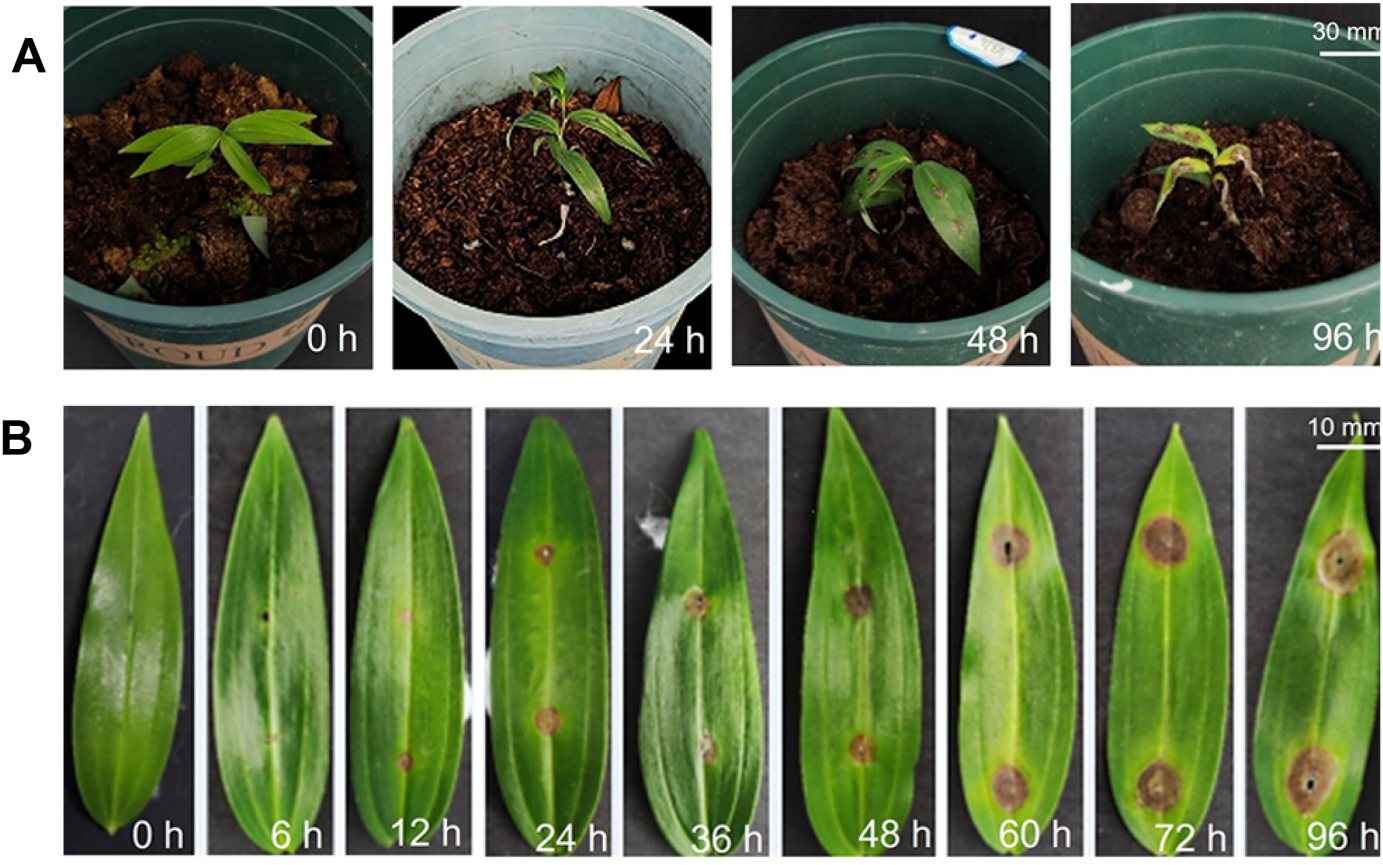
Diseases symptoms of gray mold in *Polygonatum cyrtonema* at different time points. **(A)** Symptoms of potted plants (for transcriptome sequencing); **(B)** Symptoms of single leaf disease (for enzyme activity assay).

The leaves were inoculated at 0 h to maintain a healthy and disease-free state, and disease spots started appearing 24 h after inoculation (**Figure 1A**). The diseased parts were watery and mottled. As the inoculation time increased, the disease spots expanded into ellipsoids and grew white aerial hyphae. At 96 h, the leaves withered, and the whole plant was close to death (**Figure 1B**).

### 2.2 Defense enzyme activity is enhanced after inoculation with *Botrytis deweyae*

**Figure 2:**
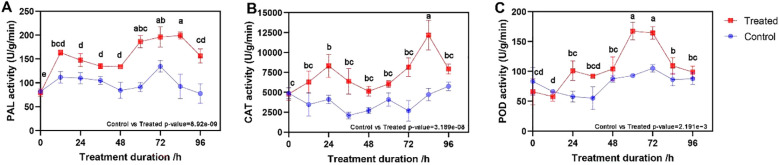
Changes in defense enzyme activity of *Polygonatum cyrtonema* leaves after inoculation with *Botrytis deweyae*. **(A)** phenylalanine deaminase (PAL) activity; **(B)** catalase (CAT) activity, and **(C)** peroxidase (POD) activity. Different lowercase letters indicate statistically significant differences among post-inoculation treatments (p < 0.05) based on Tukey’s honestly significant difference (HSD) test following ANOVA.

When *B. deweyae* invaded the leaves of *P. cyrtonema*, the activities of all three defense enzymes were greater than those of the control leaves (t-test, all *p-values <*0.01), with the activity of the PAL enzyme rapidly increasing from 0 to 12 h and then slowly decreasing before increasing to the maximum value of 199.02 U/g (units per g protein) at 84 h (**Figure 2A**). The CAT activity increased from 0 to 24 h and then decreased to the minimum value of 5134.56 U/g at 48 h, followed by a rapid increase to the maximum value of 12177.37 U/g at 84 h (**Figure 2B**). The POD enzyme activity started increasing at 12 h and generally tended to increase, reaching 167.407 U/g at 60 h, after which it started to decrease (**Figure 2C**). Compared to the symptoms of *P. cyrtonema* leaves inoculated with *B. deweyae*, *P. cyrtonema* leaves between 72 h and 84 h may have already lost their resistance capability, and they gradually withered and died at 84 h of infection.

### 2.3 Comparative analysis of transcriptome sample data

A total of 75.60 Gb of clean data were obtained from 12 samples, the amount of clean data from each sample reached 5.77 Gb, the Q30 percentage was 89.81% or greater, and 80,417 unigenes were obtained after assembly. Among them, 22,756 unigenes had a length of more than 1 kb (**Supplementary Table S1**), and 40,844 unigenes were annotated by functional annotation.

For each sample, boxplots of gene expression levels were plotted to examine the dispersion of gene expression levels across individual samples, compare the overall gene expression levels of different samples (**Supplementary Figure S1A**), assess the dispersion of samples, and note that the principal component analysis (PCA) distances within the group were relatively close. In contrast, the main components of Tpoly1 and Tpoly4 were alike for the treatment groups, whereas Tpoly1, Tpoly2, and Tpoly3 showed variation (**Supplementary Figure S1B**).

**Figure 3:**
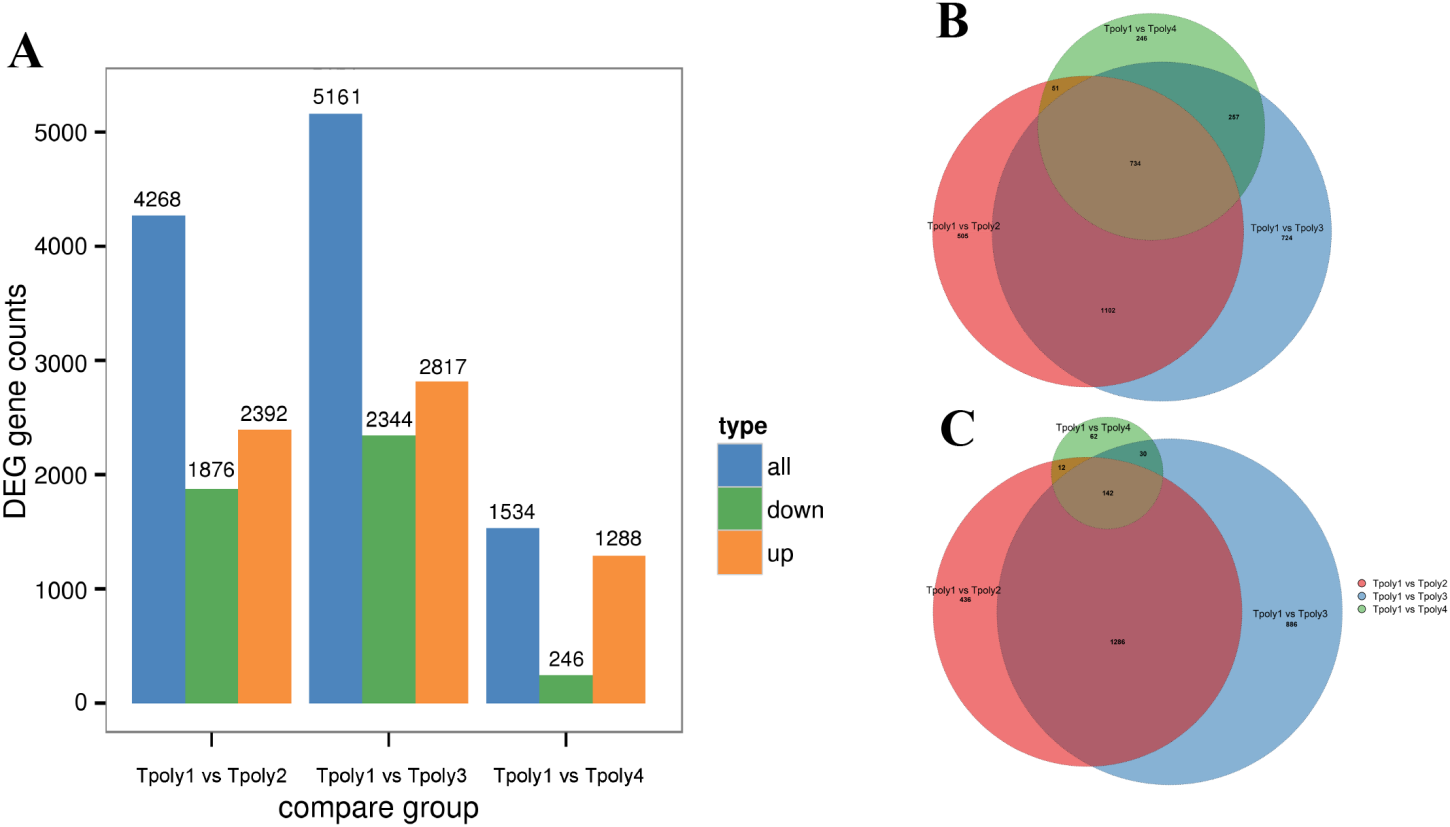
Statistics of the number of differentially expressed genes. **(A)** Histogram of the number of differentially expressed genes; **(B)** Euler plot of differentially up-regulated gene set; **(C)** Euler plot of differentially down-regulated gene set.

In the three groups, 10,963 genes were differentially expressed in the gene set; 6,497 genes were upregulated, and 4,466 genes were downregulated. The number of differentially expressed genes (DEGs) in group G2 was the highest among the three comparison groups, with 4268 genes, while the lowest number of DEGs in G3 was only 1534 (**Figure 3A**).

To determine the relationships among the three DEGs, a Venn diagram analysis was performed on the DEGs in the three gene sets of G1, G2, and G3, and Euler plots were drawn using the BIC network platform (**Figures 3B, C**). The results revealed 734 co-expressed upregulated DEGs and 142 co-expressed downregulated genes.

#### 2.3.1 Pathways related to plant immune responses are annotated by KEGG

**Figure 4:**
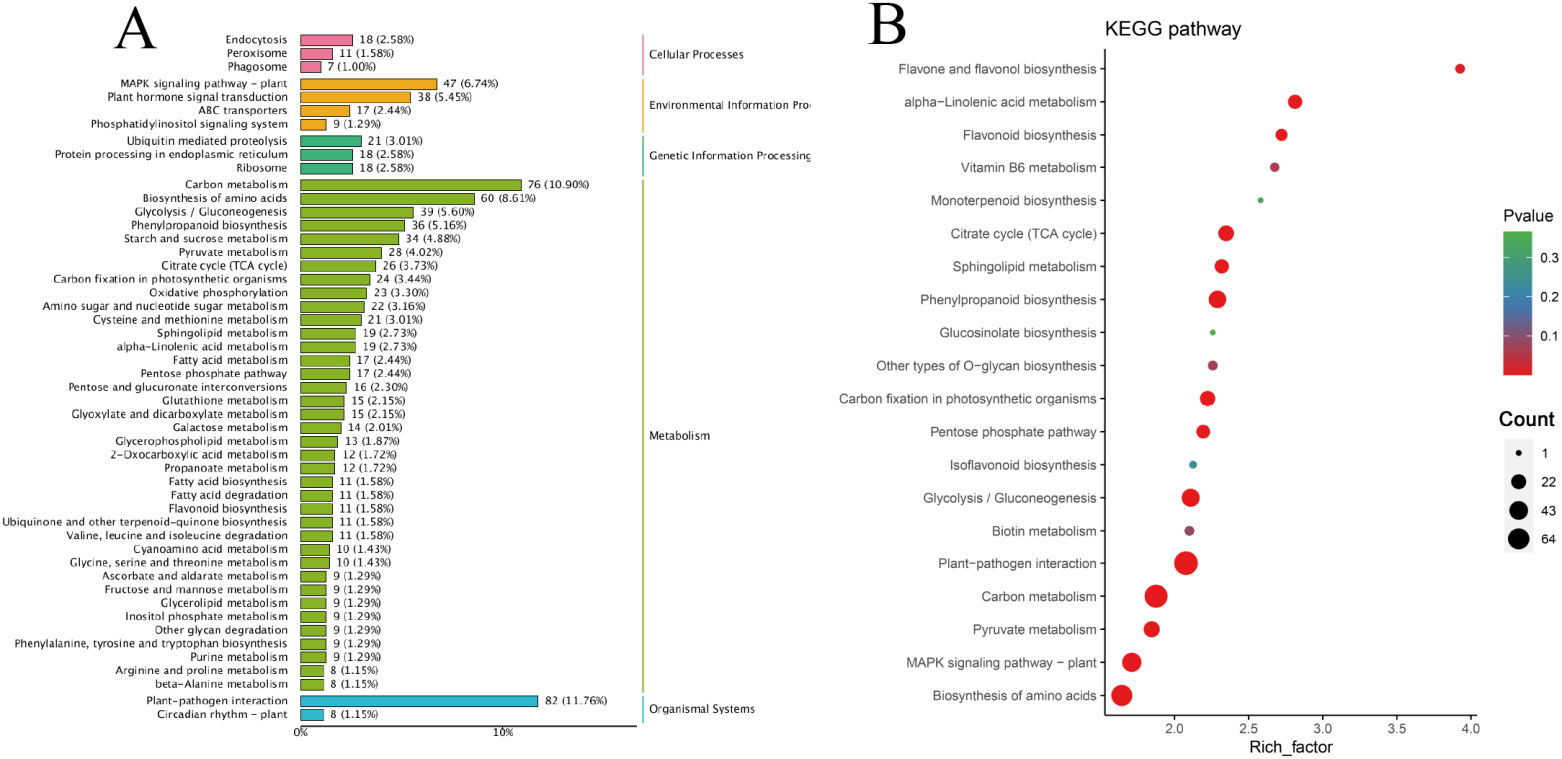
Classification and enrichment of KEGG. **(A)** KEGG pathway classification line diagram. The ordinate represents the KEGG pathway annotated by KEGG Ortholog database, and the horizontal axis represents the number of genes annotated; **(B)** KEGG enrichment bubble diagram. The ordinate represents the KEGG pathway. The abscissa represents the Rich factor. The larger the Rich factor, the greater the enrichment. The larger the point, the greater the number of differential genes enriched in the pathway. The redder the color of the dots, the more significant the enrichment.

Among the 1102 + 734 upregulated DEGs in the G1 and G2 gene sets, the KEGG classification map revealed that 11 genes associated with cellular processes were annotated to the peroxisome pathway, and the MAPK signaling pathway was the most enriched in the environmental information processing signaling pathway and plant hormone signal transduction. In total, 21 genes involved in ubiquitin-mediated proteolysis were annotated to genetic information processing, and 36 genes were involved in phenylpropanoid biosynthesis. Moreover, 82 genes were associated with the plant-pathogen interaction pathway (**Figure 4A**). Carbon metabolism and plant-pathogen interactions were the most enriched pathways in the KEGG analysis. Phenylpropanoid biosynthesis, α-linolenic acid metabolism, flavonoid biosynthesis, and sphingolipid metabolism pathways were also enriched (**Figure 4B**).

#### 2.3.2 Oxidoreductase activity pathway was enrichment

In the GO classification diagram, DEGs were the most abundant metabolic process, and 120 genes related to response to stimulus, 37 genes related to signaling and detoxification, and four genes related to immune system processes were annotated. In terms of cell composition (CC), a single cell enriched the most gene entries. The binding and catalytic activities were annotated to the molecular function (MF) with the largest number of DEG entries, and 15 genes involved in antioxidant activity were annotated (**Supplementary Figure S2**).

**Figure 5:**
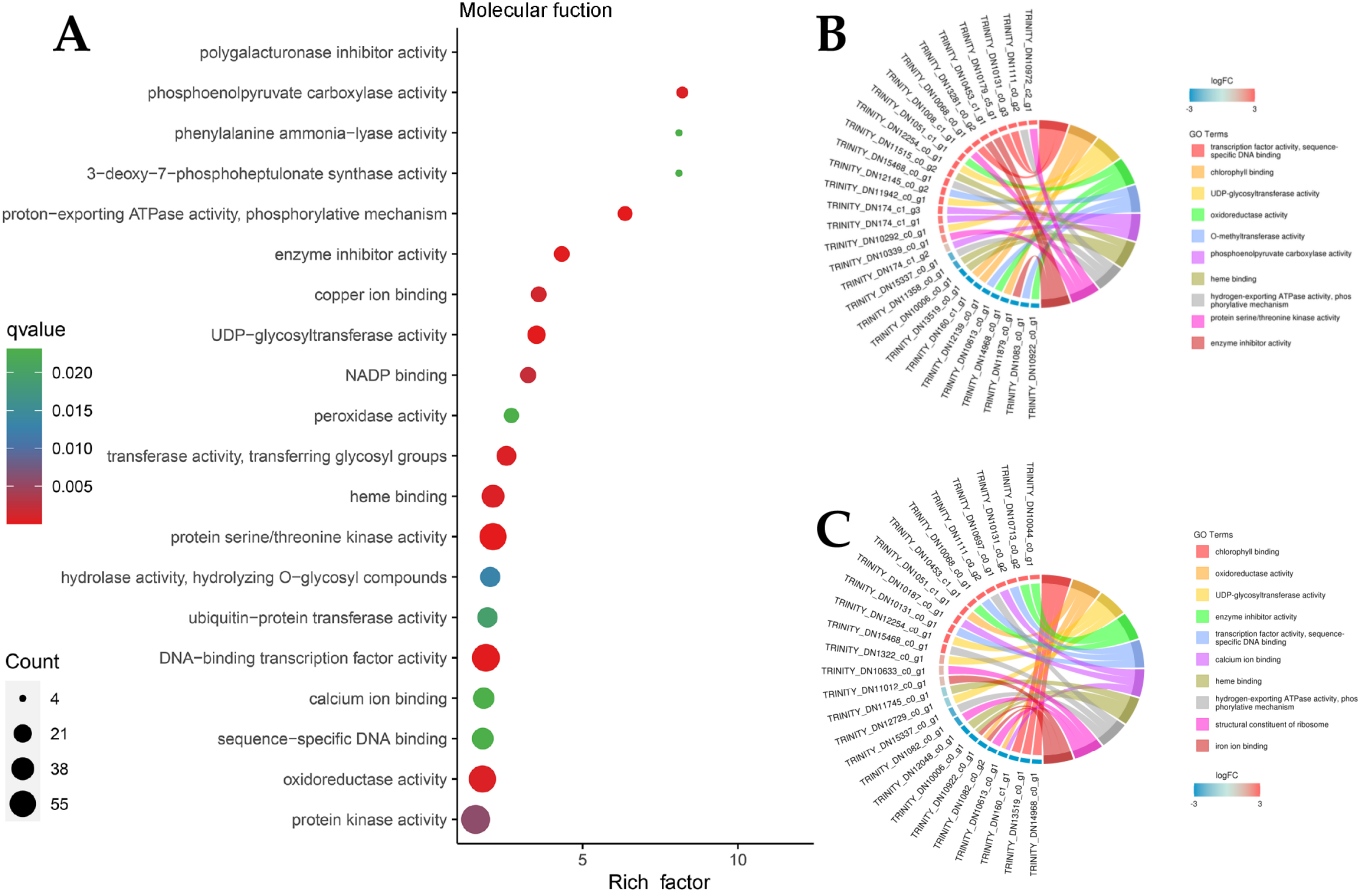
DEGs classification enrichment of Molecular function (MF). **(A)** MF enrichment Bubble diagram. The ordinate represents the KEGG pathway. The abscissa represents the rich factor. The larger the rich factor, the greater the enrichment. The larger the point, the greater the number of differential genes enriched in the pathway. The redder the color of the dots, the more significant the enrichment; **(B, C)** MF enrichment Network diagram. The left half represents different genes and their relative expression levels, the darker the color, the more obvious the up-regulation of the modified genes, and the right half represents the annotation to different MF pathways, which are represented by different color patches and connecting lines.

**Figure 6:**
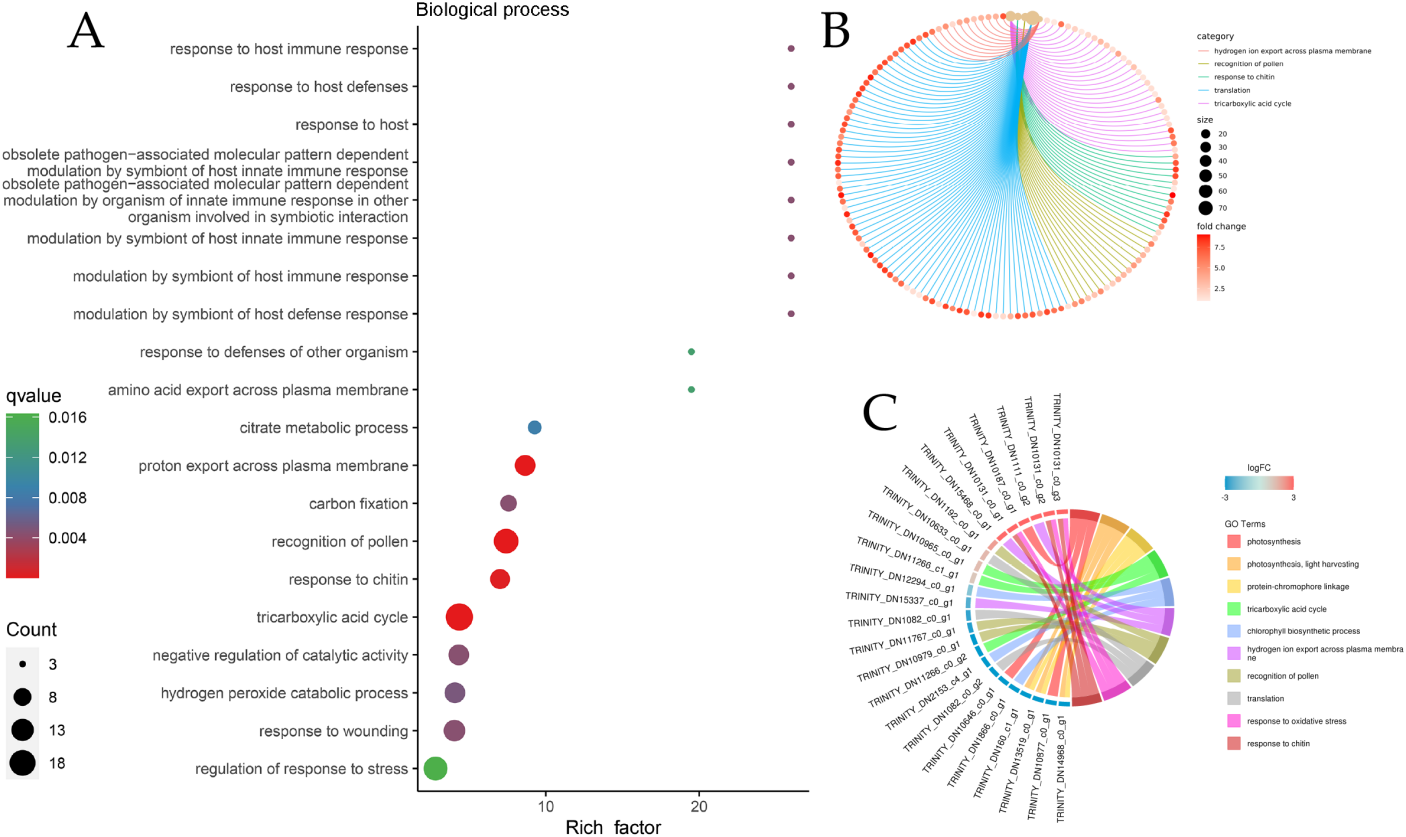
DEGs classification enrichment of biological processes (BP). **(A)** BP enrichment Bubble diagram; **(B)** BP enrichment chord diagram. The line color represents the different pathways to which the annotation is reached. The size of the dot represents the number of genes, the larger the size, the greater the enrichment; **(C)** BP enrichment Network diagram.

Owing to the large number of plant disease resistance genes involved in BP and MF, GO enrichment analysis and GO enrichment hierarchy analysis were conducted. The GO enrichment bubble diagram revealed that the tricarboxylic acid cycle had the largest number of DEG entries annotated in BP, and 10 genes involved in response to chitin were significantly enriched, which were significantly enriched with the host immune response; additionally, defense response genes were annotated (**Figure 5A**). The GO enrichment chord diagram revealed the gene expression of chitin in response to pathogens, in which DN10131 was significantly upregulated (**Figures 5B, C**), triggered the immune response of plants, and participated in the process of response to oxidative stress, which indicated that it plays an important role in plant immune signaling. In the MF bubble map, 69 genes were annotated to protein kinase activity, and the highest enrichment was protein serine/threonine kinase activity, which may act as PRRs on plant cell membranes. In total, 13 peroxidase activity genes and four phenylalanine ammonia-lyase activity genes were also enriched in small amounts (**Figure 6A**). DN1051 (GO:0003824), DN10922, and DN10613 were involved in the redox process, and DN10131 and DN10068 were upregulated in the G1 and G2 groups (**Figures 6B, C**).

#### 2.3.3 The key transcription factor WRKY regulates fungal infections

Transcription factors (TFs) were predicted for the 734 co-expressed upregulated genes using a Venn diagram (**Supplementary Figure S3A**), and heatmaps were generated for the top three types of TFs with the most gene entries (**Supplementary Figure S3B**), among which the first three gene families were AP2ERF-ERF, WRKY, and C2H2. Numerous studies have identified the important roles of TFs in plant defense. One of the most studied MYB TFs is Botrytis susceptible 1 (BOS1), which restricts necrosis triggered by *B. cinera* (Mengiste et al., 2003). In addition, WRKY33 is phosphorylated by MPK3/MPK6 to regulate the biosynthesis of phytoalexin in response to pathogen infection (Ren et al., 2008). Arabidopsis CCCH protein C3H14 contributes to basal defense against *B. cinerea* mainly through the WRKY33-dependent pathway (Wang et al., 2020). WRKY transcription factors act as key regulators in plant disease resistance signaling pathways (e.g. strawberry, tomato) (Shu et al., 2021), responding to biotic stress by regulating genes involved in innate immunity, hormone signaling pathways, and phytoalexin synthesis (Lee et al., 2023).

#### 2.3.4 Three co-expression patterns were identified to be functionally linked to susceptibility development

**Figure 7:**
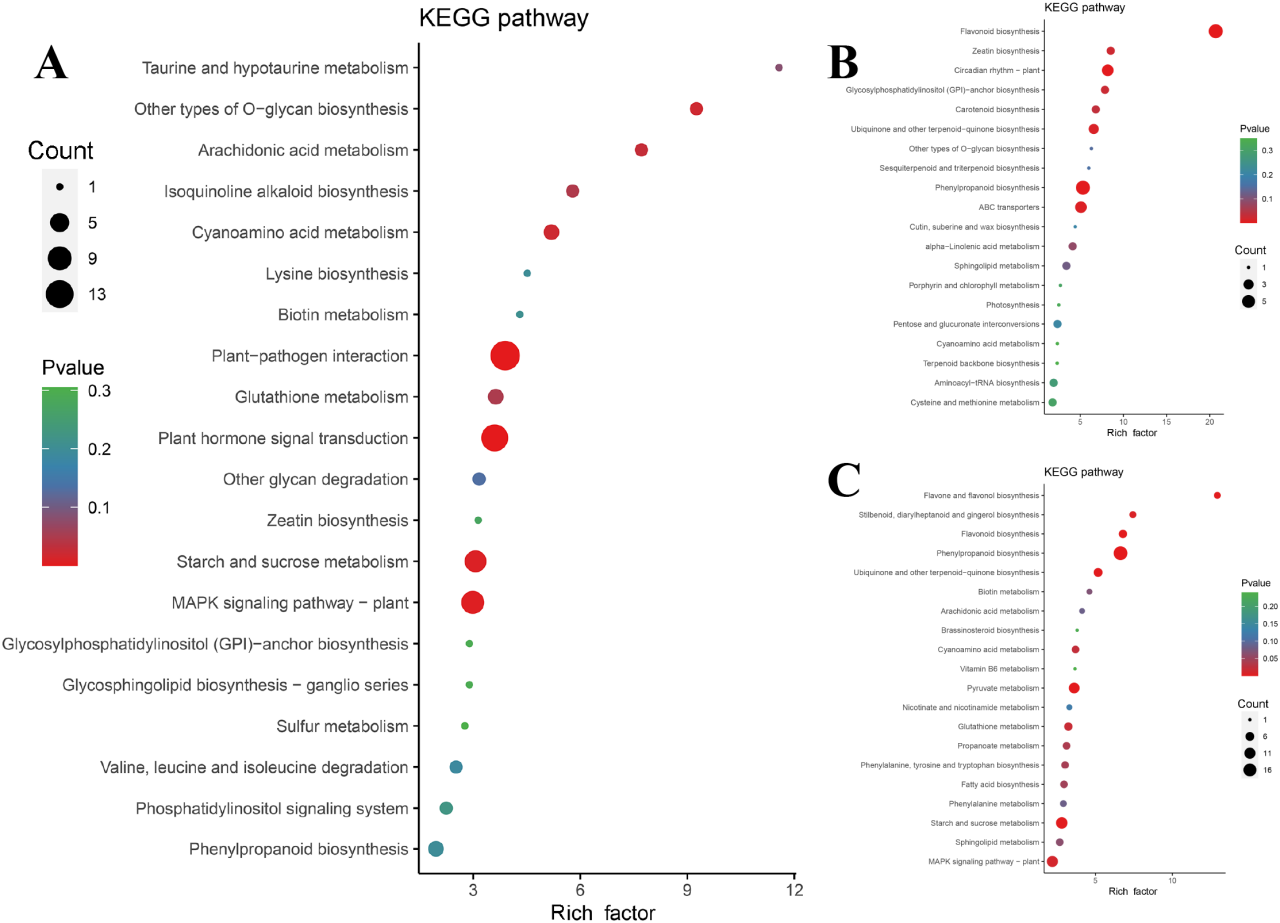
Enrichment diagram of co-expressed trend gene KEGG. **(A)** 189 gene KEGG enrichment Bubble Diagram; **(B)** 133 gene KEGG enrichment Bubble Diagram; **(C)** Bubble Diagram of KEGG enrichment of 317 gene. The ordinate represents the KEGG pathway. The abscissa represents the rich factor. The larger the rich factor, the greater the enrichment. The larger the point, the greater the number of differential genes enriched in the pathway. The redder the color of the dots, the more significant the enrichment.

To assess genes with the same expression pattern, a co-expression trend analysis of three sets of DEGs was conducted. The expression dynamics can be clustered into 11 expression patterns (**Supplementary Figure S4**). According to the co-expression trend diagram, three patterns with consistent upregulated expression trends were selected for subsequent analysis (**Supplementary Figure S4** A, H, and K). Among the 189 genes whose expression was consistent, those related to plant-pathogen interactions, plant hormone signal transduction, and the MAPK signaling pathway were significantly enriched (**Figure 7A**), which was the result of interactions between *P. cyrtonema* and *B. deweyae*.

Metabolites or metabolite intermediates associated with antibacterial activity, such as phenylpropanoid biosynthesis, flavonoid biosynthesis, ubiquinone, and other terpenoid-quinone biosynthesis, and α-linolenic acid metabolism, were enriched in the other two modes. Additionally, sphingolipid metabolism was also enriched in the other two modes (**Figures 7B, C**).

#### 2.3.5 MAPK signaling pathway mediates primary defense responses

When attacked by *B. deweyae*, the receptor protein kinase BAK1/FLS senses pathogenic effector factors and triggers the MAPK signaling immune response (**Figure 1**), including ROS mechanisms and the activation of transcription processes to resist pathogens. This indicated that brassinosteroid-related receptor kinase 1 (BAK1) and LRR receptor-like serine/threonine protein kinase (FLS) play key roles in activating MAPK signaling as two LRRs, while WRKY TFs activate downstream genes for *B. deweyae* resistance, with WRKY 33 being the most annotated transcription factor, which activates the camalexin synthesis process downstream. WRKY 22 TFs mediate cell death by activating the transcription of the senescence receptor kinase FRK1 (**Supplementary Figure S5**). The MAPK signaling pathway is an essential component of plant immunity, mediating hypersensitive response (HR) and associated cell death (Adachi et al., 2016). MPK3/MPK6 phosphorylates WRKY transcription factors including WRKY22, WRKY23, WRKY29, WRKY46 and WRKY53, which mediate the pathogen induced plant defense response (Xie et al., 2021). In our previous analysis of TFs, we propose that this conserved MAPK-WRKY regulatory module likely functions in *P. cyrtonema* to coordinate defense against fungal pathogens.

#### 2.3.6 Protein-protein interaction network associated with defense-enriched pathways

**Figure 8:**
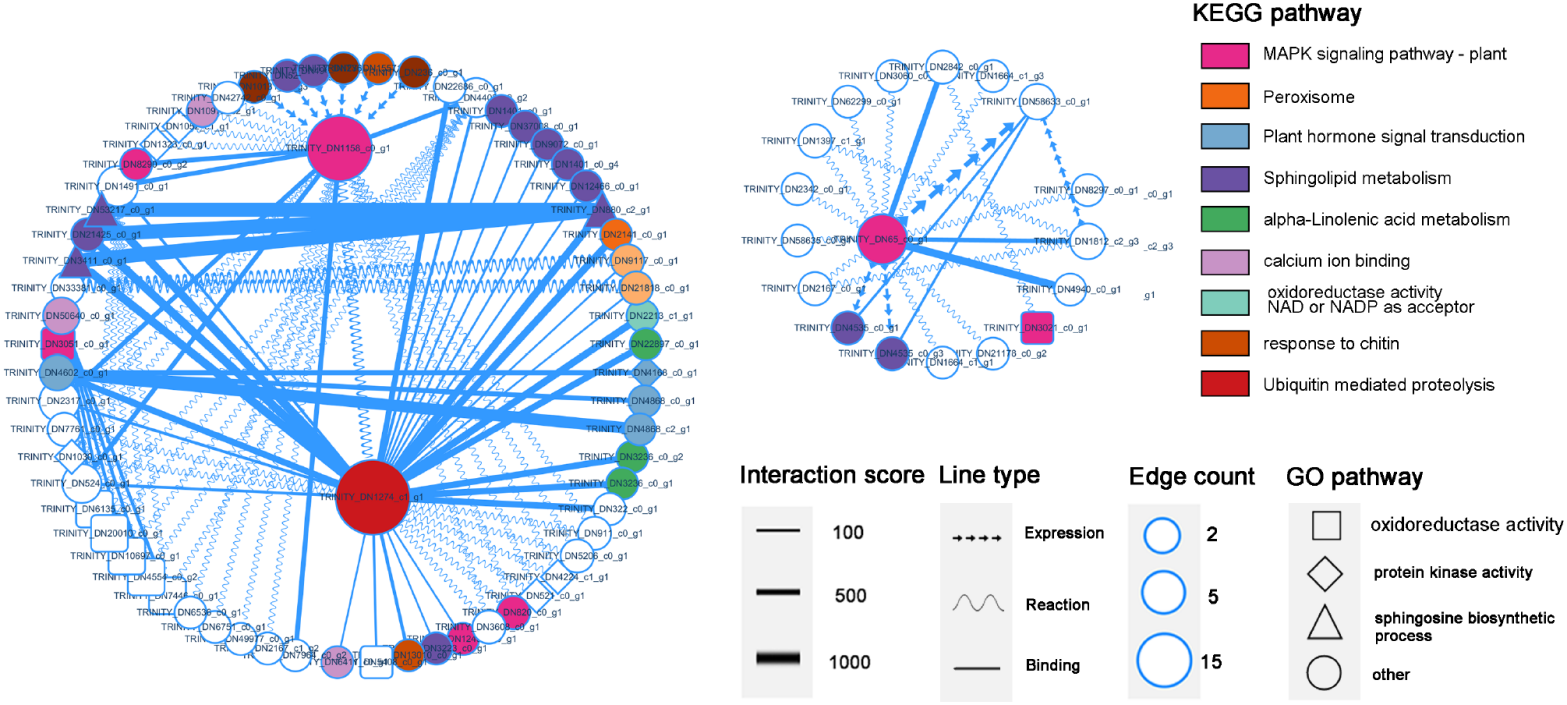
PPI protein interaction diagram of DEGs. The size of each node represents the number of Edge count, the width of the line represents the degree of interaction score, and is automatically scored by the database. The shape of the line represents the interaction relationship, and the node shape reflects the function of genes in the KEGG and GO pathways.

Based on the KEGG and GO classification information, the protein interaction network diagram nodes were edited, and the functions and interactions of each gene were displayed, among which the ubiquitin-mediated protein hydrolysis gene DN1274_c1_g1 (ko04120) showed binding relationships with multiple disease resistance genes, including the MAPK signaling pathway, α-linolenic acid metabolism pathway, and plant hormone signaling pathway. This indicated that protein ubiquitination is crucial for activating and regulating gene expression in the immune pathways of *P. cyrtonema*. Four genes (DN3411_c0_g1/DN21425_c0_g1/DN53217_c0_g1/DN880_c2_g1) (ko00600) involved in sphingolipid metabolism most strongly interacted with DN1274_c1_g1, and these four genes were found to be closely related. The two genes involved in MAPK signaling, including DN1158_c0_g1 (ko04626) and DN65_ c0_g1 (ko04016), also presented a high degree of correlation in the network diagram, and multiple pathways, such as chitin and sphingoesteric acid metabolism, promoted MAPK signaling (**Figure 8**).

#### 2.3.7 Verify differentially expressed genes in key pathways by qPCR

**Figure 9:**
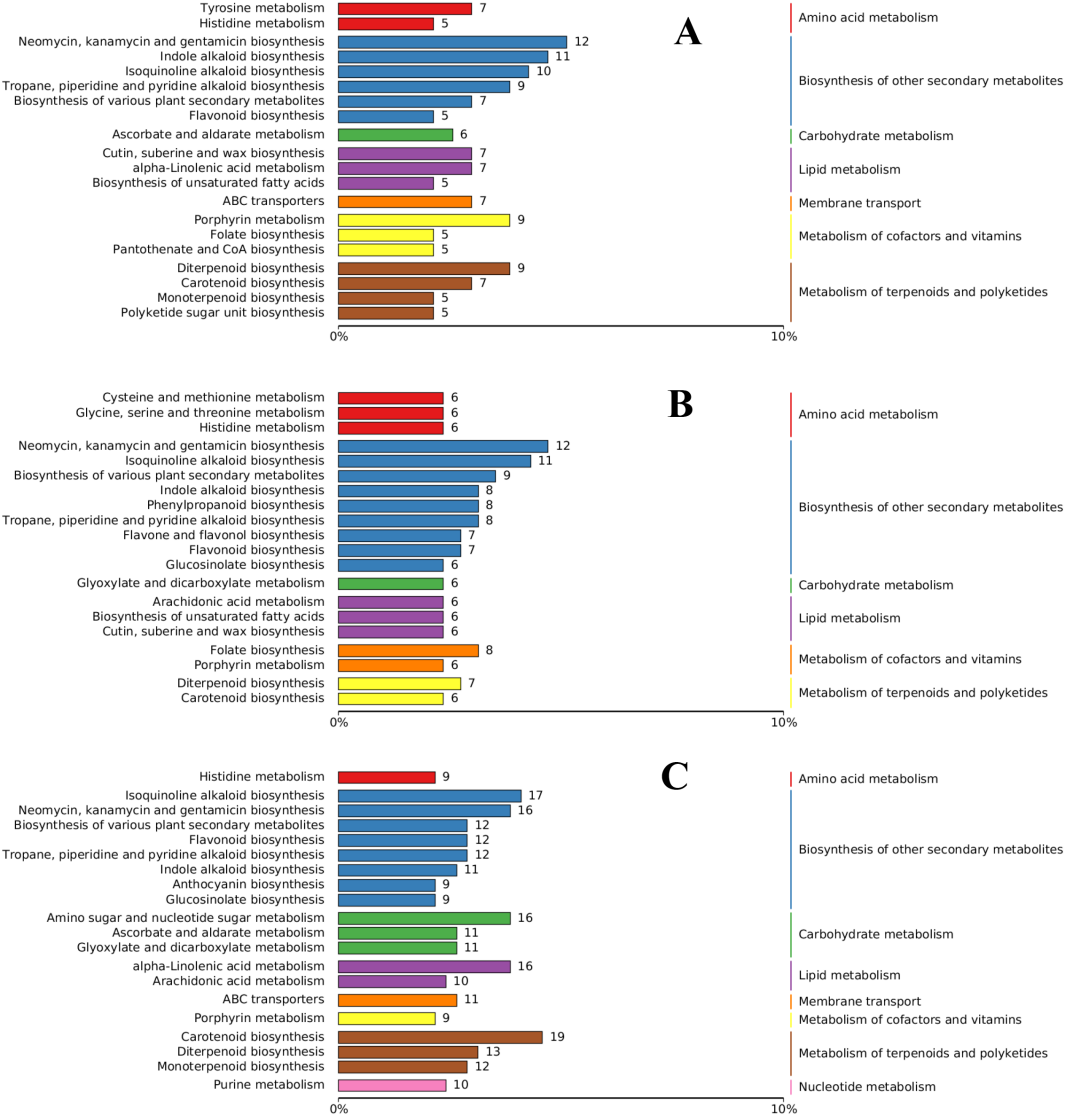
Functional annotation of differential metabolite KEGG in comparative groups. **(A)** M1 comparison group; **(B)** M2 comparison group; **(C)** M3 comparison group. The left ordinate represents the metabolic reaction, the right ordinate axis repre-sents the metabolic pathway, and the same color indicates the same metabolic pathway. The abscissa axis value represents the proportion of each metabolite.

Real-time PCR verification of the screened DEGs was conducted, and the correlation between the RNA-seq data and relative quantitative data was evaluated. The results of the qPCR analysis of the 12 DEGs are shown in **Figure 9**; the genes confirmed included the receptor protein kinase gene in MAPK signaling pathway DN65_c0_g1 (ko04016), the DN14157_c0_g1 (ko00940) gene of phenylpropanoid biosynthesis or catalytic activity (GO:0003824), the DN1401_c0_g1 (ko00600) gene of sphingomate metabolism, and the phenylpropanoid biosynthesis DN13372_c0_g1 (ko00940) or antioxidant activity (GO:0016209).

### 2.4 Metabolomic analysis in response to *Botrytis deweyae* infection

#### 2.4.1 Sample difference grouping and test results

We used the LC-QTOF platform for qualitative and quantitative metabolomics analysis of 16 samples and detected 24,245 peaks under default mode, of which 4,975 metabolites were annotated. By conducting PCA of correlations between samples to evaluate biological reproducibility within the group (**Supplementary Figure S6A**), the Spearman rank correlation coefficient (r) was used as an indicator of biological reproducibility (**Supplementary Figure S6B**). Based on the results of the PCA variable analysis and correlation assessment, the differential metabolite (DAMs) data obtained from this metabolomics study were found to be reliable and can be used for downstream DAMs screening. Based on the results of OPLS-DA (**Supplementary Figure S7**). Permutation testing confirmed the robustness of the OPLS-DA model. All permuted *R^2^Y* and *Q^2^Y* values (blue dots) fell below the original model’s metrics (red star), indicating the model’s validity (p < 0.001). The intercepts of the regression lines (*Q^2^* < 0.05) further support non-overfitting (**Supplementary Figure S8**). The models built in the three comparison groups were all valid and could be used for subsequent analysis.

#### 2.4.2 KEGG enrichment analysis of differential metabolites

**Figure 10:**
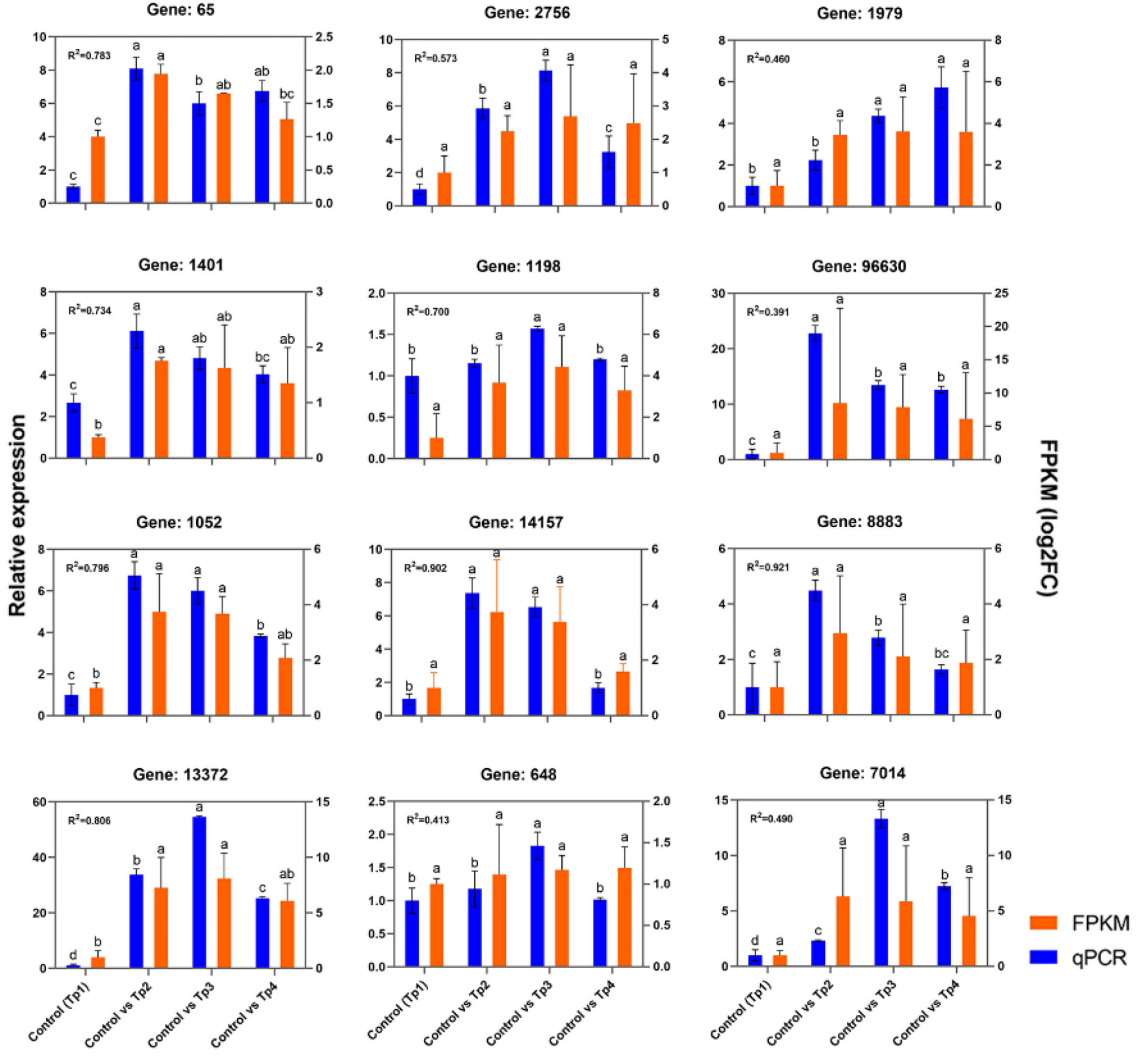
qRT-PCR validation of differentially expressed genes in the transcriptome of *Polygonatum cyrtonema* post infection. The Spearman’s rho values quantify the rank-based correlation between RNA-seq and qRT-PCR measurements at each time point (*P* value < 0.05). Different letters indicate statistically significant differences of RNA-seq and qRT-PCR among the three comparison groups at p < 0.05 according to Tukey’s test.

The DAMs in each differential group were annotated using the KEGG database, and the substances with antifungal activity in the three groups were annotated to the biosynthesis of other secondary metabolites. Most of the annotated metabolites were antibiotic metabolites, such as neomycin, kanamycin, and gentamicin. Flavonoid biosynthesis, indole alkaloid, quinoline alkaloid, and alkaloid-related metabolites were also annotated to the three groups. Arachidonic acid metabolism, α-linolenic acid metabolism, and cutin, suberin, and wax biosynthesis were the most annotated DAMs in lipid metabolism. Other DAMs, such as diterpenoid biosynthesis and carotenoid biosynthesis, were annotated to the metabolism of terpenoids and polyketides (**Figure 10**). Eight DAMs were annotated to the phenylpropanoid biosynthesis pathway in the M2. Although KEGG analysis revealed apparent enrichment of neomycin, kanamycin, and gentamicin, these compounds are not synthesized in plants. This observation likely reflects conserved primary metabolic components shared between plants and bacteria, such as enzymes and intermediates involved in amino sugar and nucleotide sugar metabolism (e.g., UDP-glucose). To improve pathway resolution and avoid such artifacts, propose future studies will employ complementary tools like MapMan or Reactome for functional enrichment analysis, which may better distinguish plant-specific metabolic processes.

The abundance score (DA score) reflects the overall changes in all metabolites in the metabolic pathway, and a positive score indicates that the expression of all annotated metabolites in the pathway is upregulated, and vice versa. The DA scores for the three comparison groups are shown in **Supplementary Figure S9**. The five pathways with the greatest enrichment in each difference group were assessed by association analysis and combined with the results of enrichment network diagram analysis. In the M1 comparison group, the sulfur- relay system had the highest score, and the biosynthesis of alkaloids, plant hormone signaling, and the biosynthesis of cutin, serine, and wax were also upregulated. The enrichment network diagram showed that indole alkaloids and other biosynthesized alkaloids were the most annotated differentially expressed metabolites (with 11 metabolites in total) (**Supplementary Figure S9A**). In the M2 comparison group, β-lactam resistance had the highest score, and the metabolites associated with phenylpropanoid biosynthesis increased in score (**Supplementary Figure S9B**). In the M3 comparison group, the highest score was for α-linolenic acid metabolism, accounting for 11 metabolites, followed by microterpene biosynthesis (**Supplementary Figure S9C**). Furthermore, the bubble plot of KEGG enrichment factor for DAMs confirmed significant enrichment of cutin, serine, and wax biosynthesis (**Supplementary Figure S10A, B**) and alpha-linolenic acid metabolism (**Supplementary Figure S10C**) in the metabolome of *P cyrtonema*. The results of the KEGG enrichment analysis of the transcriptome revealed that α-linolenic acid metabolism was the most significant metabolite involved in disease resistance.

### 2.5 Integrative transcriptome-metabolome association analysis

#### 2.5.1 Correlation assessment of differential genes and metabolites

Subsequently, PCA was performed separately for the three differential groups, and the degree of association between the DAMs and the DEGs was evaluated by reducing the dimensionality of the DAMs to a few independent variables. The results of the analysis are shown in **Supplementary Figure S12**. By comparing the pathways associated with genes in the transcriptome and the pathways associated with metabolites in the metabolome, the number of common pathways involved was obtained, as shown in **Supplementary Figure S12**, with 79, 78, and 83 common metabolic pathways in the three differential groups, respectively.

#### 2.5.2 Annotation of differential genes and metabolites

The DEGs and metabolites in the three comparison groups were uniformly annotated on the KEGG pathway, and the top 30 pathways with significantly enriched differential genes/metabolites were plotted as a bar chart. Bubble plots were made using the KEGG pathways coenriched by transcriptomics and metabolomics. In the MG1 grouping bar chart, metabolic pathways related to keratin, serine, and wax biosynthesis were significantly different. As shown in the KEGG pathway bubble plot on the right (**Supplementary Figure S13A**), sphingolipid metabolism, flavonoid biosynthesis, phenylpropanoid biosynthesis, α-linolenic acid metabolism, and plant hormone signaling were significantly enriched. These pathways were also enriched in the MG2 group (**Supplementary Figure S13B**). In the MG3 group, genes and metabolites related to α-linolenic acid metabolism presented the greatest significance, whereas the DEGs and metabolites related to plant hormone signaling and keratin, serine, and wax biosynthesis presented increased significance (**Supplementary Figure S13C**).

#### 2.5.3 Jasmonic acid signaling and zeatin networks

**Figure 11:**
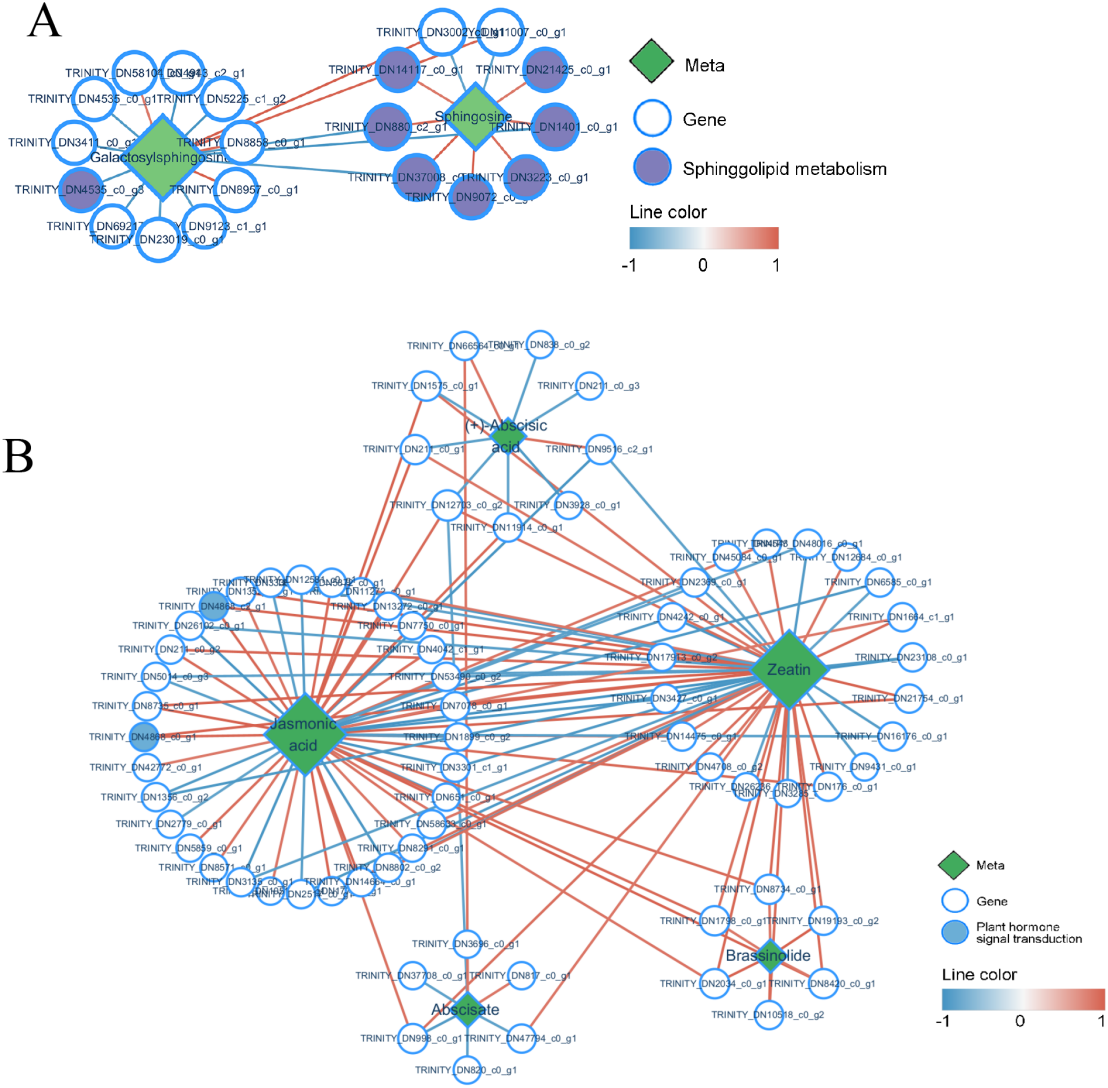
Network chart of differentially expressed genes and metabolites, **(A)** Network chart of sphingolipid metabolism; **(B)** Network chart of jasmonic acid and zeatin metabolism. The red line represents a positive correlation (Correlation coefficient Pearson, CCP <0.05), and the blue line represents a negative correlation.

In this study, the networks involved in Jasmonic acid (JA) synthesis and sphingolipid metabolism were annotated. Sphingolipids are a class of structurally complex lipid molecules containing long-chain sphingosine groups, with galactosyl sphingosine and sphingosine being key intermediate products in the sphingolipid metabolism pathway of *P. cyrtonema* (**Figure 11A**). Both metabolite levels increased after *B. deweyae* infected *P. cyrtonema*, and their gene expression also increased, which aligned with the transcriptome network diagram. Another annotated pathway was the synthesis of the plant hormone JA; we found (**Figure 11B**) that JA synthesis is influenced by abscisic acid (ABA), brassinolide, abscisate, and zeatin and is closely related to genes associated with zeaxanthin and JA. JA is an endogenous hormone that plays a very important role in plant disease resistance, and its gene expression is regulated by WRKY TFs, which can trigger HR-mediated disease resistance in plants (Shu et al., 2021). Zeatin is a novel plant hormone that can regulate various physiological mechanisms, such as growth, development, phototropism, and stress tolerance. Although abiotic stresses such as drought in wheat (*Triticum aestivum*) and cadmium toxicity in *Desmodesmus armatus* have been extensively studied (Piotrowska-Niczyporuk et al., 2024; Wang et al., 2023), research on their responses to biotic stress—particularly pathogen infection—remains relatively underexplored. In this experimental, the metabolic processes of zeatin are not significant, the network diagram showed that most genes involved in its synthesis are the same as those involved in JA synthesis, indicating that a synergistic relationship may exist between JA and zeatin, which can coregulate *P. cyrtonema* in the *B. deweyae* antidisease process, a hypothesis requiring functional validation through future interrogation of their crosstalk mechanisms.

#### 2.5.4 Defense-related phenylpropanoids and derivatives against *Botrytis deweyae* infection

**Figure 12:**
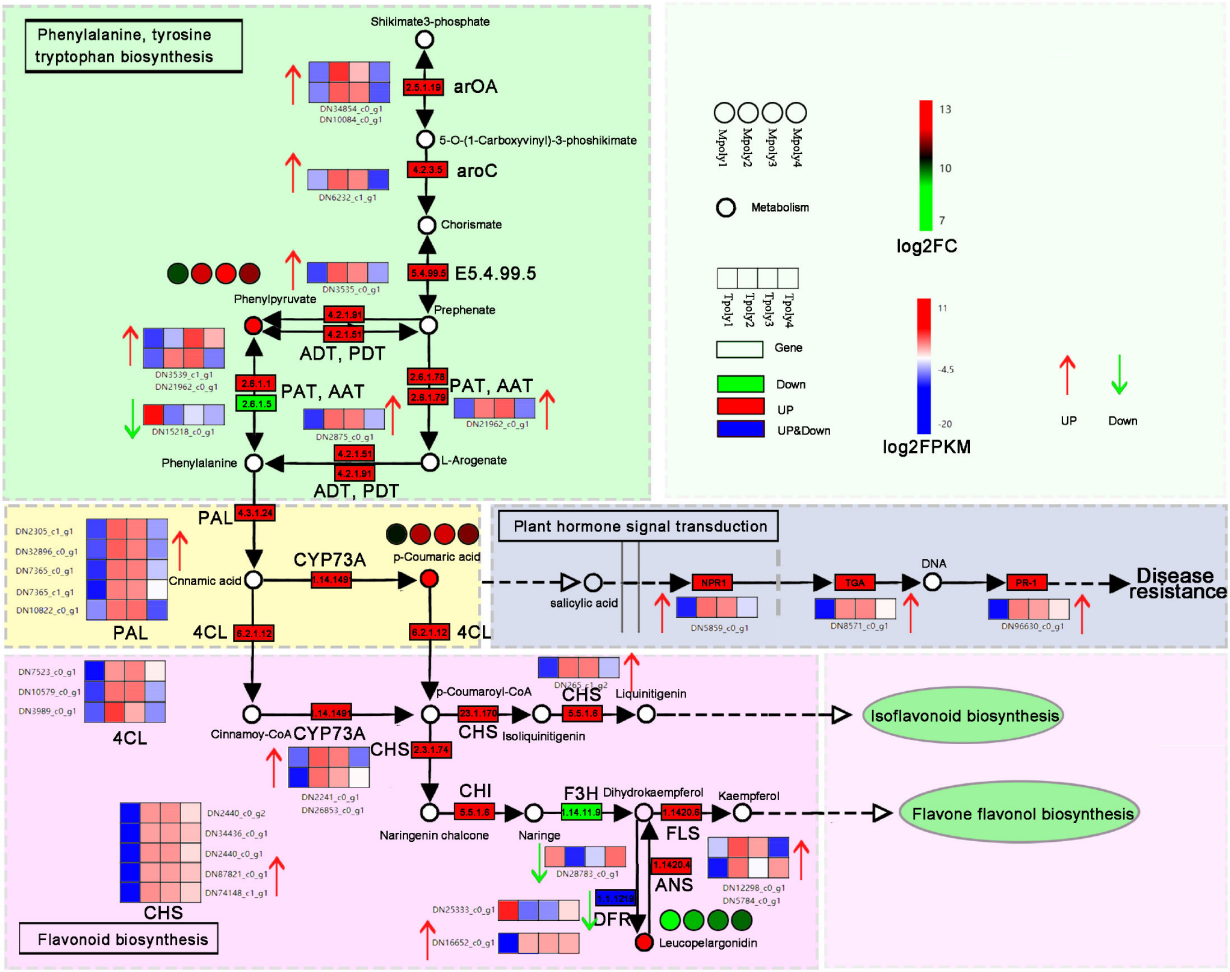
The synthesis of phenylalanine metabolites involved in the disease resistance pathway. Each gene is represented in the wireframe, the red shading represents the up-regulation of the gene, the green represents the down-regulation, and the heat map represents the enrichment level of the gene at four time points, and the redder the color indicates the more enrichment of the gene at that time point.

According to the phenylpropion synthesis pathway diagram (**Figure 12**), during *B. deweyae* infection, the activities of three metabolites, phenylpyruvate, p-coumaric acid, and leucopelargonidin, significantly increase in *P. cyrtonema*. Phenylpyruvate is the precursor for phenylalanine (Pen) synthesis, which is primarily synthesized into Pen by transferases such as aspartate aminotransferase (AST) and glutamate dehydrogenase (GDH). The phenylpropanoid biosynthesis pathway may indirectly influence the salicylate-mediated disease resistance signaling pathway. Studies have shown that p-coumaric acid has a significant inhibitory effect on the walnut anthracnose pathogen *Colletotrichum gloeosporioides*. In this study, Pen activated the downstream flavonoid biosynthesis pathway, where the chalcone synthase (CHS) gene plays a crucial role. In another pathway involved in flavonoid synthesis, leucopelargonidin promotes the formation of dihydrokaemplerol under the action of anthocyanin synthase (ANS), with rutin participating as a precursor in the downstream flavonoid synthesis pathway. Pen acts as a key substance in the phenylpropanoid metabolic pathway (Li et al., 2017). The phenylpropanoid metabolism-based defense responses towards pathogen attacks have been widely characterized in plants (Cui et al., 2024; Xiao et al., 2022). It can directly or indirectly activate plants to produce various secondary metabolites with antibacterial effects, such as flavonoids and phenols, *B. cinerea* quercetin dioxy genase *(BcQdo)* (catalyzing flavonoids degradation gene) involves in *B. cinerea* virulence towards the *Panax ginseng*, *△BcQdo* mutants showed increased flavonoids accumulation and reduced disease development (Chen et al., 2022).

#### 2.5.5 The α-Linolenic acid-to-jasmonate signaling cascade

**Figure 13:**
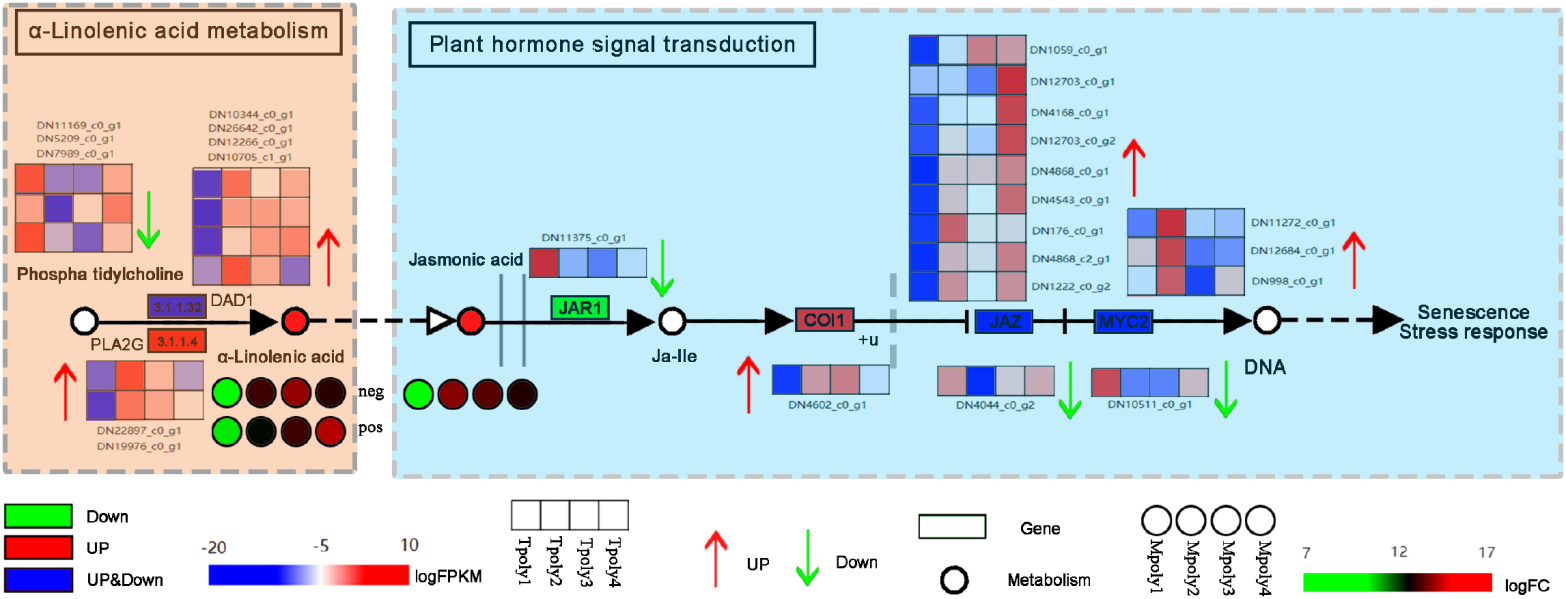
The antifungal signaling pathway of α-linolenic acid. Each gene is represented in the wireframe, the red shading represents the up-regulation of the gene, the green represents the down-regulation, and the heat map represents the enrichment level of the gene at four time points, and the redder the color indicates the more enrichment of the gene at that time point.

Linolenic acid (LnA) and linoleic acid (LA) are the main unsaturated fatty acids in plants (Lim et al., 2017). They are natural immune inducers in plants and can act as signaling molecules to induce the natural, systematic, and lasting resistance of plants to diseases (Dedyukhina et al., 2014). In this study, the metabolite α-linolenic acid was detected in the metabolome, and its activity significantly increased with increasing inoculation time (**Figure 13**), indirectly correlated with the plant hormone JA signaling pathway, with the metabolic levels of both showing a positive correlation. In the hormone signaling pathway, the JAZ (jasmonate ZIM-domain protein) protein complex, which is composed of the negative regulator JAZ and inositol pentophosphate molecules, has COI1 as a core member of the JAZ receptor complex (Wang, 2015). JA receptor COI 1 can specifically bind to JAZ, causing JAZ to be ubiquitinated and degraded by the proteasome, relieving the inhibition of JAZ in the transcriptional regulation of the JA pathway and thus participating in the transmission of downstream disease resistance genes (Srivastava et al., 2018). In this study, an increase in the expression of multiple JAZ genes regulated transcription and played a dominant role in the JA signal-mediated disease resistance process. Moreover, MYC2 acts as the core transcription factor of the JA signaling pathway, not only exerting positive regulatory effects on defense-related JA responses but also regulating the expression of the plant defense gene PDF1.2 (plant defensin 1.2) (Li et al., 2021), the genes COI1 and MYC2 were confirmed to participate in the JA signaling pathway, with MYC2 showing a significant association with *B. squamosa* resistance in onion (*Allium cepa*) (Lee et al., 2022). Additionally, allene oxide synthase (AOS) enzymes mediate the biosynthesis of JA from α-linolenic acid. The AOS-overexpressing line exhibited enhanced JA accumulation, which was associated with increased resistance to *B. squamosa* (Kim et al., 2025), suggesting that α-linolenic acid metabolism plays a crucial role in plant defense mechanisms. However, the regulatory role of α-linolenic acid in JA biosynthesis is currently inferred primarily from omics data, and its precise molecular mechanisms require further experimental dissection.

## 3 Conclusion and future research

**Figure 14:**
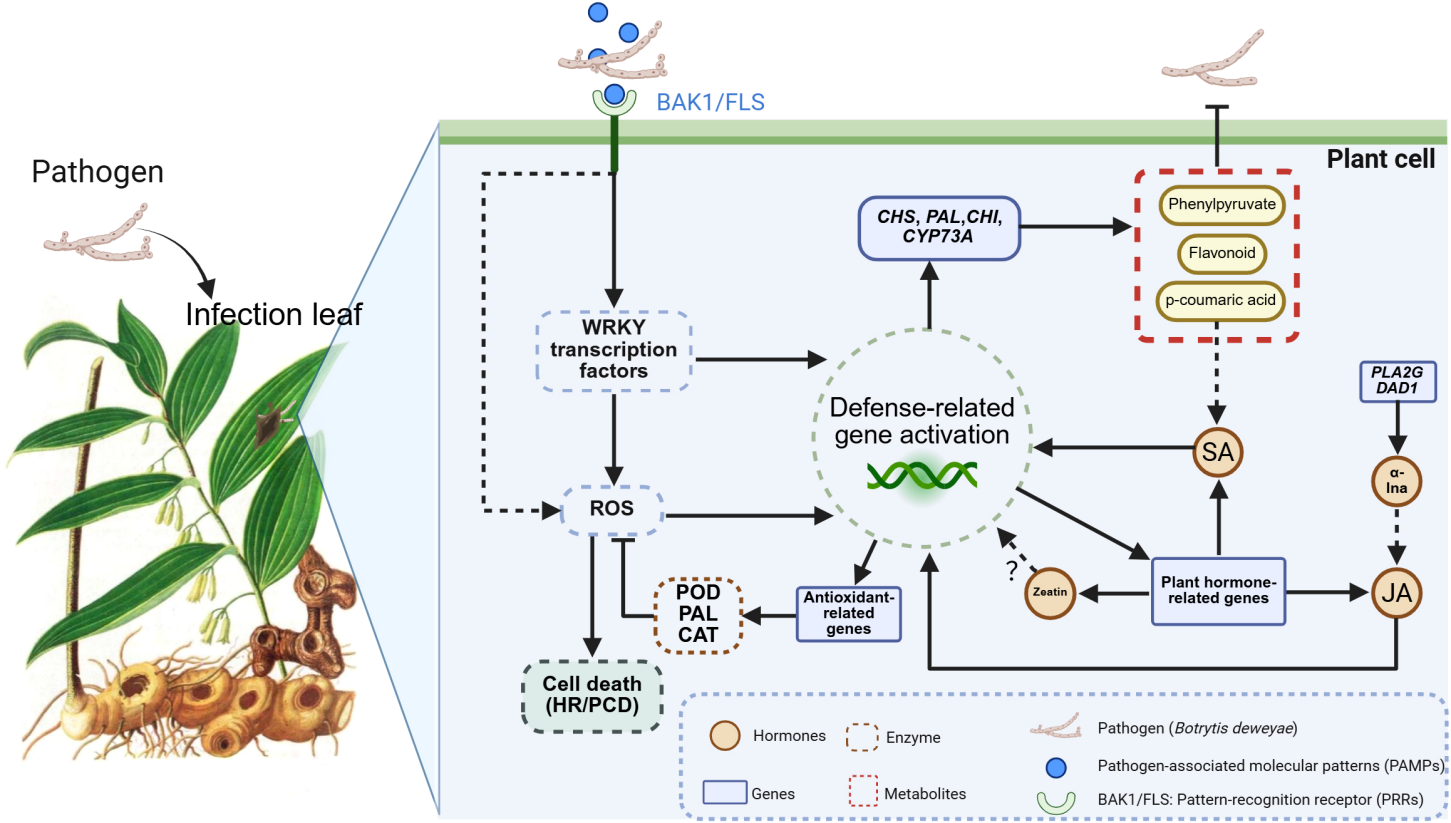
Proposed model for leaf of *Polygonatum cyrtonema* in response to *Botrytis deweyae* infection. (PRR)-triggered immunity; ROS, reactive oxygen species; PCD, programmed cell death; HR, hypersensitive response; POD, peroxidase; PAL, phenylalanine deaminase; CAT, catalase, *CHI*, chalcone isomerase; *CHS*, chalcone synthase; *CYP73A*, cinnamate 4-Hydroxylase; *PLA2G*, phospholipase A2 group; *DAD1*, defender against cell death 1; JA, jasmonic acid; SA, salicylic acid; α-LnA, α-linolenic acid.

In this study, when *P. cyrtonema* was subjected to *B. deweyae* infection, and two receptor protein kinase genes, BAK 1 and FLS 2 (PRRs) on the cell membrane may perceive *B. deweyae*-related effector molecules and potentially initiate MAPK signaling, as suggested by upregulated MAPK gene expression in our transcriptome data, which includes the activation of downstream disease resistance genes by the transcription factor WRKY and the induction of PCD and HR through the burst of ROS (**Figure 14**). WRKY is the second most important transcription factor in disease resistance responses after AP2ERF-ERF. Under *B. deweyae* stress, ROS accumulation and WRKY 33 may activation-induced expression of phytohormone-related genes involving WRKY 22 require the mediation of the WRKY transcription factor gene family. Silencing the FaWRKY 25 gene can increase JA biosynthesis and increase resistance to *B. cinerea* in strawberries (Jia et al., 2020), and overexpressing FaWRKY 25 leads to a decrease in resistance. However, the silencing of WRKY-related genes in *Nicotiana benthamiana* reduces resistance to *B. cinerea* (Ramos et al., 2021). Although the overexpression of WRKY TFs can activate the production of downstream disease-resistant metabolites, for necrotrophic fungi such as *Botrytis* spp (Saha et al., 2023), the PCD response of overexpressed WRKY TFs is more advantageous for infection, indicating that there are significant differences in disease resistance responses among different types of fungi when facing gray mold infection. The specific functions of the WRKY 33 and WRKY 22 transcription factor family members identified in this study need to be further investigated.

The invasion of *B. deweyae* led to an increase in defense enzyme activity in *P. cyrtonema* leaves to different degrees, and all of these activities were greater than those in the control group. CAT activity reached its peak at 84 h and then rapidly decreased at 96 h. The PAL and SOD enzyme activities were optimal between 72 and 84 h, whereas the POD enzyme activity was the best between 60 and 72 h. Compared to the disease progression of inoculated leaves, as the leaves turn yellow and wither, the activity of protective enzymes decreases (**Figure 2**). Moreover, multiple DEGs were enriched in the peroxisomal transcriptome, and these genes have antioxidant functions according to the KEGG database. In MF to GO enrichment, there were 13 peroxidase activities (**Figure 5**), four phenylalanine oxidase activities, two SOD enzyme activities, and two CAT enzyme activities. CAT is present in animals, plants, and microorganisms, and the overexpression of CAT genes can significantly increase the disease resistance of potatoes (**Figure 6**). In tobacco lacking CAT genes, cells undergo apoptosis due to an increase in hydrogen peroxide levels (Adachi et al., 2016). Besides participating in the antioxidant process, POD enzymes can also catalyze the production of phenolic compounds that are toxic to pathogens or synthesize lignin to form a physical barrier. The activity of POD enzymes is positively correlated with the disease resistance of the host and the overexpression of POD genes in plants such as tea and tobacco can improve disease resistance (Lai et al., 2016). PAL is a key and rate-limiting enzyme in the phenylpropionamide metabolic pathway, providing precursors for the synthesis of phytohormones (Lin et al., 2018), such as flavonoids and terpenoids. The enrichment results of the 189 coexpressed genes indicate that the PAL gene is involved in the biosynthesis of terpenoids such as phenylpropanoids, flavonoids, and quinones. In this study, during *B. deweyae* infection, multiple POD genes were upregulated and participated in the disease resistance process. These genes played a dominant role, and further research is needed to determine the functions of these genes.

We analyzed the PPI network of DEGs in the transcriptome and revealed that the DN1274_c1_g1 gene of ubiquitin-2, like Rad60 SUMO-like, interacts with multiple functional genes. DN1274_c1_g1 is annotated as a ubiquitin-like domain of the ubiquitinoid miniature modifier (**Figure 8**). The ubiquitin-like domain (ULD) is part of a polypeptide, and its primary function is to mediate protein hydrolysis. Ubiquitinoids are a family of proteins with key structural characteristics similar to those of ubiquitin. Studies on the class of ubiquitinoid protease genes under biological stress conditions are limited, whereas CRL-type and RING/U-box-type E3 ubiquitin ligases have been extensively studied in the context of plant disease resistance (Hu et al., 2014). In this study, the interaction between DN1274_c1_g1 and the gene regulating sphingolipid metabolism, DN65_c0_g 1, was the strongest, and their coexpressed KEGG enrichment analysis revealed that DN1274_ c1_ g 1 was associated with sphingolipid metabolism. Sphingolipids are signaling molecules that can trigger PCD in plants and are involved in various cellular processes, such as apoptosis, cell proliferation, and autophagy (Mu et al., 2024). Along with sphingolipid metabolism, multiple disease resistance signaling pathways are regulated by the ubiquitination gene DN1274_c1_g1, including peroxisomes, plant hormone signaling, and α-linolenic acid, indicating that the response of *B. deweyae* to *P. cyrtonema* is regulated by ubiquitinoid-like genes. Although our integrative analysis reveals candidate regulators and metabolites potentially involved in *P. cyrtonema*’s defense, further functional validation through gene silencing or overexpression is required to confirm causality.

The results of the comparative metabolome analysis revealed that the gene and metabolic levels of phenylpropanoid synthesis and α-linolenic acid metabolism significantly increased after *B. deweyae* infection (**Figure 11**). The phenylpropanoid metabolic pathway is an important pathway involved in plant basal disease resistance, and its downstream products play crucial roles in resisting *B. deweyae* infection and self-antioxidation processes, which participate in the resistance of *P. cyrtonema* against *B. deweyae* (**Figure 14**), whereas other phenylpropanoid metabolites indirectly activate the SA-mediated disease resistance response, activating the transcription of disease resistance genes to prevent further invasion by *B. deweyae*. Furthermore, in the KEGG pathway, α-linolenic acid metabolism and phenylpropanoid biosynthesis are mediated by activating hormone signaling to facilitate disease resistance processes, specifically the transcription of downstream disease resistance genes mediated by SA and the defense regulatory mechanisms mediated by JA. Genes and metabolites involved in α-linolenic acid metabolism remain highly active throughout the infection process. The production of LA affects the production and colonization of *Aspergillus* sp. spores (Upchurch, 2008). SA can activate the expression of plant disease process-related proteins and regulate the activity of disease-resistance proteins to improve plant resistance (Mishra et al., 2024). The exogenous application of SA can inhibit the germination of rice blast spores and reduce the occurrence of rice blast disease (Wang et al., 2011). These findings may suggest that *P. cyrtonema* could synthesize bioactive antifungal compounds through pathways such as phenylpropanoid and α-linolenic acid metabolism, potentially inhibiting the growth and development of *B. deweyae* mycelia. Tissue-specific and low-abundance responses may have been underrepresented due to the limitations of bulk RNA-seq and untargeted metabolomics. Thus, further validation through overexpression or silencing of key genes is required to confirm the underlying mechanisms.

The interaction network diagram of genes and metabolites revealed that genes regulating zeaxanthin synthesis are also involved in JA biosynthesis (**Figure 11**). Zeatin is a plant growth regulator that plays a significant role in regulating plant growth, development, and stress resistance. Whether there is a synergistic effect between zeatin and JA in the disease resistance process needs to be investigated (**Figure 14**). Although untargeted metabolomics identified significant alterations in phytohormones (e.g., JA, zeatin) in *P. cyrtonema* following *B. deweyae* infection, these findings remain provisional due to methodological limitations. To enhance reliability, future studies will employ targeted LC-MS/MS with authentic standards for precise quantification of these hormones. Additionally, the metabolism of sphingolipid and sphingomyron is enriched in the transcriptome and metabolome, and whether these genes participate in activating the PCD mechanism of *P. cyrtonema* requires further assessment. These findings provide foundational insights that may support the development of disease-resistant cultivars or biostimulant strategies for *P. cyrtonema* and related medicinal plants.

## 4 Materials and methods

### 4.1 Sample preparation

Tubers of identical age and variety were collected from the *P. cyrtonema* plantation in Longju Town, Wanzhou District, Chongqing (30ngqingnn 108ngqingnnse Three independent biological replicates (n=3) were established, with each replicate representing one individual plant. After treatment with 50% carbendazim, plants were allowed to grow for three months, three fully expanded leaves per plant were uniformly inoculated by creating standardized micro-wounds using sterile needles (0.3 mm diameter). Three precisely positioned wounds were introduced per leaf, inoculated with inverted 5-mm fungal cakes, and wrapped with cling film. Tissue from the lesion margins of these three leaves was pooled to create one composite biological sample per replicate plant. All plants were maintained in a greenhouse (95% ± 1% RH; 27 ± 1°C) under 12-h light/dark cycles.

### 4.2 Defense enzyme extraction and activity determination

For each harvest time point (0, 12, 24, 36, 48, 60, 72, 84, and 96 hours post-inoculation), three independent biological replicates were collected (n=3 per time point). Each biological replicate consisted of lesion-edge tissue pooled from three inoculated leaves of a single *P. cyrtonema* plant. The activities of the three enzymes were determined by the BC 0205 kit for catalase (CAT), the BC 0215 kit for phenylalanine deaminase (PAL), and the BC 0095 kit for peroxidase (POD) provided by Solarbio Science & Technology Co., Ltd. (Beijing). The measured data were processed and statistic ally analyzed using Excel 2016 and SPSS 20.0 software, differences between inoculated plants (treatment) and uninoculated controls were analyzed using welch two-sample t-tests. Temporal changes within the treatment group across post-inoculation time points (0–96 h) were assessed via one-way ANOVA with Tukey’s HSD *post hoc* test (α = 0.05), and the graphs were plotted using GraphPad Prism 8. Enzyme activities were calculated as follows:


$$CAT\;activity(U/g\;FW)=[\frac{\Delta A_{240}\;\times \;Vt}{\left(\epsilon \;\times \;d\right)}\times 10^{6}]÷(\frac{Vs}{Ve\;\times \;W})÷T$$

$$PAL\;activity(U/g\;FW)=\Delta A_{290}\times Vt÷0.1÷(\frac{Vs}{Ve\;\times \;W})÷T$$

$$POD\;activity(U/g\;FW)=\Delta A_{470}÷0.01\times Vt÷(\frac{W}{Ve\;\times \;Vs})÷T$$

U/g FW: One unit of enzyme activity (U) is defined as the amount of enzyme that catalyzes the degradation of 1 μmol of substrate per minute per gram of tissue under standard assay conditions.

FW: Fresh weight; ΔA: Change in absorbance; Vt: Total reaction volume (mL); Vs: Volume of enzyme extract added (mL).

Ve: Total extraction volume (mL); T: Reaction time (min); W: Sample fresh weight (g).

### 4.3 Transcriptome sequencing analysis

#### 4.3.1 Sample collection

Samples were collected at 0 h, 24 h, 48 h, and 96 h after inoculation, with healthy tissues from the interface between diseased and healthy tissues (3 × 5 mm) collected in centrifuge tubes and stored in liquid nitrogen. Metabolomic samples were preserved using the same method, while transcriptomic analysis was performed on three biological replicates per group (n=3), and metabolomic analysis was performed on biological replicates per group (n=4). The repetitive groups and grouping of each sample are listed in **Supplementary Table S2**.

#### 4.3.2 RNA extraction and quantitative detection

Total RNA was extracted from the samples using an RNAprep Pure Plant Kit (Tiangen, Beijing, China). The RNA concentration and purity were measured using a NanoDrop 2000 system (Thermo Fisher Scientific, Wilmington, DE). The integrity of the RNA was assessed using an RNA Nano 6000 detection kit from Agilent Bioanalyzer 2100 Systems (Agilent Technologies, CA, USA). After confirming that the RNA was sufficient for detection, one part was used for sequencing, and the other part was used for qPCR validation.

#### 4.3.3 Sequence acquisition and annotation

Paired-end 150 bp (PE 150) mode sequencing was performed using the Illumina NovaSeq 6000 sequencing platform. Gene functions were annotated by comparing with the KOG/COG (Clusters of Orthologous Groups of proteins), KO (KEGG Ortholog database), and GO databases. Data were analyzed using the bioinformatics analysis workflow provided by the Biomarker Cloud Platform BMKCloud (www.biocloud.net).

#### 4.3.4 Differential expression gene screening

Genes whose expression levels significantly differ among different samples are called DEGs, and the set of genes obtained from differential expression analysis is known as the set of DEGs, referred to as “A vs. B” (A is the control and B is the experimental treatment). The grouping of DEGs identified in this study is shown in **Supplementary Table S3**.

Differential expression gene screening was performed using differential analysis software based on the count value of genes in each sample, and DESeq 2 was used (Love et al., 2014). While detecting DEGs, the screening criteria were set as a differential expression ratio (fold change) ≥ 2 and a false discovery rate (FDR) < 0.01. The greater the absolute value of log_2_FC, the more significant the difference between the two groups of samples of genes with smaller FDR values.

#### 4.3.5 Differential gene enrichment analysis

The KEGG database resources (Kanehisa et al., 2011) (http://www.kegg.jp), and the KOBAS database (Xie et al., 2011) were used to conduct DEGs enrichment analysis. The clusterProfiler software was used to analyze the enrichment of DEGs in KEGG pathways, and the GO enrichment analysis of DEGs was performed using the clusterProfiler package based on Wallenius non-central hypergeometric distribution (Wu et al., 2021), with P < 0.05 as the threshold. Combined with the differential expression analysis results, the differential gene sequences were aligned to the STRING database (http://stringdb.org/) to obtain the protein-protein interaction (PPI) relationships of the DEGs in *B. deweyae*, which were then visualized using Cytoscape 3.10.1 (Shannon et al., 2003).

#### 4.3.6 Differential gene qPCR verification

The DEGs used for qPCR validation were selected from the original material that passed detection for reverse transcription. The reverse transcription reaction was performed with the MightyScript First Chain cDNA Synthesis Master Mix (B639251) kit, following instructions provided by Shanghai Biotech Co., Ltd. (MightyScript). After the reaction ended, the samples were stored at –20°C until use. Primers were designed using Primer Premier 6 software with target gene band sets between 150–250 bp, and the gene sequences are shown in **Supplementary Table S4**. The internal reference gene of ubiquitin-conjugating enzyme-E2-10 (UBQ-E2-10) and elongation factor 1-alpha isoform (EF-1α2) were selected to correct the relative expression levels of the genes. The Q-PCR mixture was prepared as follows: 20 μL, including 10 μL of Master premix (SGE xcel FastSYBR, B 532955, Sangon Biotech), 0.3 μL each of the upstream and downstream primers, and 2 μL of cDNA, using RNase-Free ddH_2_O, was added to 20 μL, and three repetitions were set for each sample. Real-time fluorescence qPCR was performed on a Gentier 96E (Xi’an Tianlong Technology Co., Ltd.).

The one-step real-time fluorescence qPCR protocol was as follows: predenaturation at 95°C for 15 s, reaction at 95°C for 5 s, and reaction at 58°C for 30 s for 38 cycles; the melting curve program was as follows: 95°C for 15 s, followed by 60°C for 1 min. If the Ct value was between 15 and 25, the melting curve was a single peak, and if the Tm value was between 80 and 90, the target gene was considered to be successfully amplified. After obtaining the data using Gentier 96E, the software provided by Gentier 96E was used to preliminarily collate and analyze the experimental data. The 2^-ΔΔCt^ method was used to obtain the relative expression value, and the calculation formula was as follows: The graphs were drawn with GraphPad Prism 8.

ΔΔ Ct = (Ct of the target gene in the experimental group-Ct of the internal reference gene in the experimental group) - (Ct of the target gene in the control group-Ct of the internal reference gene in the control group).

### 4.4 Metabolomic profiling methods

Untargeted metabolomic profiling of all 16 samples was performed using an LC-QTOF platform. The LC/MS system for metabolomics analysis is composed of Waters Acquity I-Class PLUS ultra-high performance liquid tandem Waters Xevo G2-XS QTof high resolution mass spectrometer. Mass spectrometry data were acquired in MSe mode under the control of the MassLynx V 4.2 (Waters) software for high-resolution mass spectrometry (Wang et al., 2016). Data processing operations such as peak extraction and peak alignment were performed using the Progenesis QI software, and identification was conducted based on the online METLIN database, public database, and Baimaike self-built database.

The metabolome was functionally annotated using spectral libraries (Mass Bank METLIN NIST, etc.), metabolic pathway databases (KEGG PlantCyc MetaCyc), compound information databases (PubChem ChemSpider, etc.), and metabolomics experimental information management databases (SetuoX SesameLIMS). The classification and information on the pathway of the identified compounds were searched in the KEGG HMDB and lipidome databases.

DAMs were grouped using the “A vs B” method (A as the control and B as the experimental treatment) to screen for DAMs, with samples divided into groups for differential comparison (**Supplementary Table S5**). The grouping information showed that the fold changes (FC) were calculated and compared, and a t-test was conducted to determine the differences for each compound. The ropls package in Rstudio was used for orthogonal projections to latent structures- discriminant analysis (OPLS-DA) modeling to verified through permutation testing (n = 200). Briefly, class labels were randomly shuffled, and new OPLS-DA models were reconstructed using the permuted groupings. The *R^2^Y* (goodness-of-fit) and *Q^2^Y* (predictive ability) values from each permuted model were recorded. These values were plotted against those of the original model in a scatter plot to confirm that the original model’s performance exceeded random chance (p < 0.05). Differential multiples from the OPLS-DA model were used as screening criteria with the following conditions: FC ≥ 2, p-value < 0.05, and Variable Importance in Projection (VIP) > 1. Hypergeometric distribution testing was conducted to calculate the enrichment significance of DAMs in KEGG pathways. The screened DAMs were mapped to the KEGG network database for comparison, and the metabolites with the highest degree of matching for metabolic pathway annotation and analysis were selected.

### 4.5 Association analysis between the transcriptome and metabolome

To establish functional linkages between genes and metabolites, we performed rigorous correlation analyses by integrating transcriptomic and metabolomic datasets, the comparison group information is provided in **Supplementary Table S6**. Following UV-scaling pretreatment of KEGG-annotated differentially expressed genes (DEGs) and differentially accumulated metabolites (DAMs), two-way orthogonal partial least squares (O2PLS) analysis was conducted using the OmicsPLS package in RStudio to model intrinsic correlations between the two datasets. Principal component analysis (PCA) was additionally employed to evaluate sample dispersion patterns across both transcriptomic and metabolomic datasets, providing complementary assessment of data structure variation. KEGG pathway enrichment analysis for both metabolomic and transcriptomic data was performed using Fisher’s exact test. The resulting p-values were adjusted via the Bonferroni correction method, with a corrected p-value (p adjust) ≤ 0.05 considered statistically significant for pathway enrichment. Based on KEGG enrichment pathways, we generated distinct bar charts and bubble plots for transcriptomic and metabolomic datasets, respectively. The pathway mapping of DEGs and DAMs was visualized using component information extracted from the KEGG Markup Language. Subsequently, Pearson correlation coefficients (PCC) were calculated between all differentially expressed genes and metabolites across comparison groups. Networks were filtered using dual thresholds: |PCC| > 0.80 with corresponding correlation p-value < 0.05. we constructed an interaction network of JA pathway-related genes and metabolites using Cytoscape software (version 3.10.1), and a hypothetical model diagram of *P. cyrtonema* in response to *B. deweyae* infection using the illustrations software https://BioRender.com).

## Data Availability

The datasets generated or analyzed during the current study are available in the National Center for Biotechnology Information (NCBI) database under the BioProject ID PRJNA1115085. https://www.ncbi.nlm.nih.gov/sra/PRJNA1235604, The Metabolome datasets generated or analyzed during the current study are available in the China National Center for Bioinformatics (CNCB) database under the BioProject ID PRJCA038870.
