# Supplementary material for: Combined transcriptome and metabolome analysis of Polygonatum cyrtonema Hua in response to Botrytis deweyae infection
Source: Front Plant Sci. 2025 Jul 29;16:1617308. doi: 10.3389/fpls.2025.1617308 (PMC12339532; doi:10.3389/fpls.2025.1617308)
Supplement: Supplementary file 1 [file DataSheet1.docx]

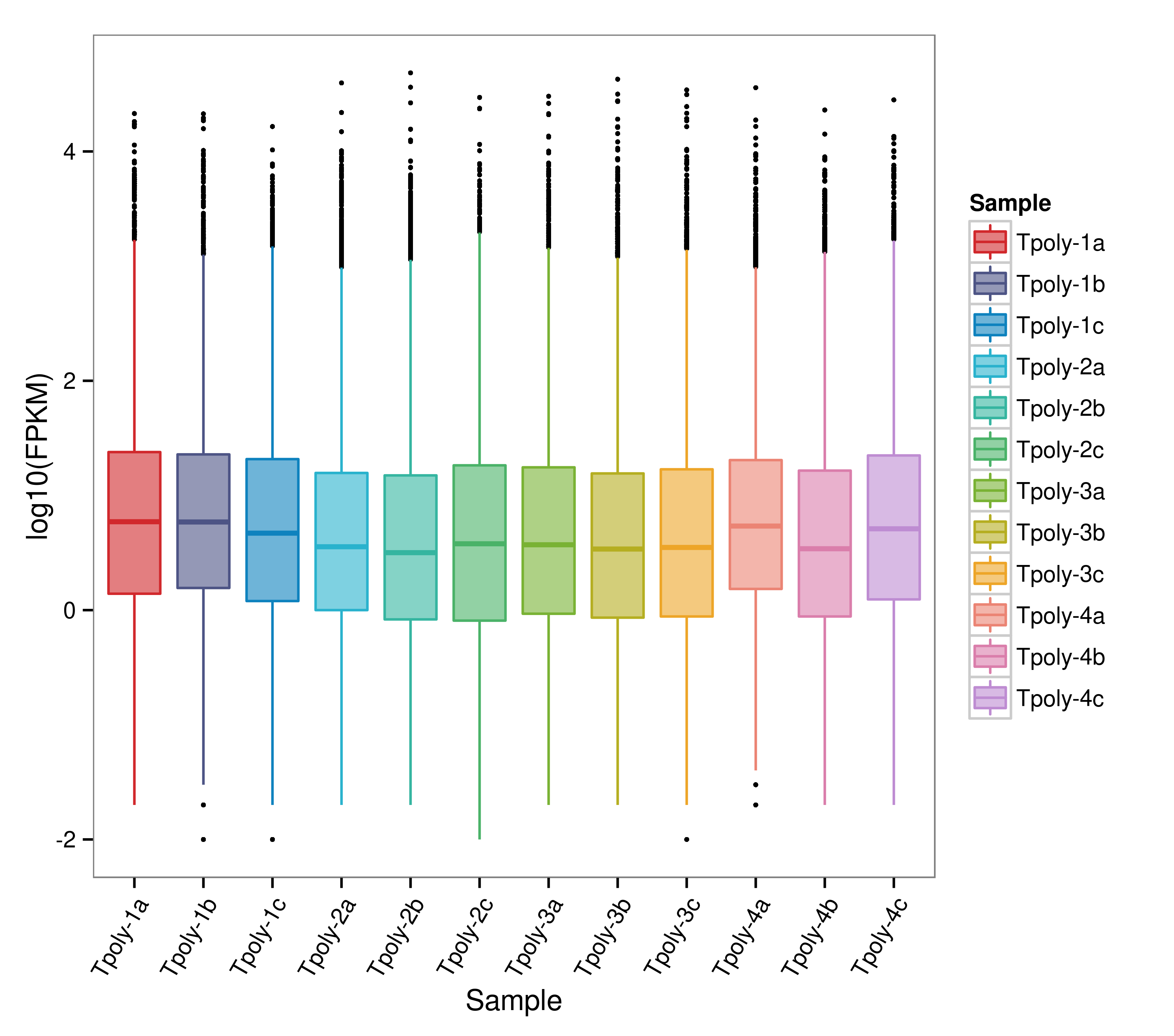


**A**


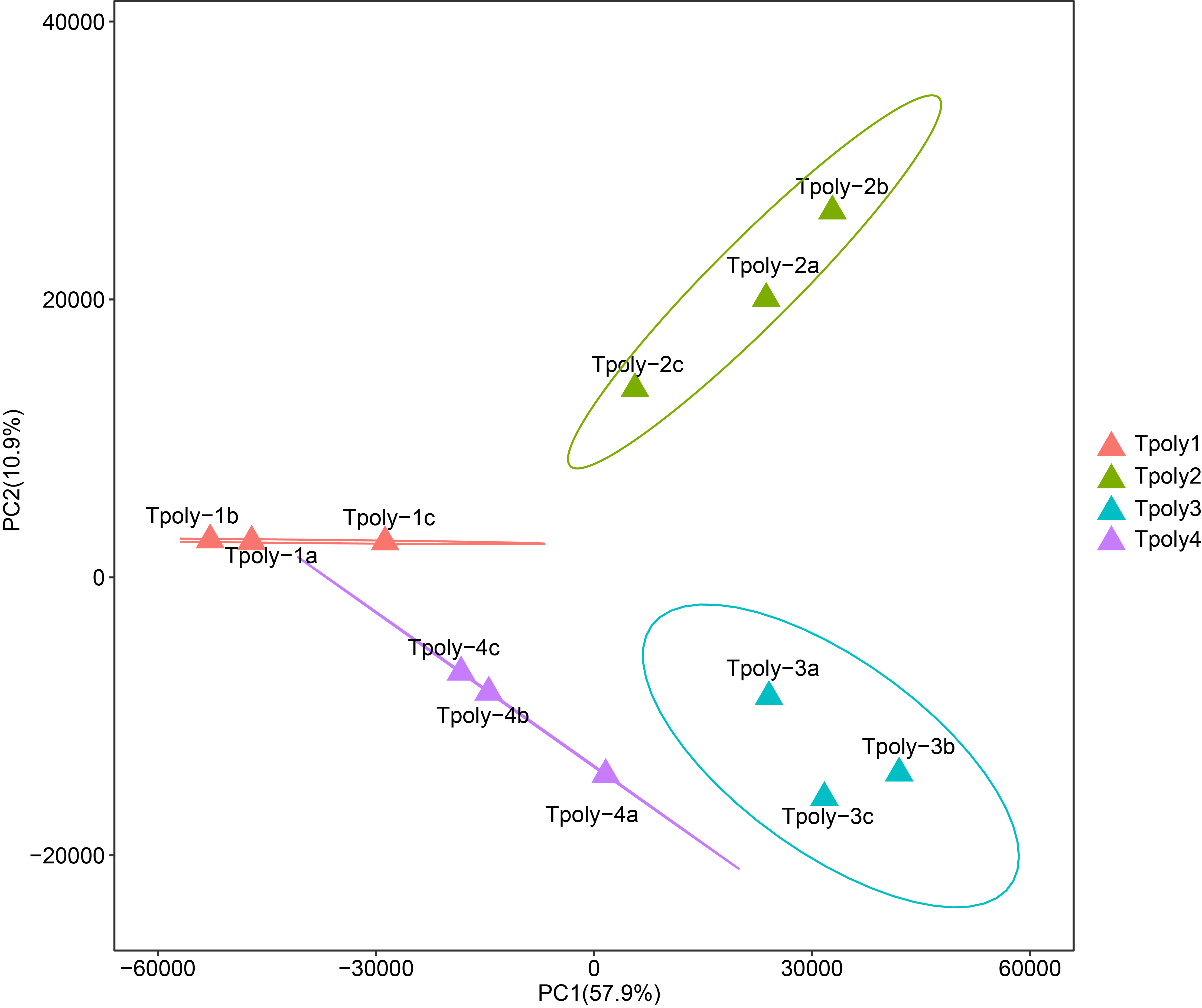


**B**

Figure. S1 Transcriptome gene expression levels and correlation of *Polygonatum cyrtonema* leaves

A. Boxplot of expression levels for each sample; B. Sample correlation PCA analysis


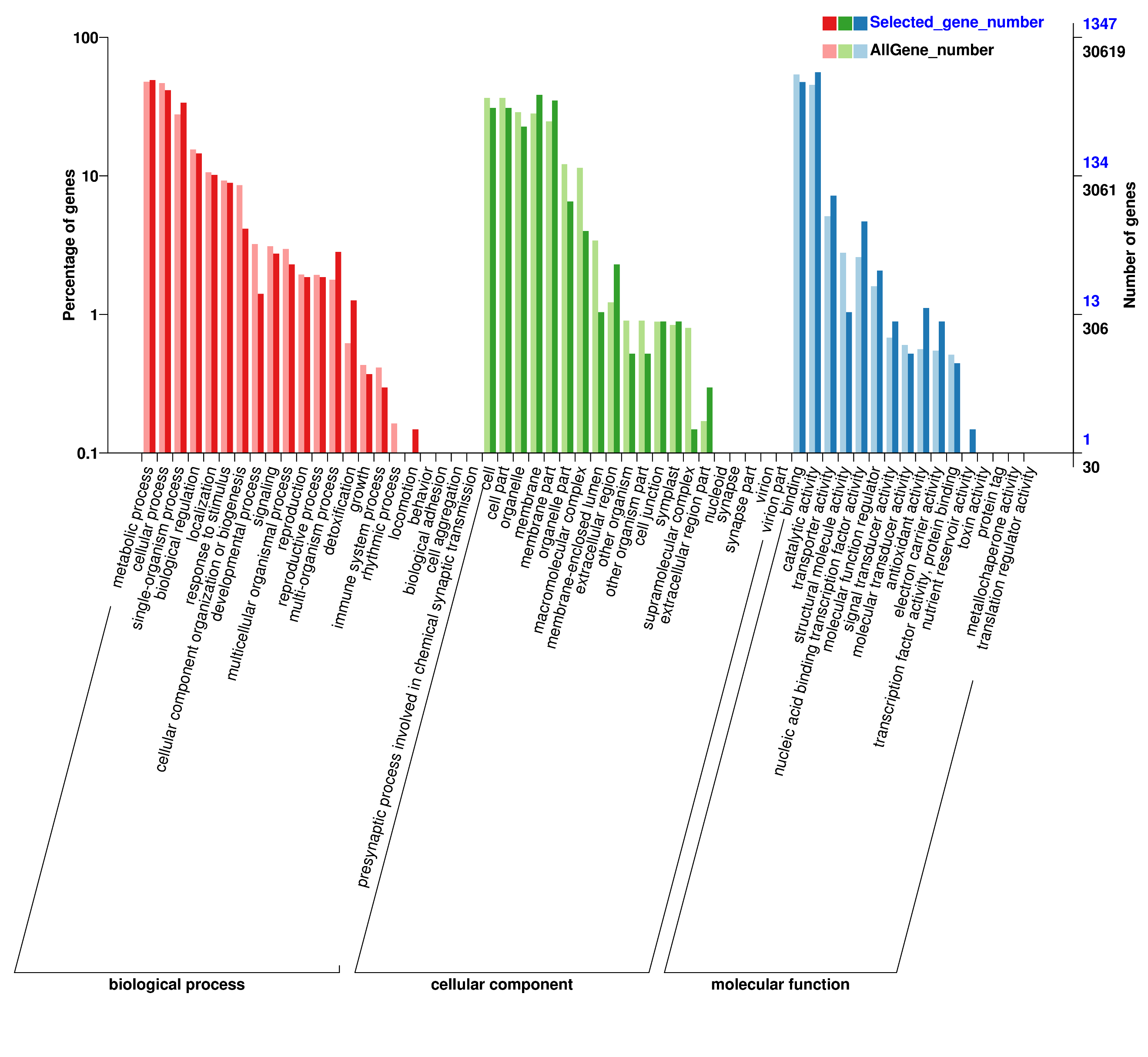


Fig. S2 Classification and enrichment of GO. The abscissa represents the pathway annotated through the Gene Ontology database, the red (biological processes), green (cellular component), and blue (molecular function), and the Ordinate represents the number or proportion of genes corresponding to them.


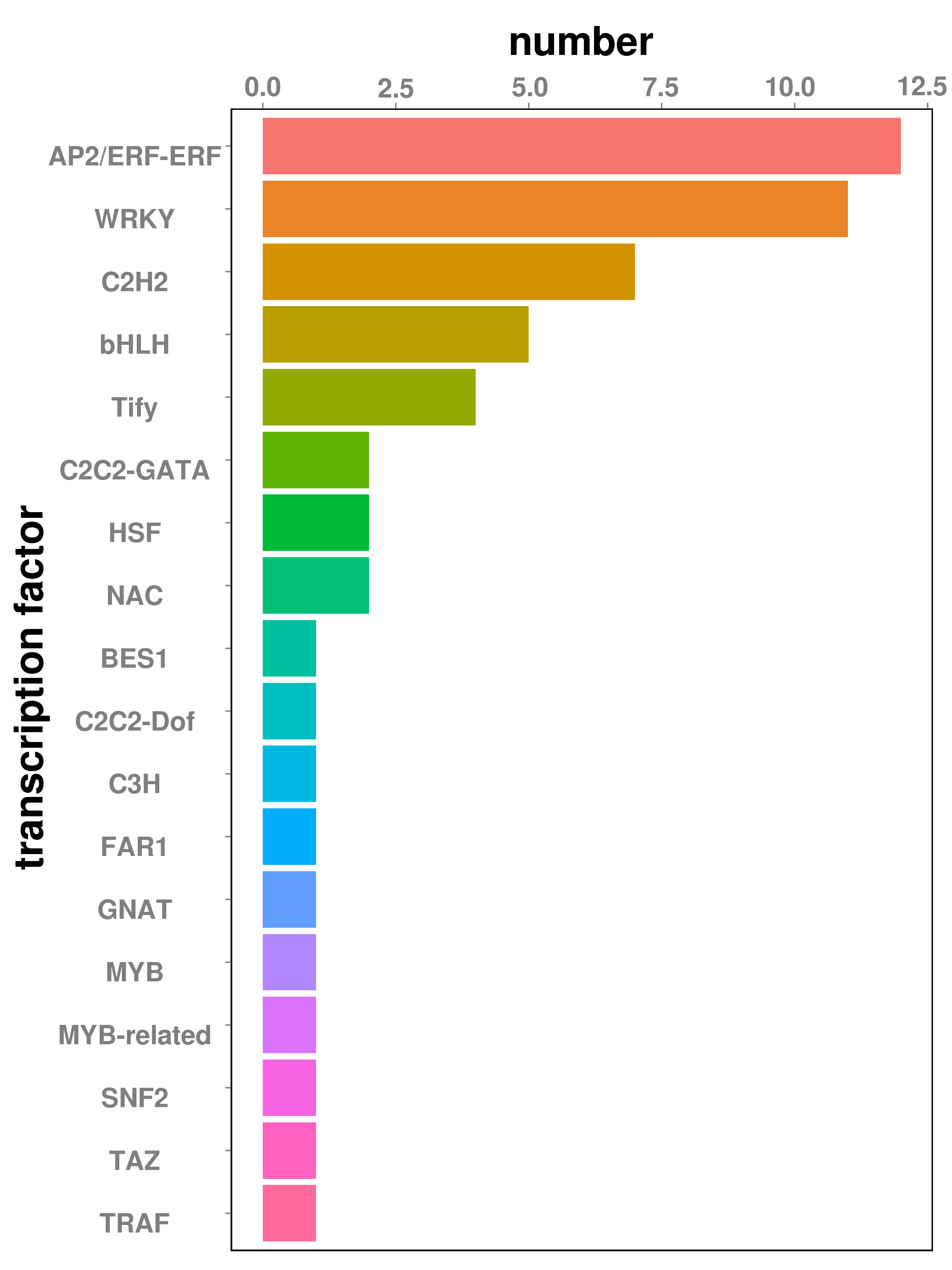


A


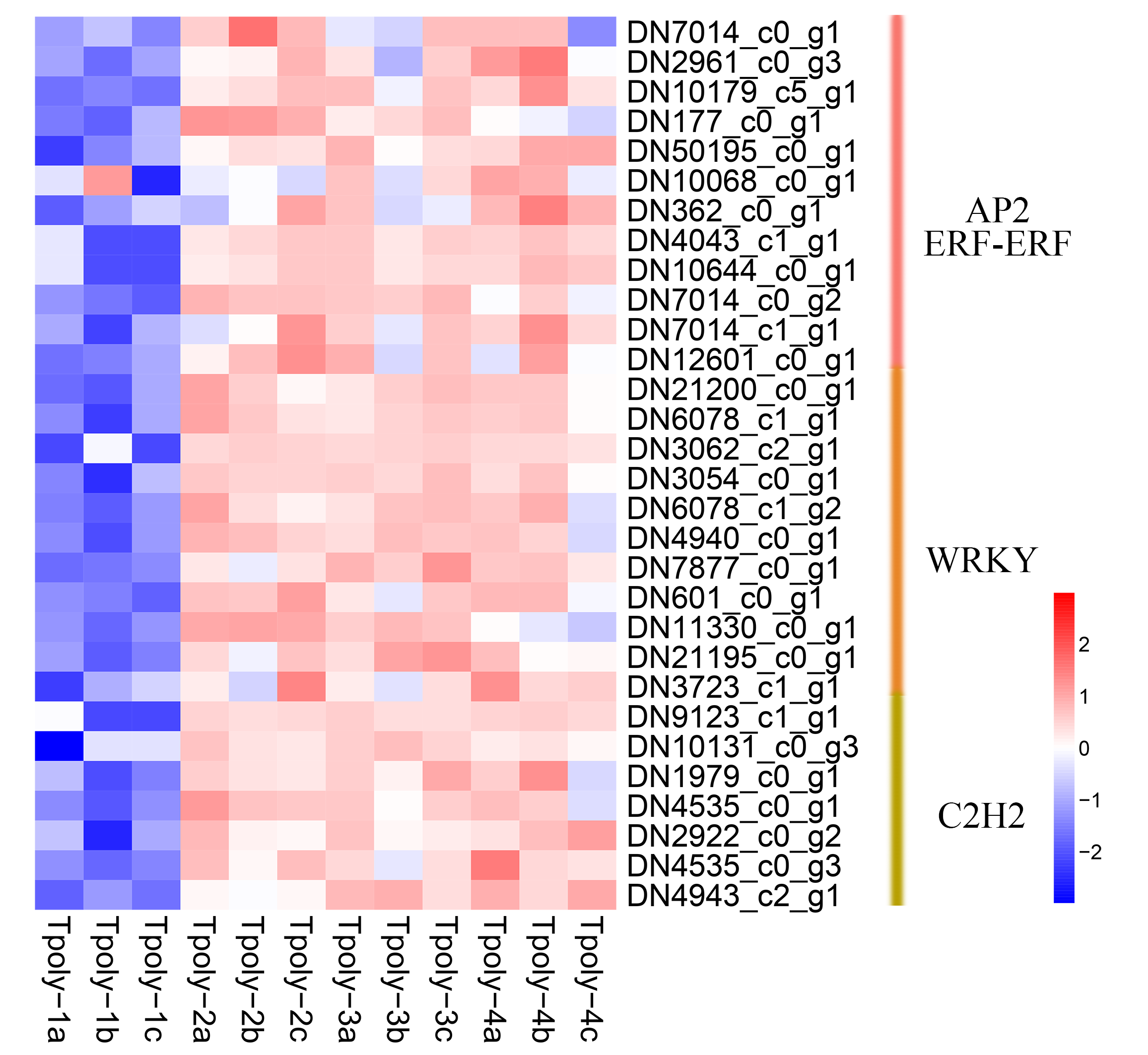


B

**Figure S3.** Transcription factor prediction and heatmap. A. S.tatistics on the number of transcription factors. The abscissa represents the number of genes, and the ordinate represents the transcription factor to which it is annotated; B. Gene heatmap among the top 3 transcription factors. The redder the color, the more enriched the gene is in that sample.

**
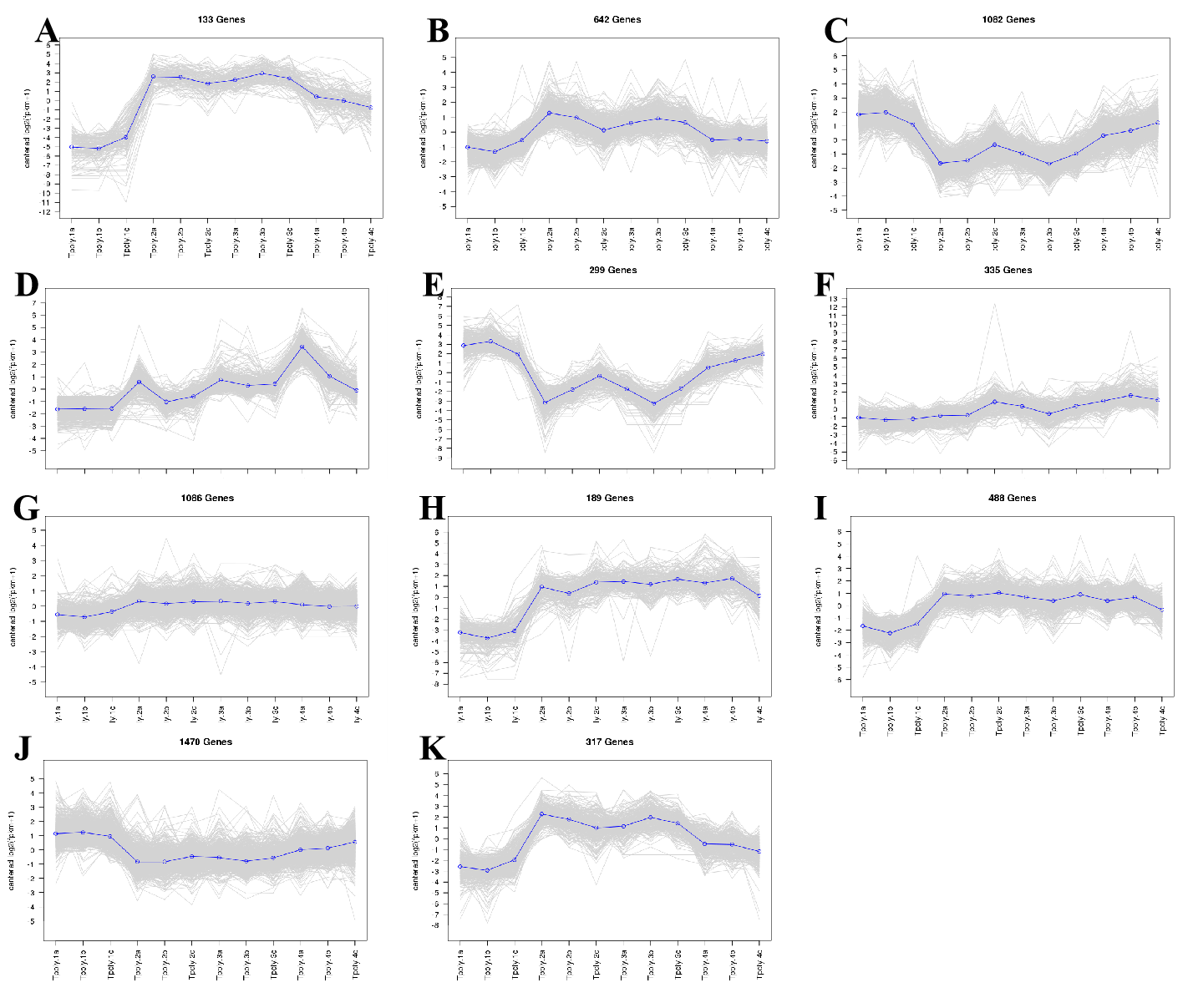
**

**Fig S4.** 11 co-expressed trends in transcriptome sequence of *Polygonatum cyrtonema*.


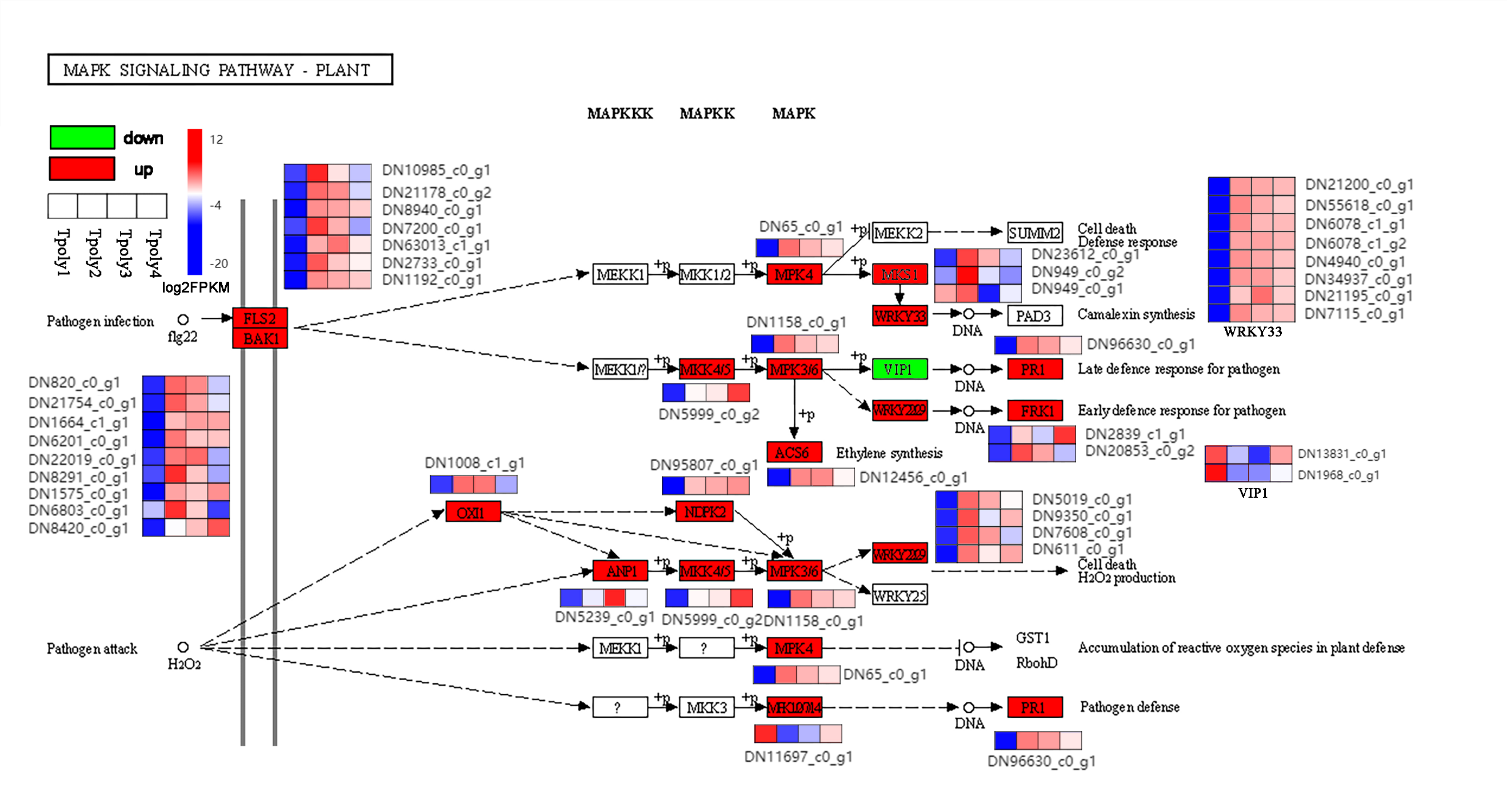


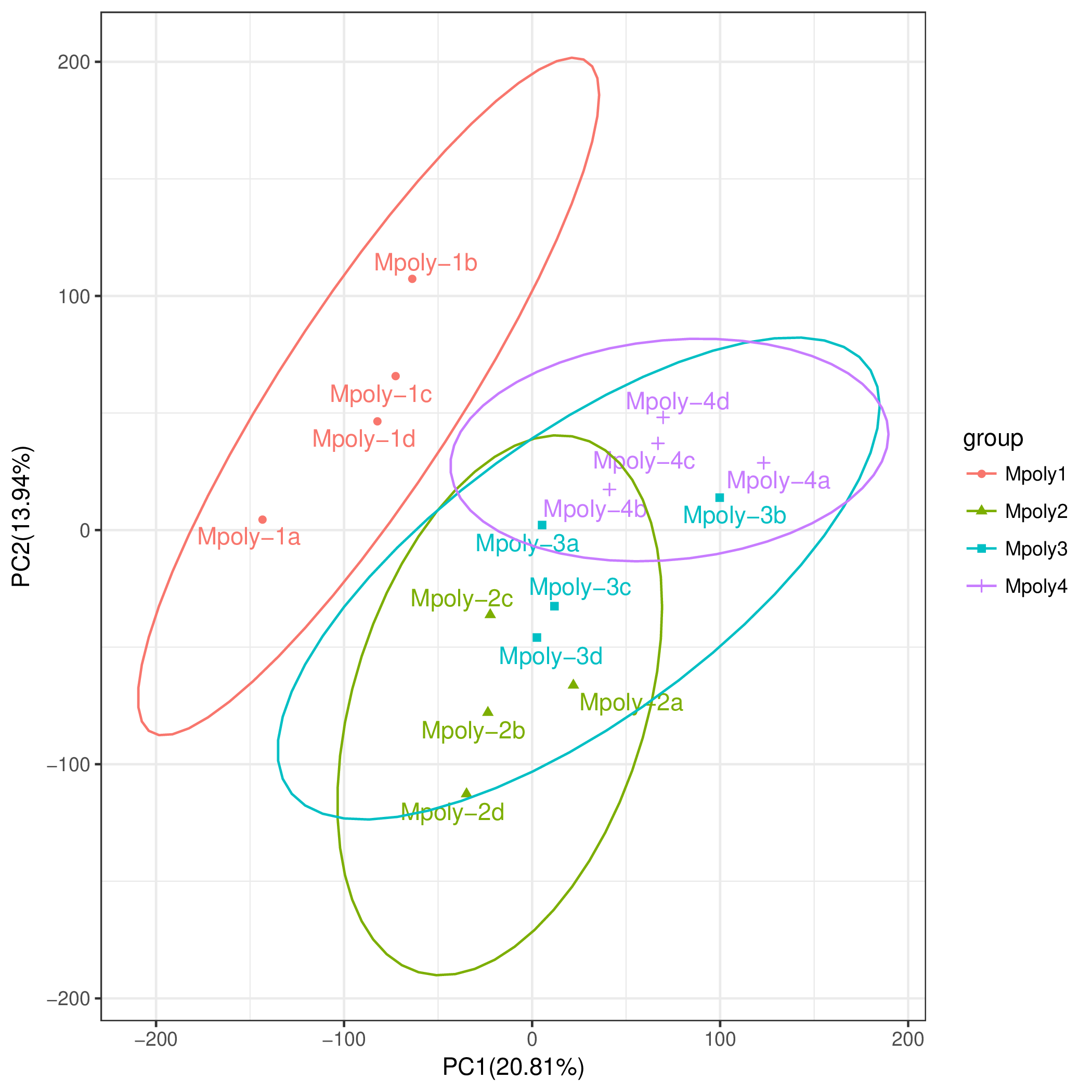


**A**


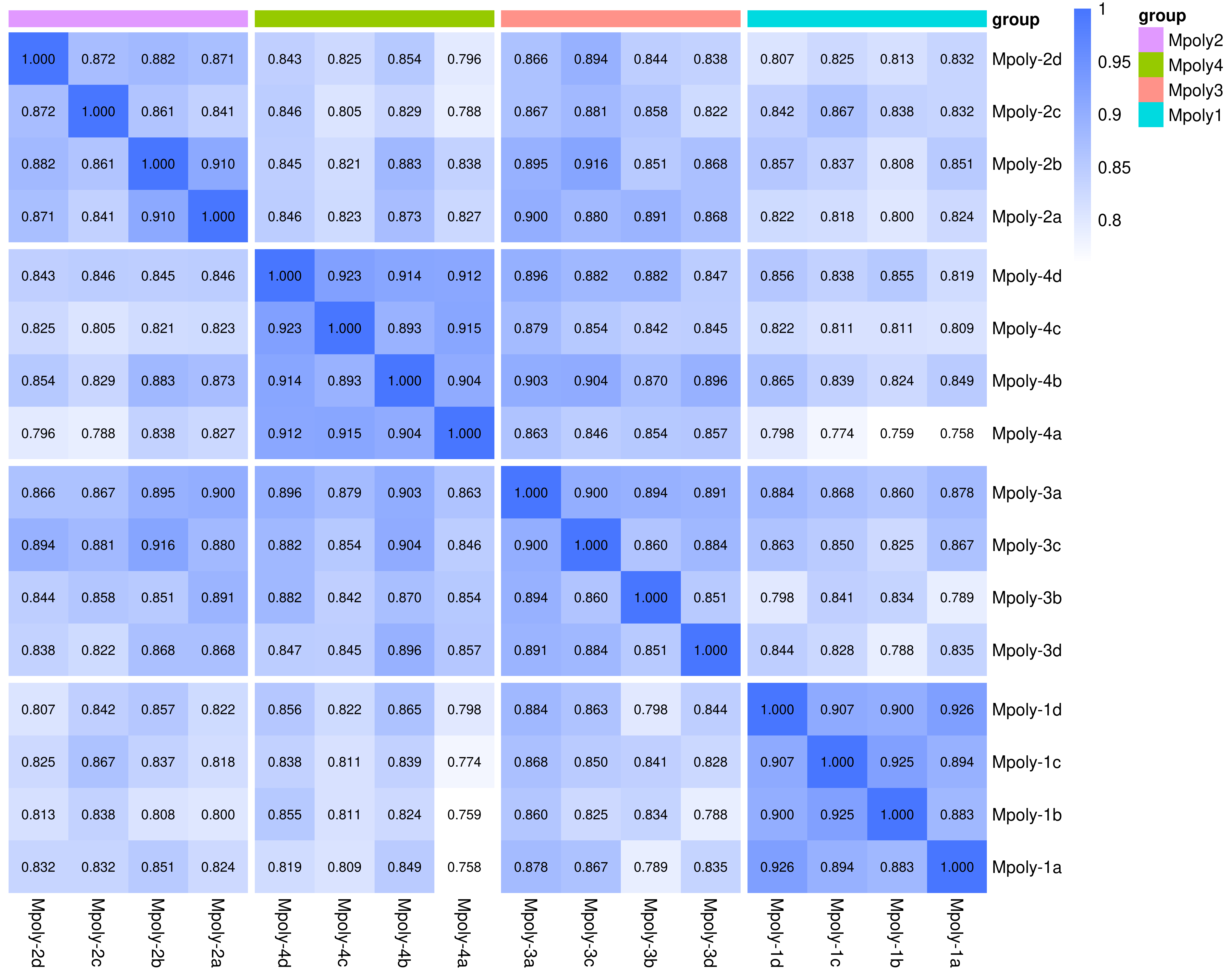


**B**

**Fig. S6** Correlation evaluation of various samples in the metabolome

A. Principal component analysis of differential grouping; B. Correlation graph between samples

**Fig. S5** MAPK activated disease resistance response. Each gene is represented in the wireframe, the red shading represents the up-regulation of the gene, the green represents the down-regulation, and the heat map represents the enrichment level of the gene at four time points, and the redder the color indicates the more enrichment of the gene at that time point.


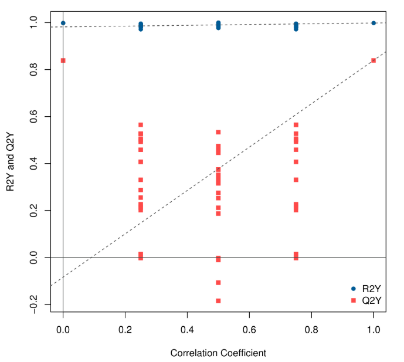

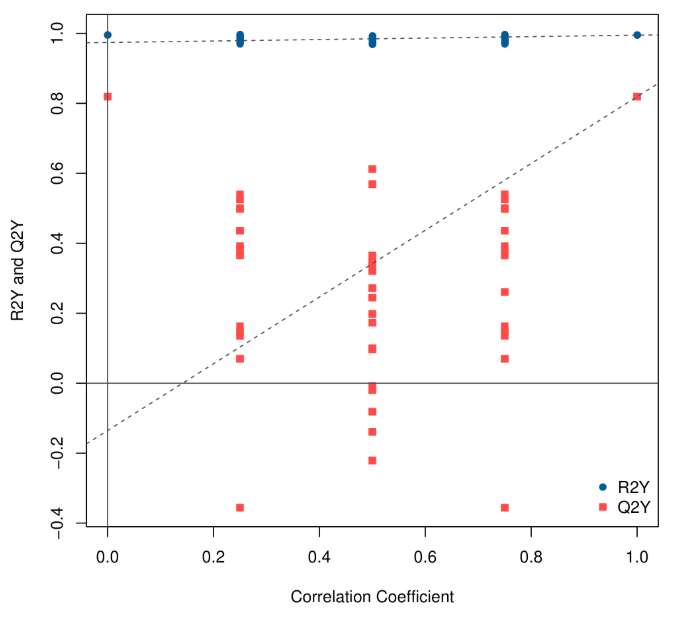

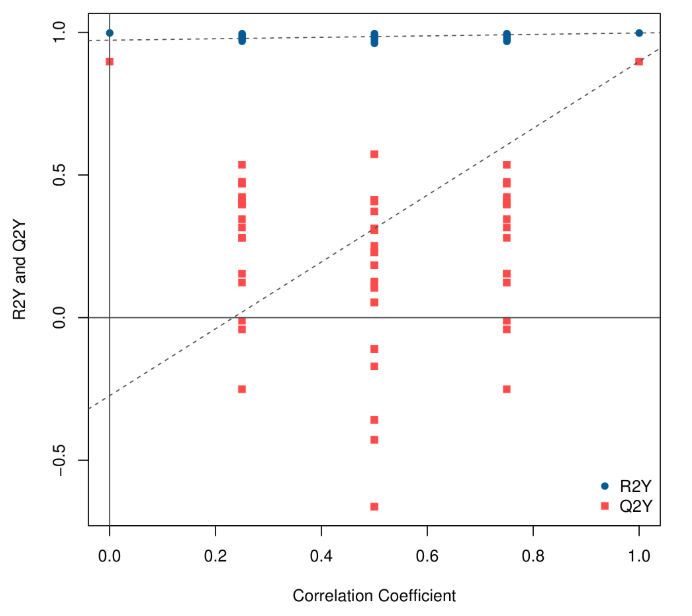


**A**

**Bcv**

**Ccv**

**Fig. S7** Correlation evaluation of various samples in the metabolome

A. M1 difference comparison group; B. M2 difference comparison group; C. M3 difference comparison group


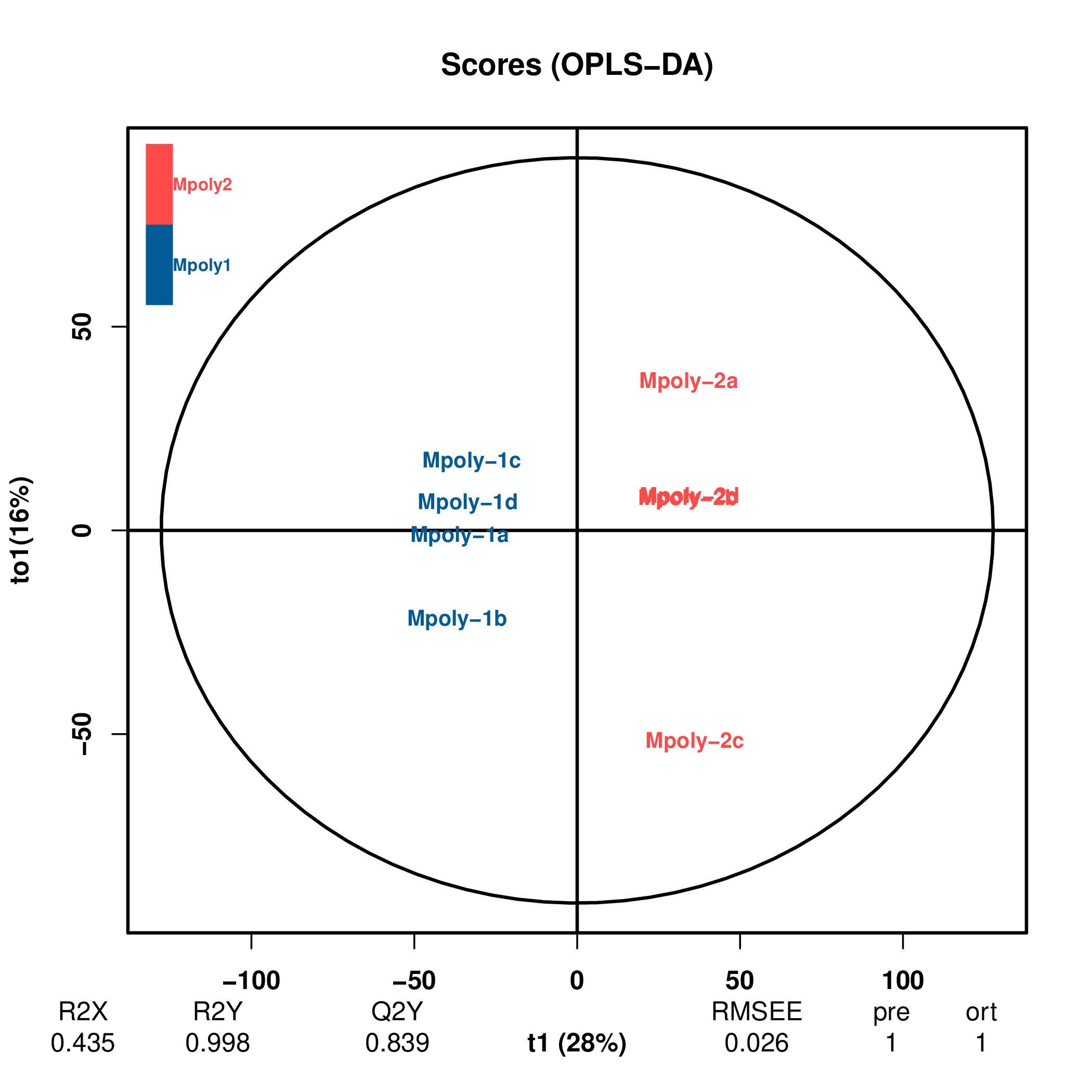


**A**


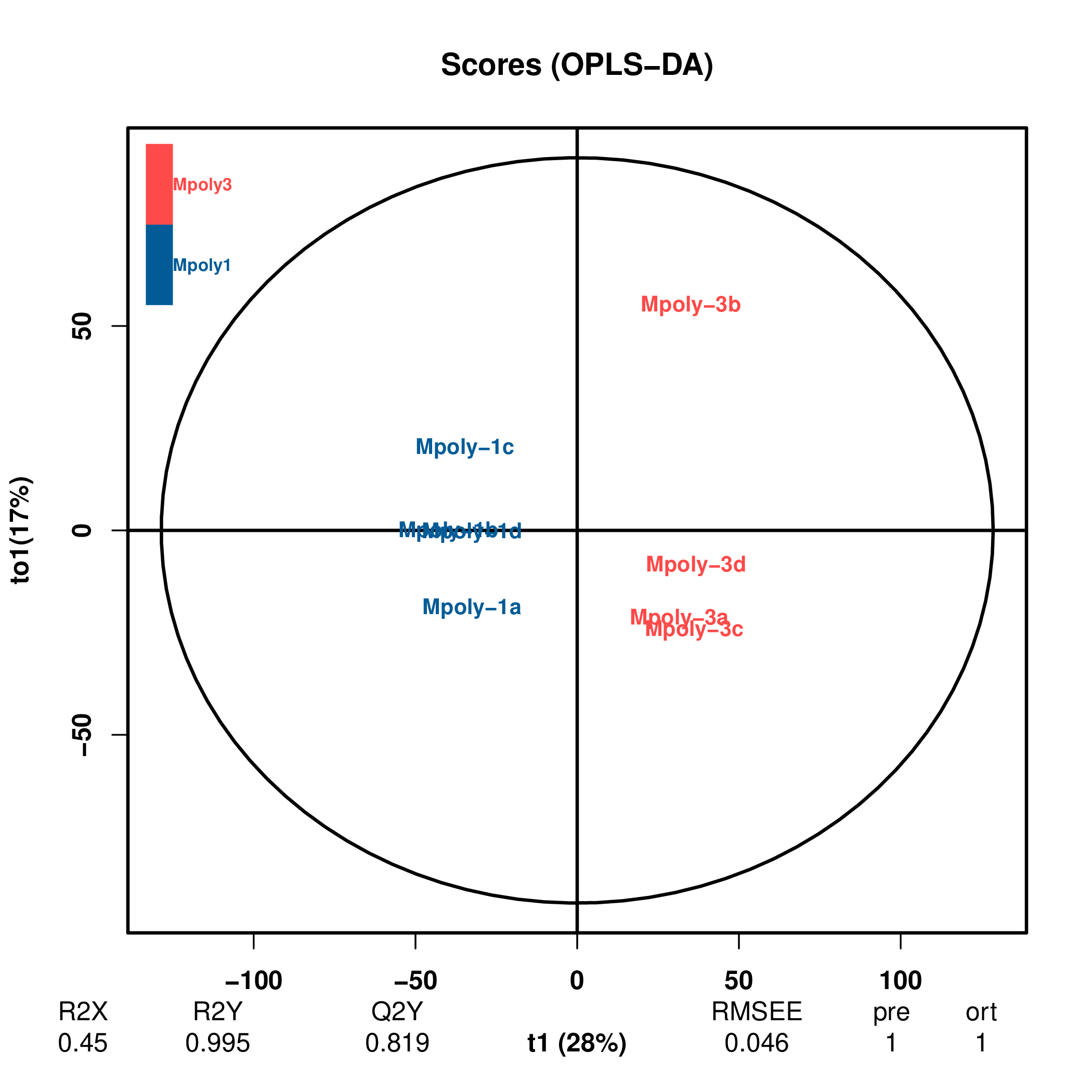


**B**


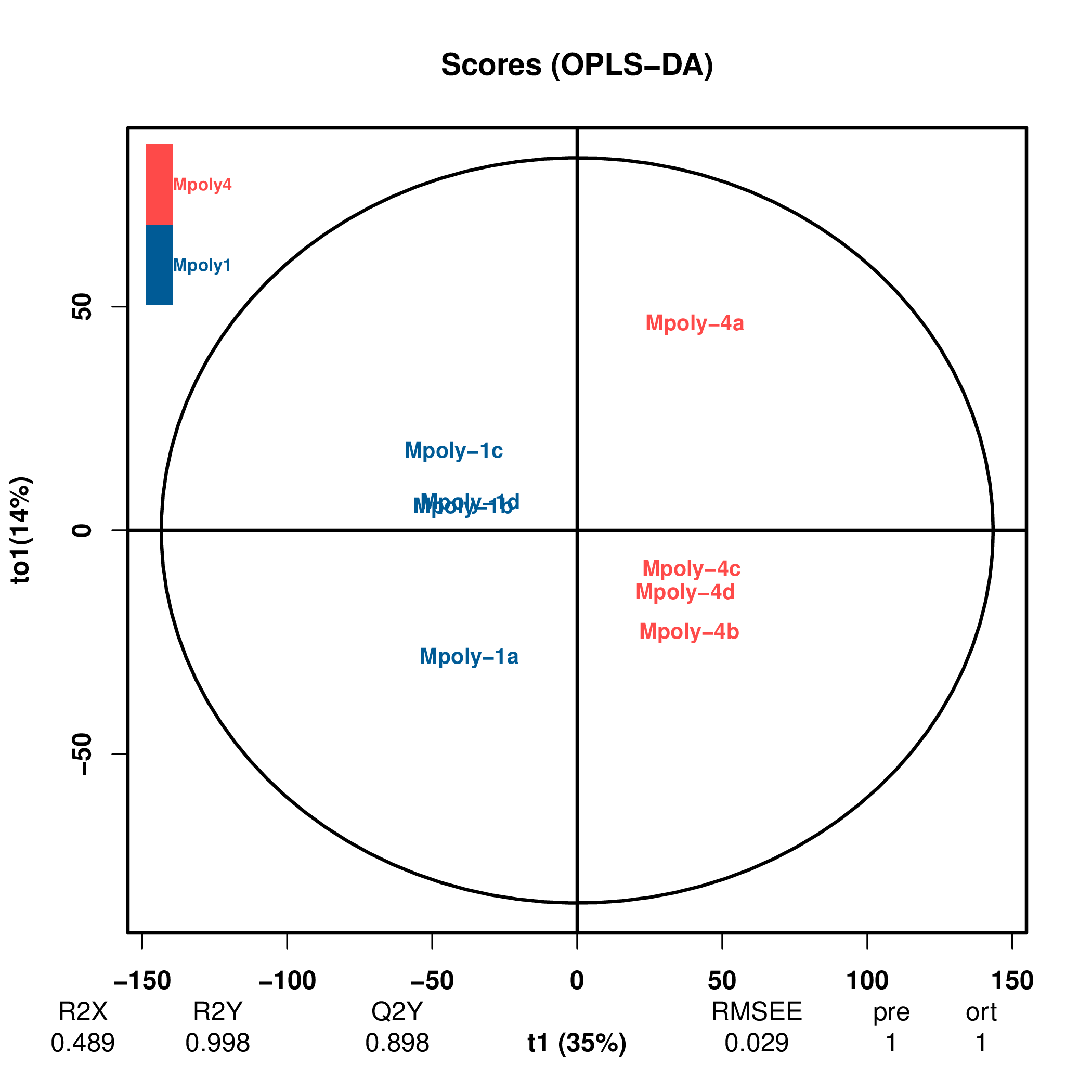


**C**

**Fig. S8** Permutation Test Plot for the OPLS-DA Model

A. M1 difference comparison group; B. M2 difference comparison group; C. M3 difference comparison group

X-axis: Correlation between permuted group labels and the original model's group labels. Y-axis: Values of *R²Y* (blue) or *Q²Y* (red). Dashed lines represent the linear regression fit for the permuted *R²Y* (blue) and *Q²Y* (red) values. A positive slope for the *Q²Y* (red) regression line indicates that the original model is statistically significant and not overfit. Blue dots (*R²Y*) generally appearing above the red dots (*Q²Y*) suggests good validity of the model and the absence of overfitting, as the predictive ability (*Q²Y*) of permuted models degrades faster than their explained variance (*R²Y*) compared to the original model.


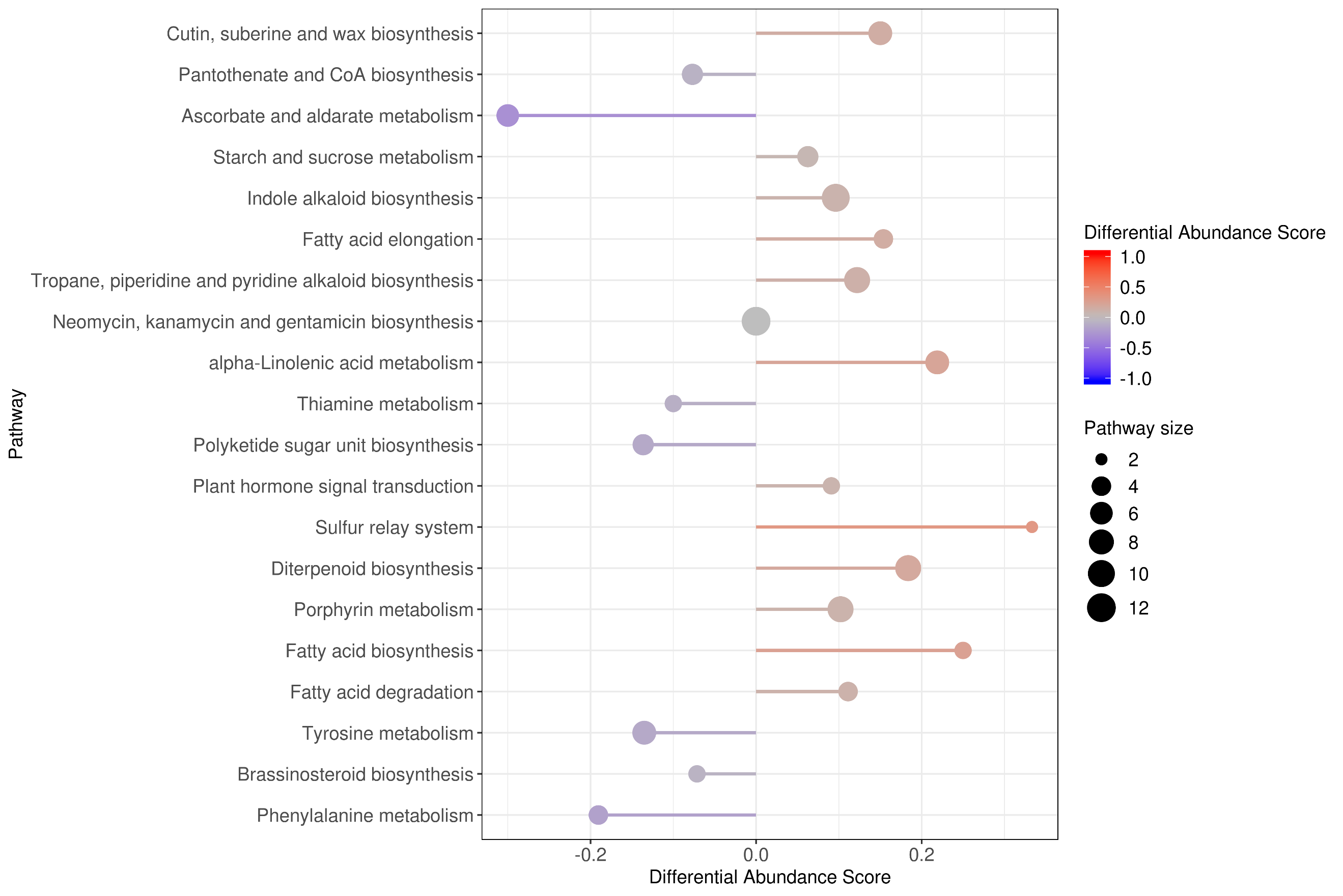

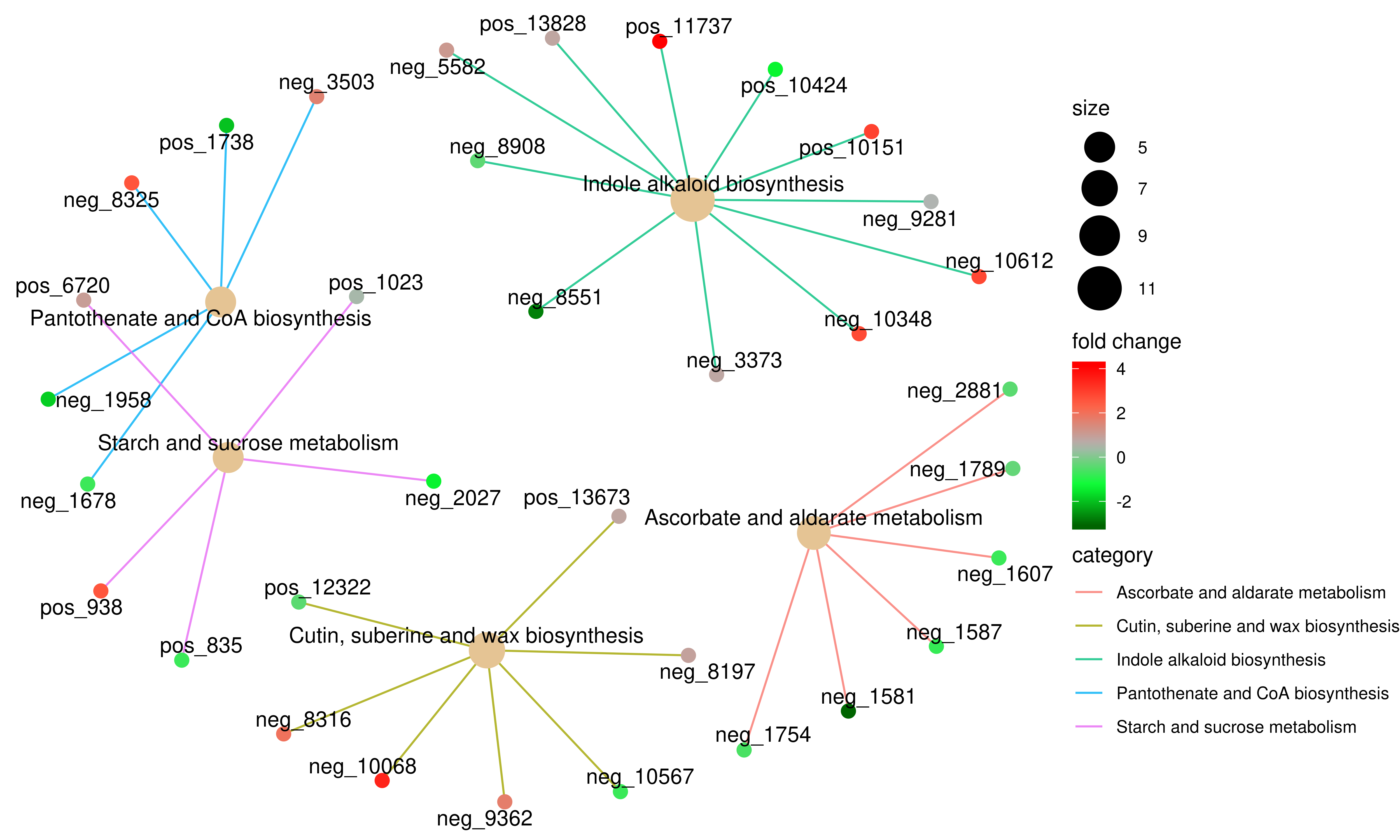


A


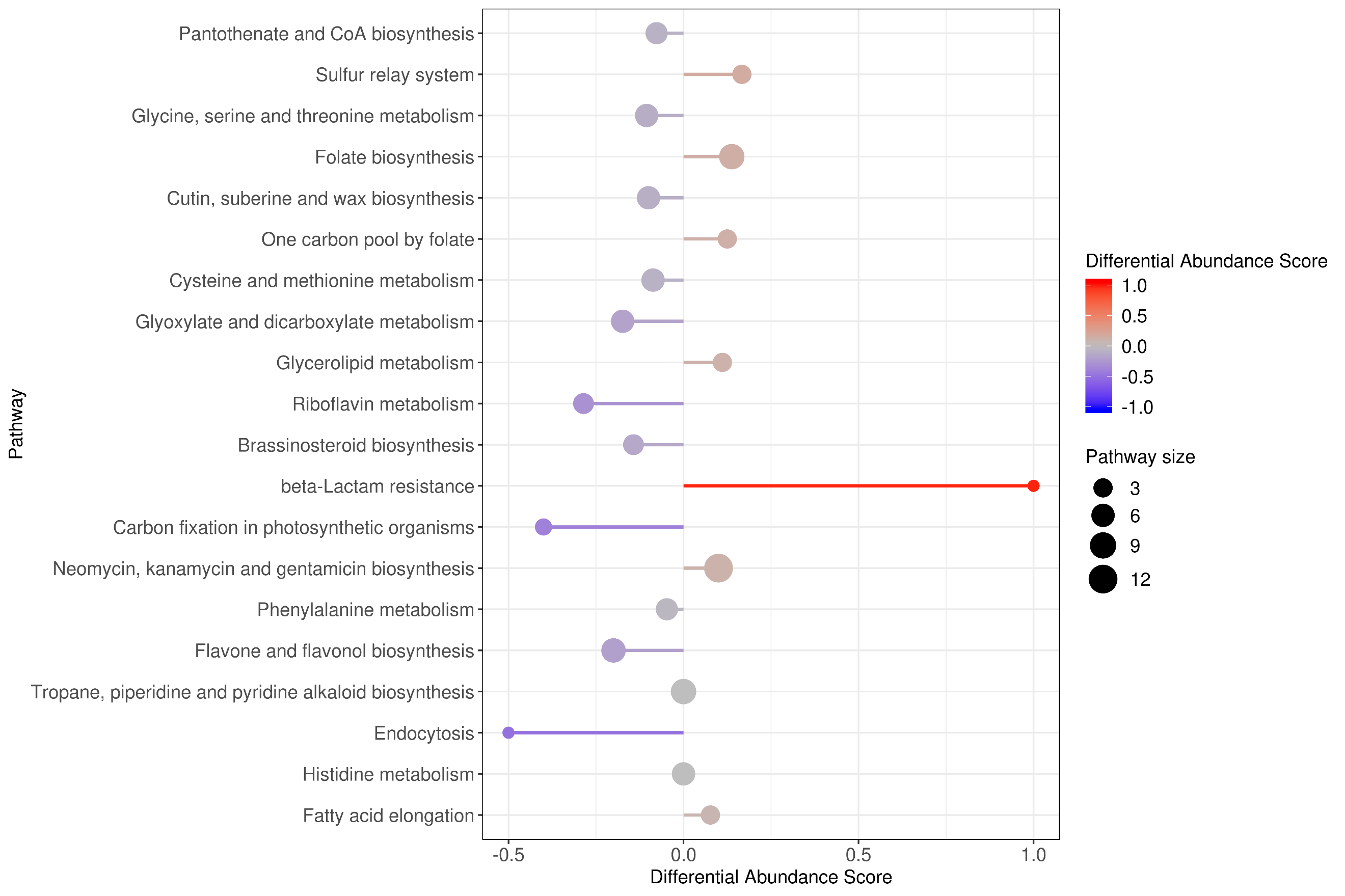

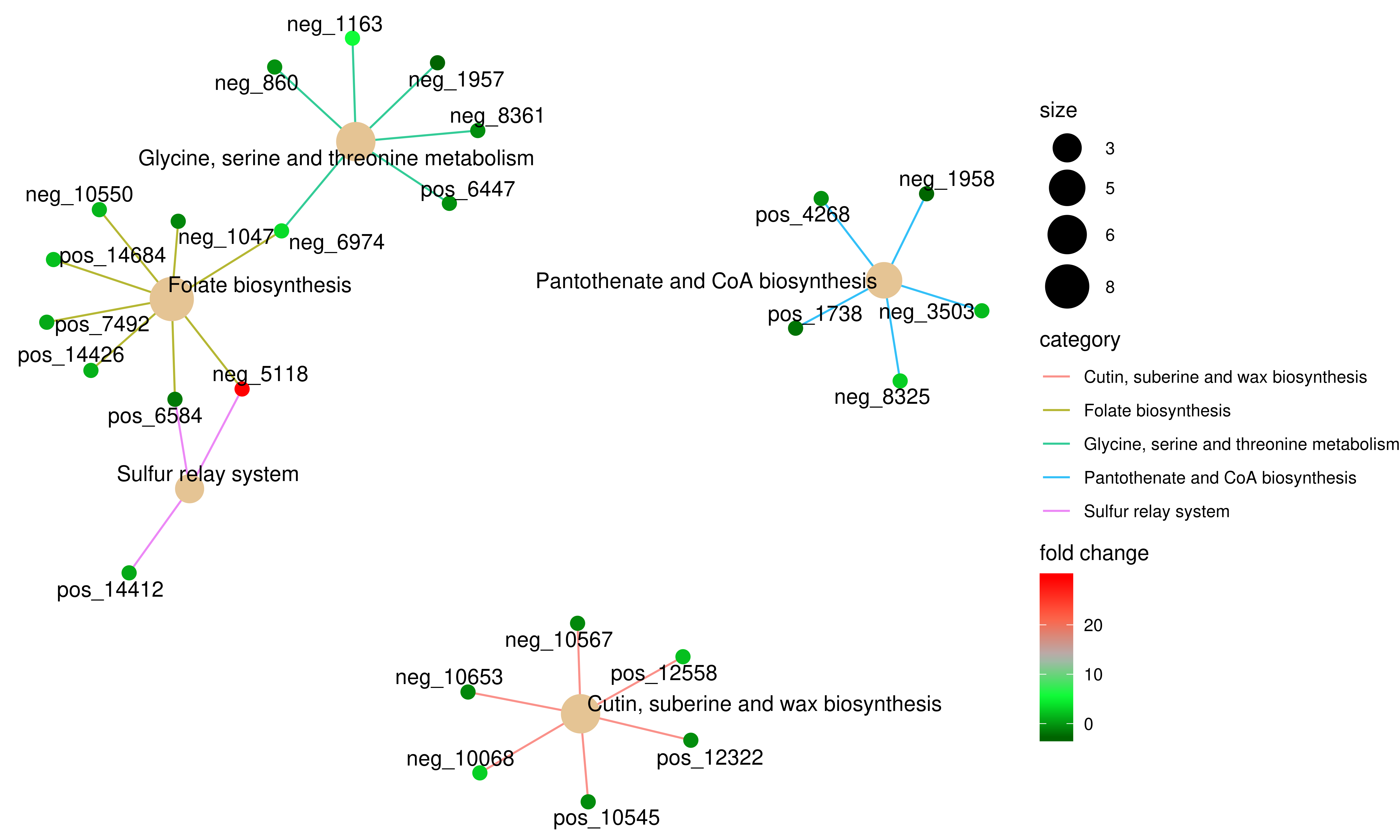


B


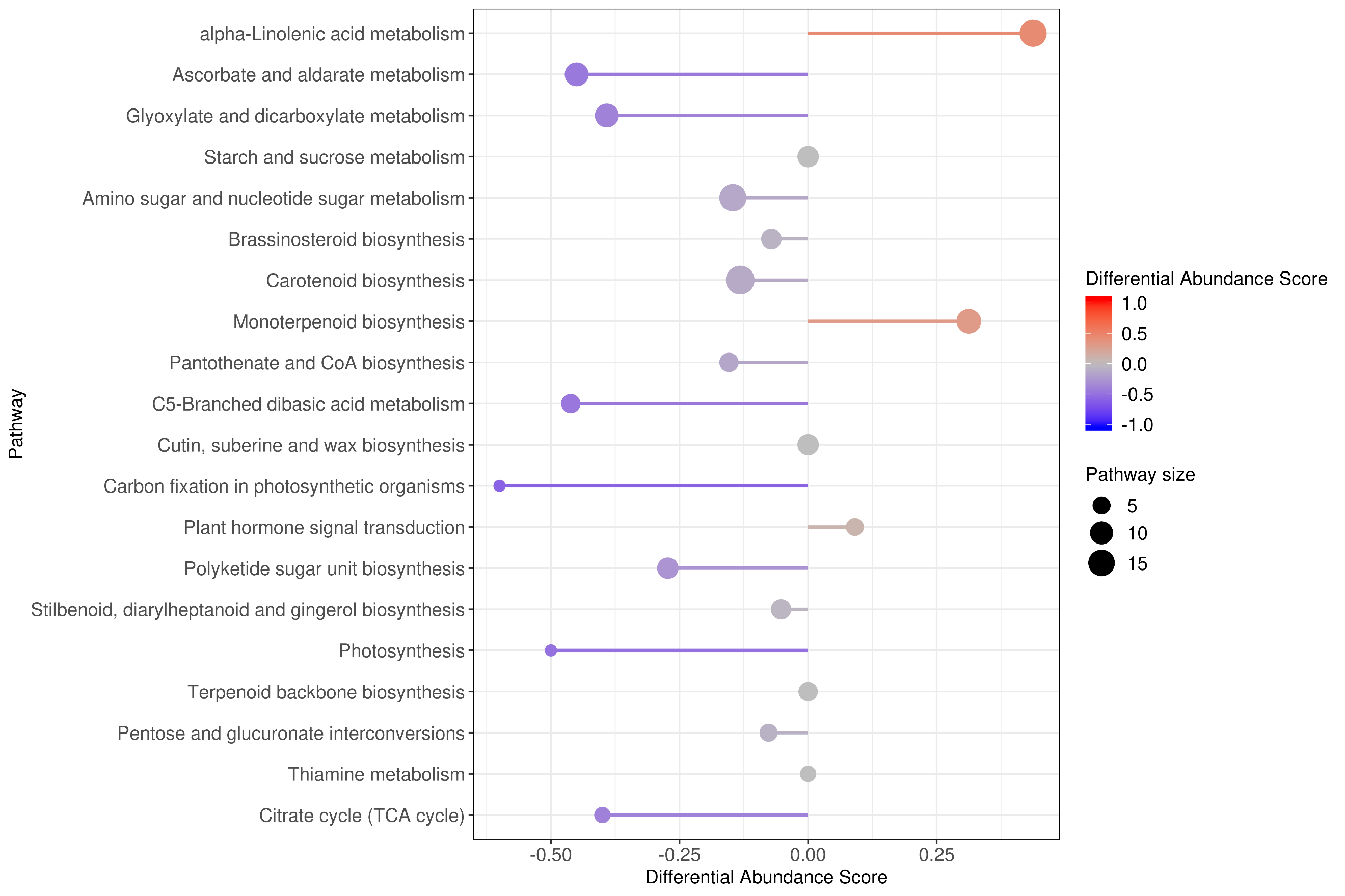

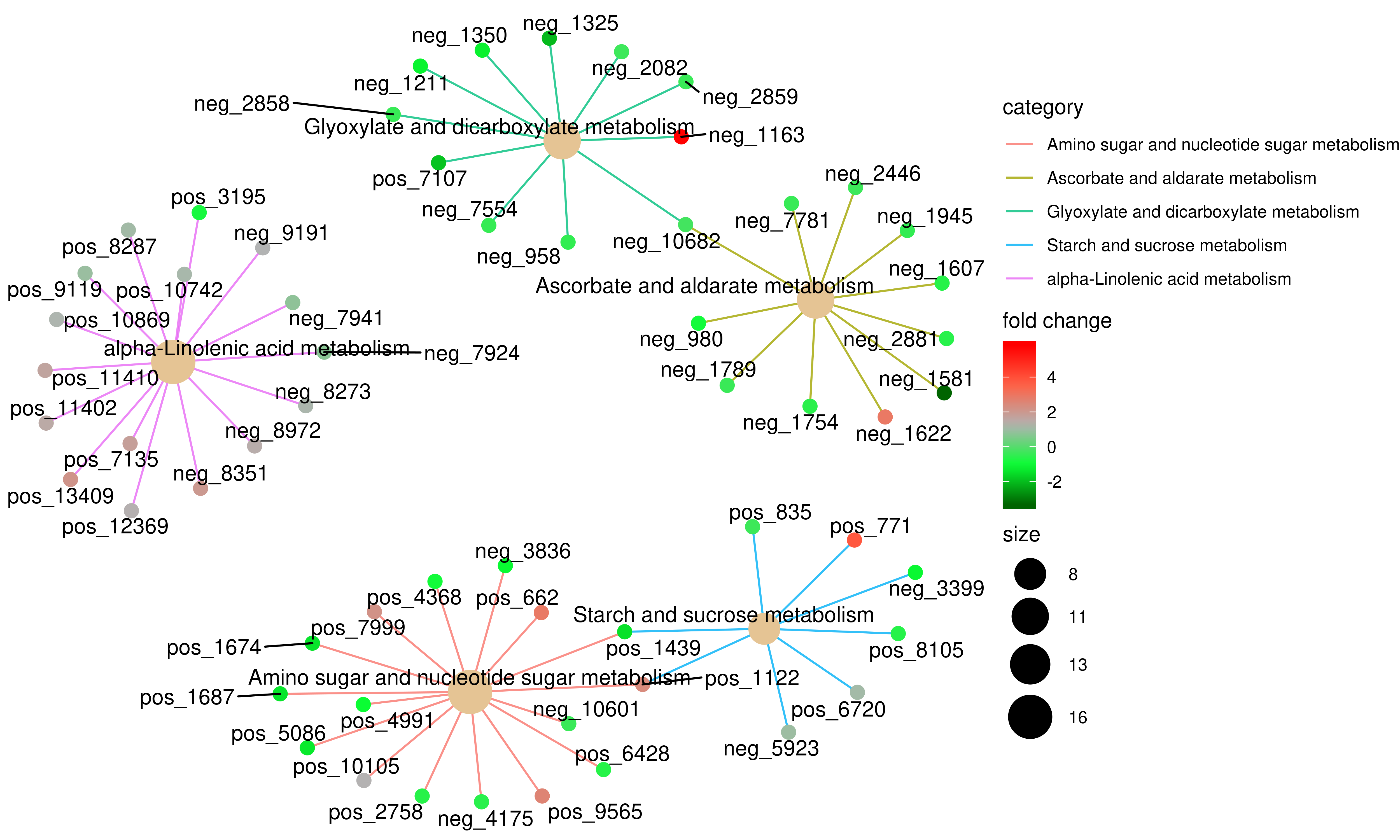


C

**Fig.S9** KEGG enrichment analysis and network chart of differential metabolite in comparative groups

A. M1 difference comparison group; B. M2 difference comparison group; C. M3 difference comparison group. The left side is the metabolic pathway annotation, the abscissa represents the metabolite abundance score, the vertical axis represents the annotated metabolic pathway, the right is the metabolite network diagram, the size of the dot indicates the number of metabolites annotated, and the line color represents different metabolic pathways


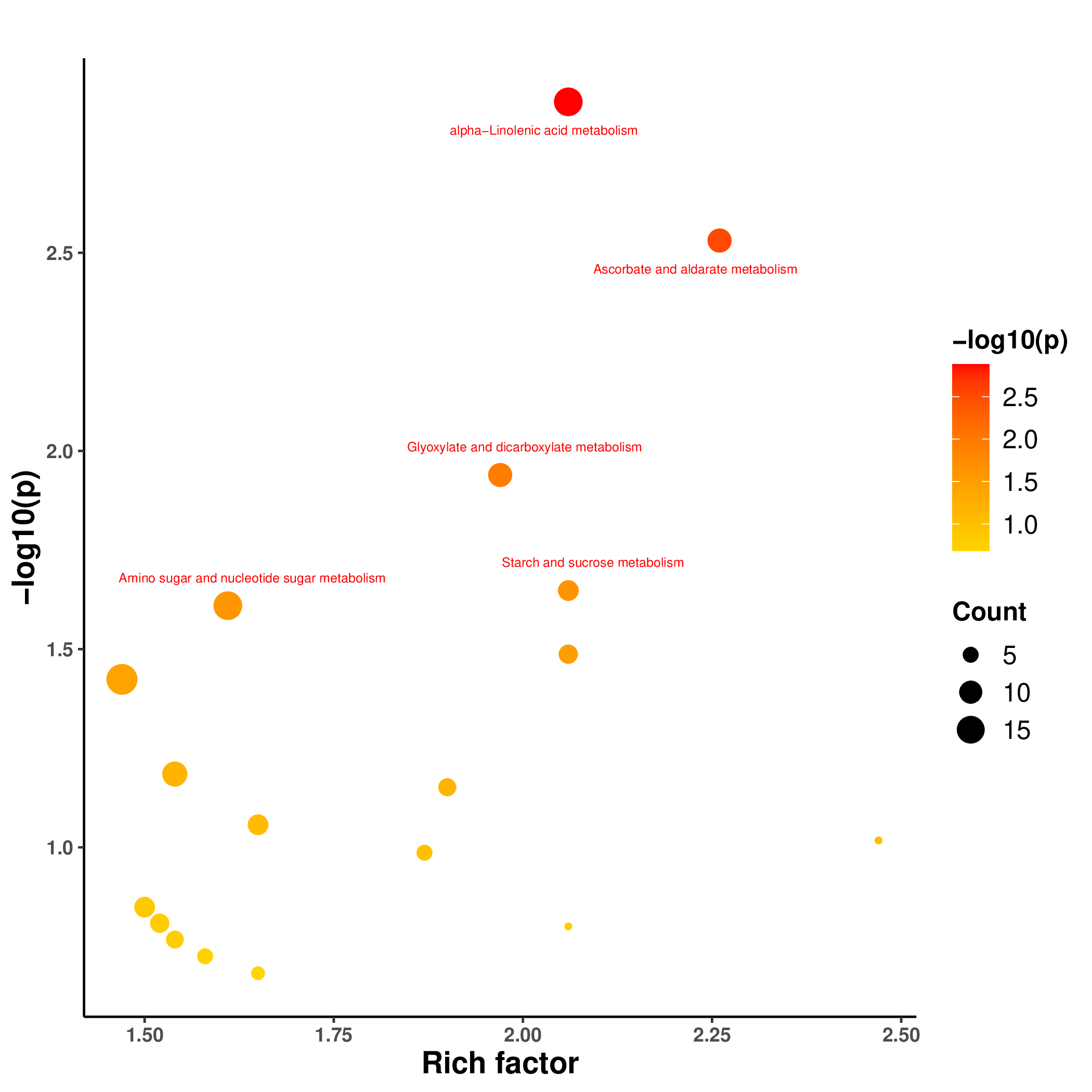

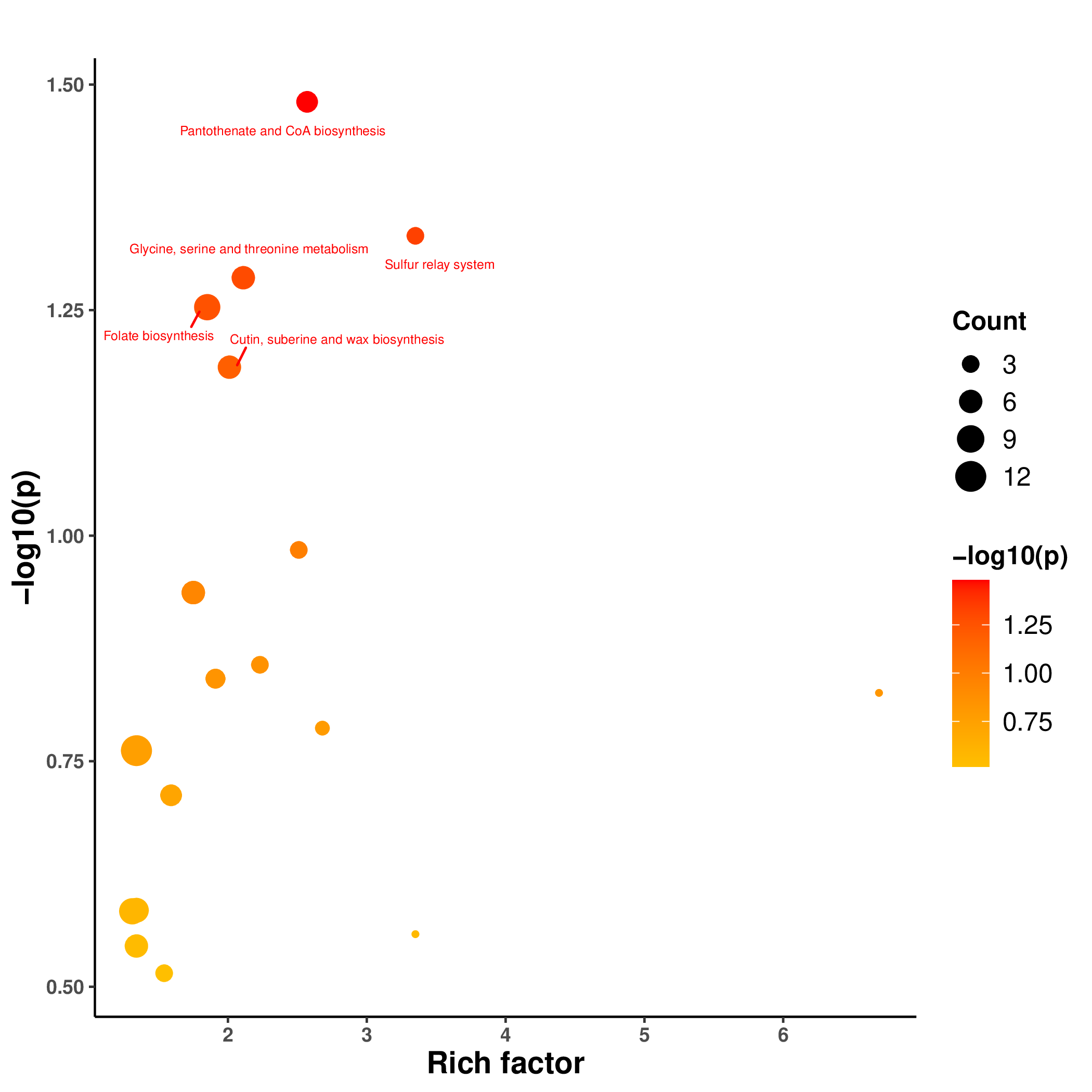

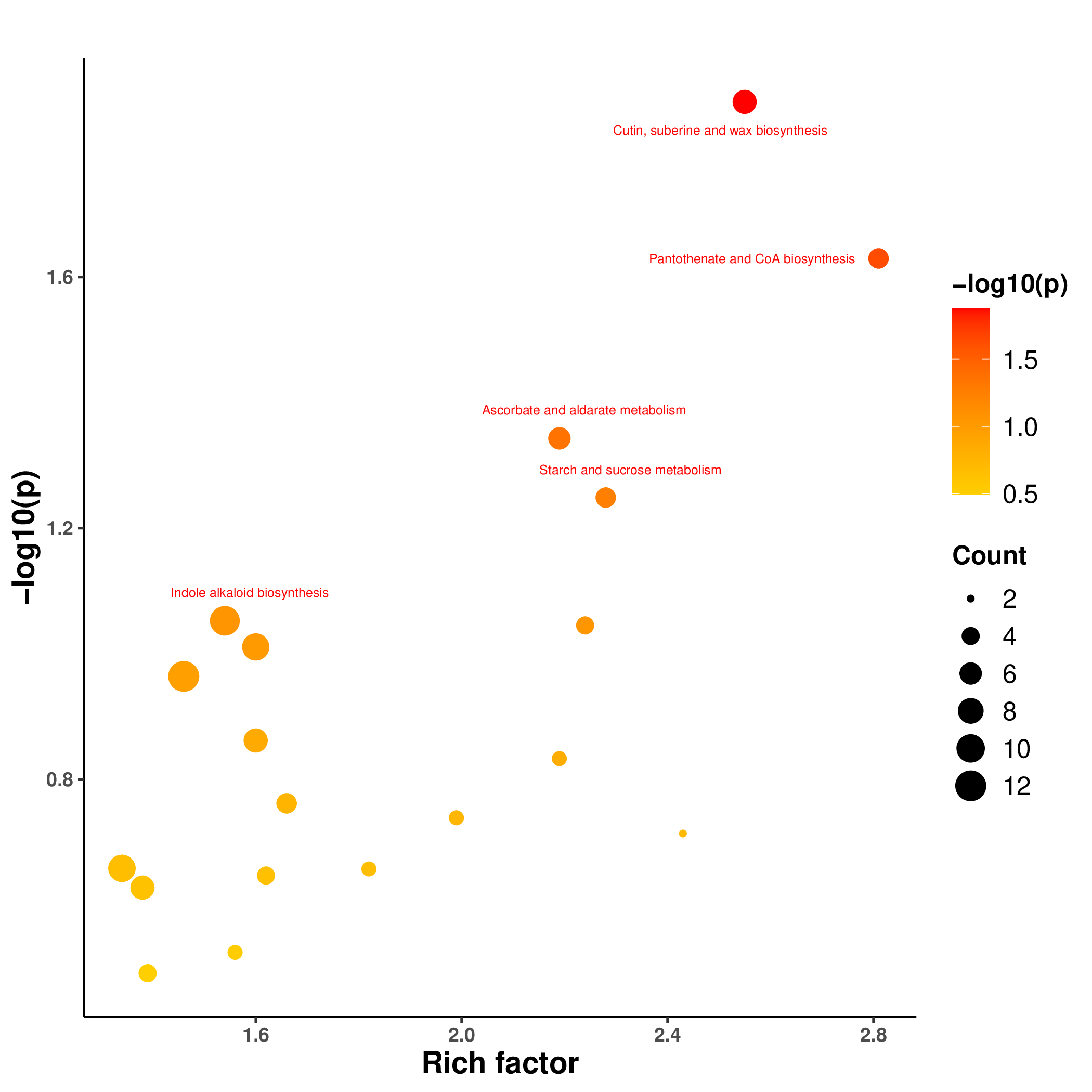


**A**

**B**

**C**

**Fig. S10** Bubble Plot of KEGG Enrichment Factor for Differential Metabolites.

A. M1 difference comparison group; B. M2 difference comparison group; C. M3 difference comparison group.

The x-axis represents the enrichment factor for the differential metabolites enriched in the corresponding pathway, the y-axis represents the p-value of the pathway enrichment, and the size of each bubble indicates the number of differential metabolites enriched in that pathway.

**Fig. S11** Differential Grouping Principal Component Analysis. Unconstrained PCoA (for principal coordinates PC1and PC2) based Bray-Curtis distances

onBray-Curtisdistances


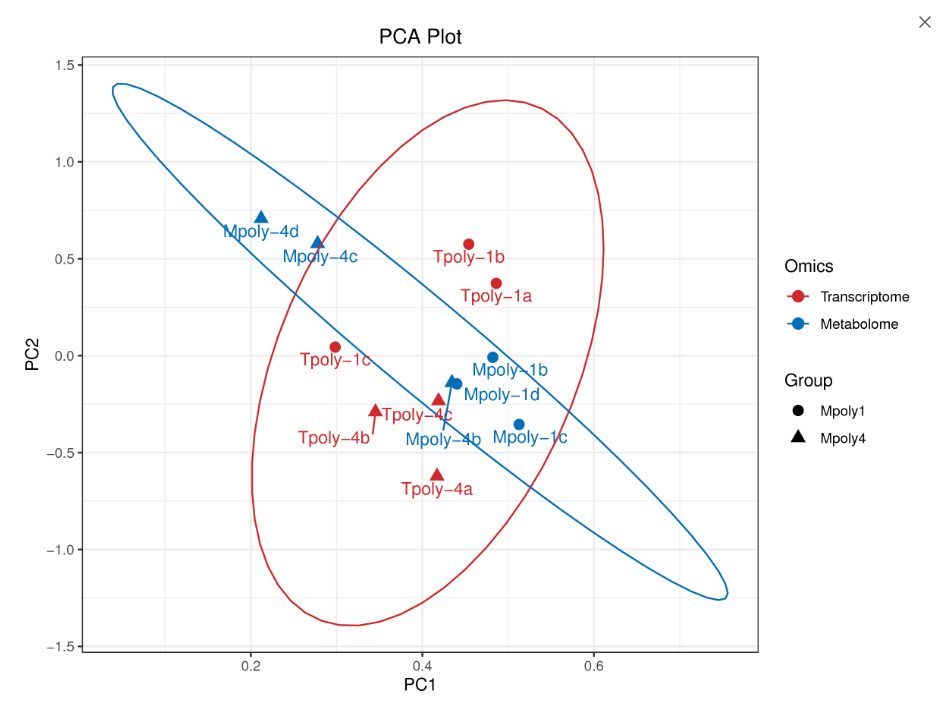

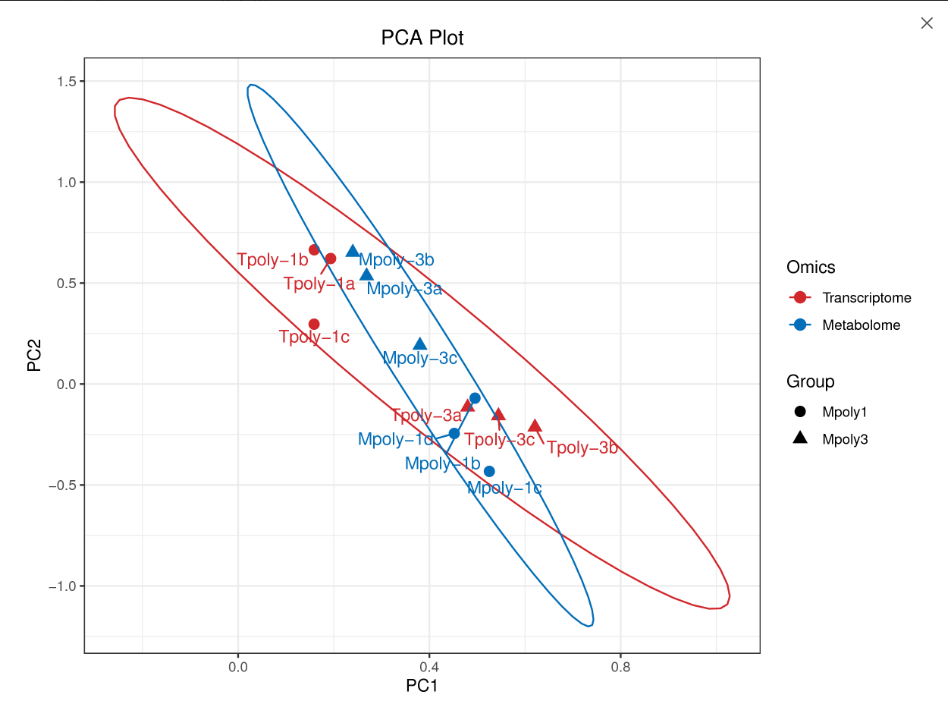

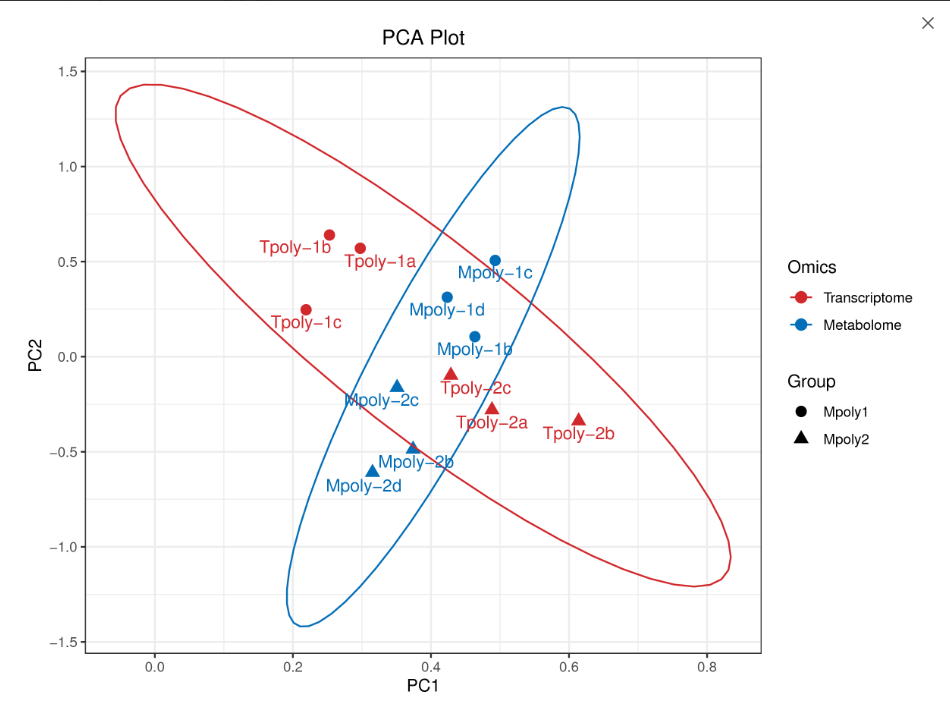

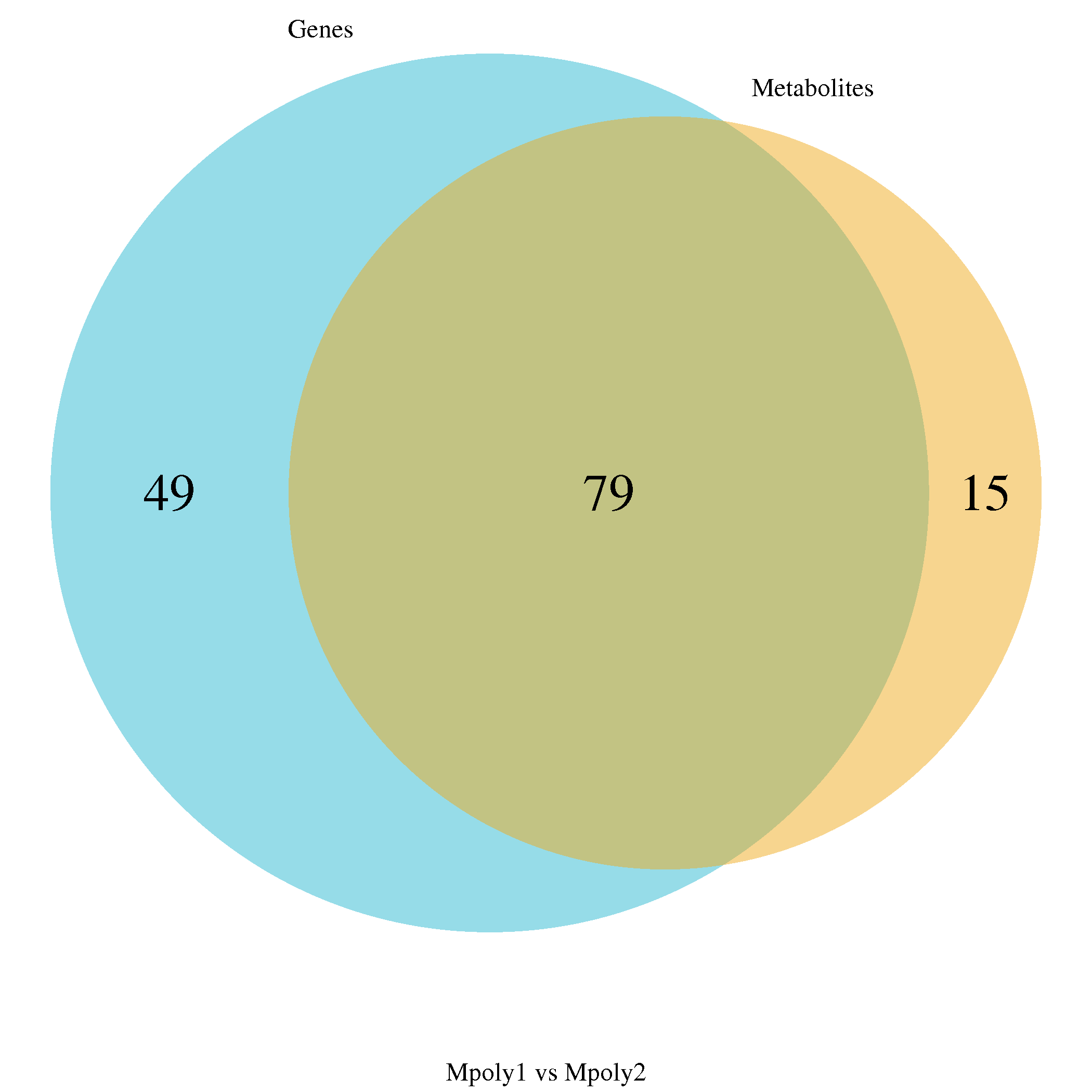

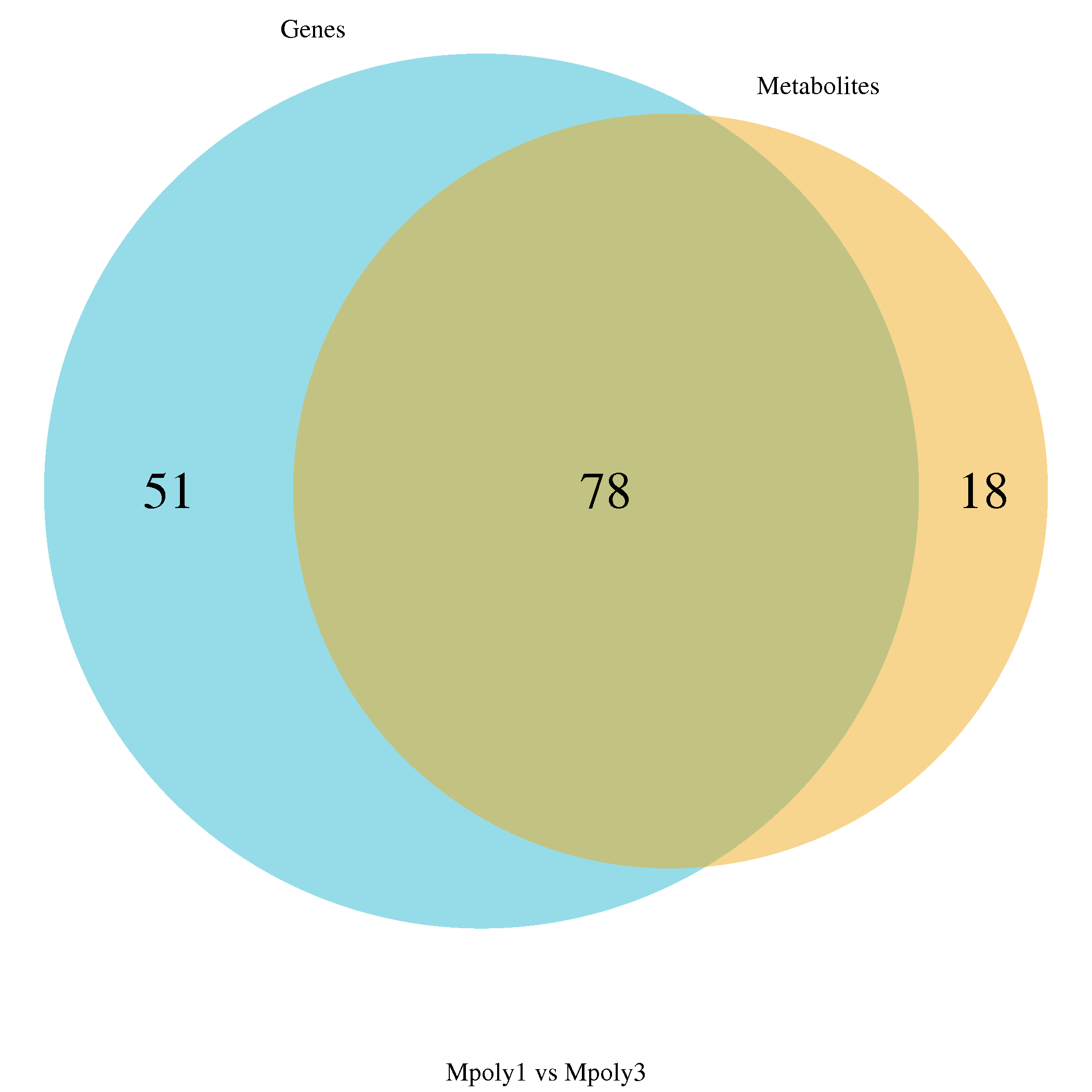

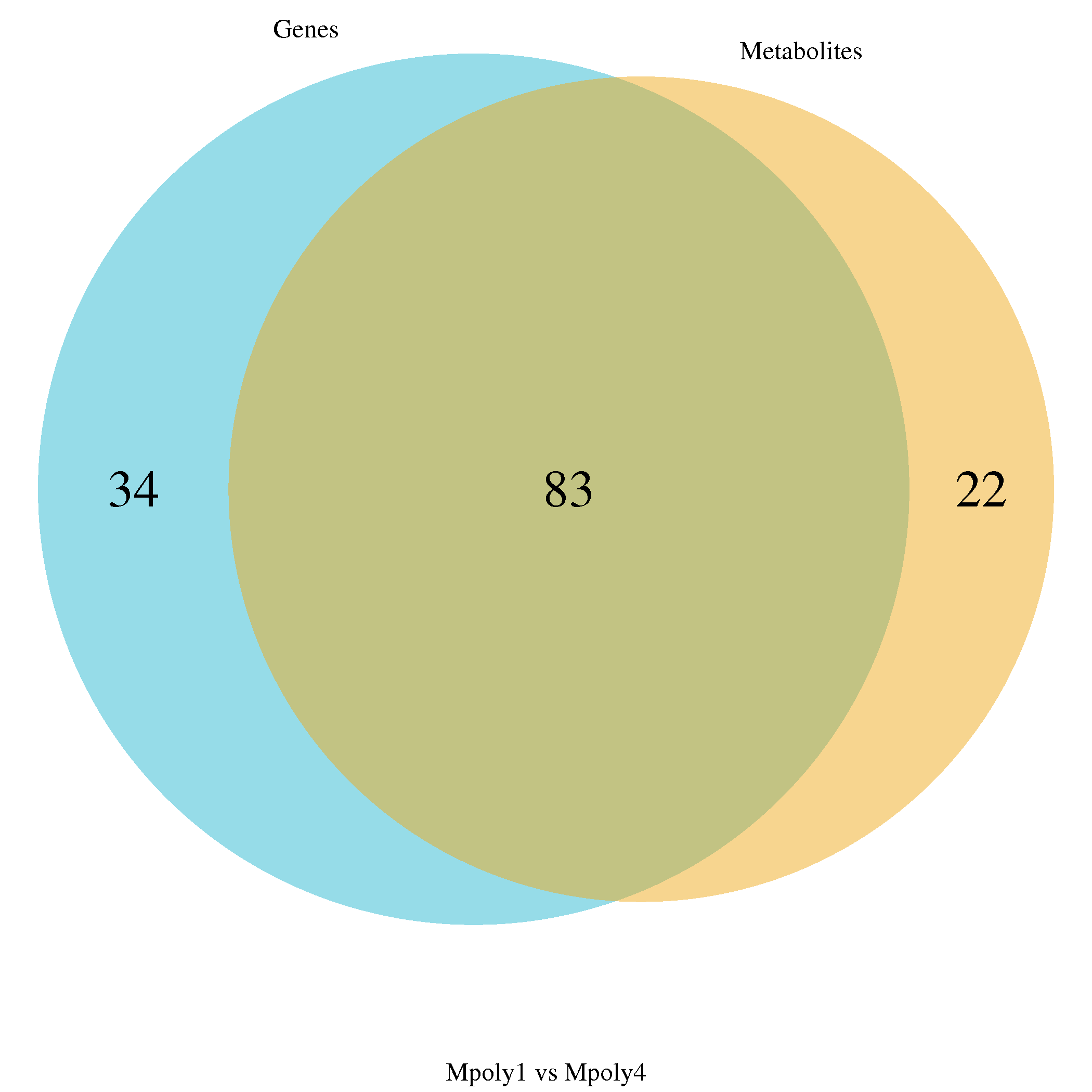


**Fig. S12** Differential genes and metabolite Venn chart


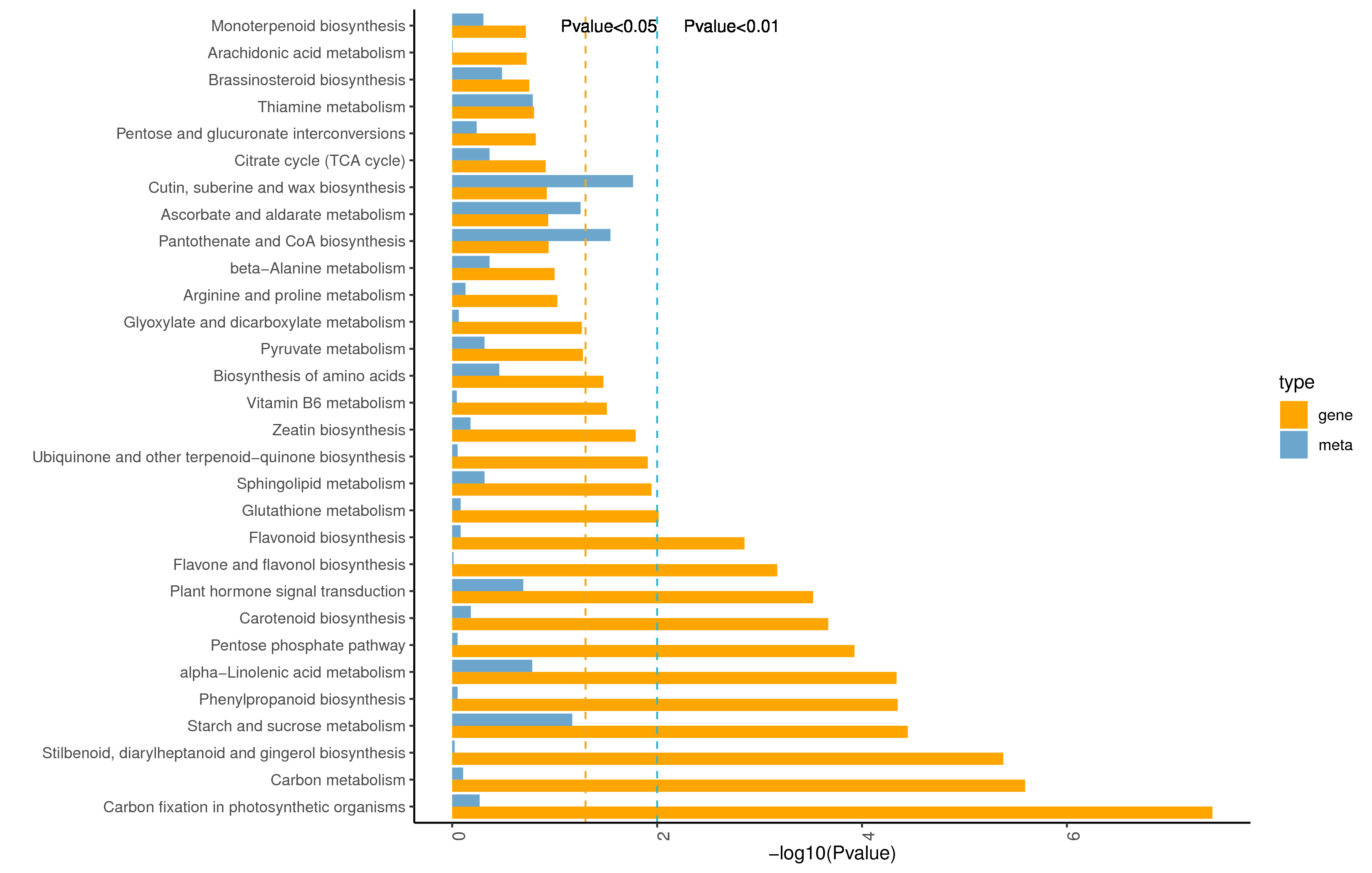

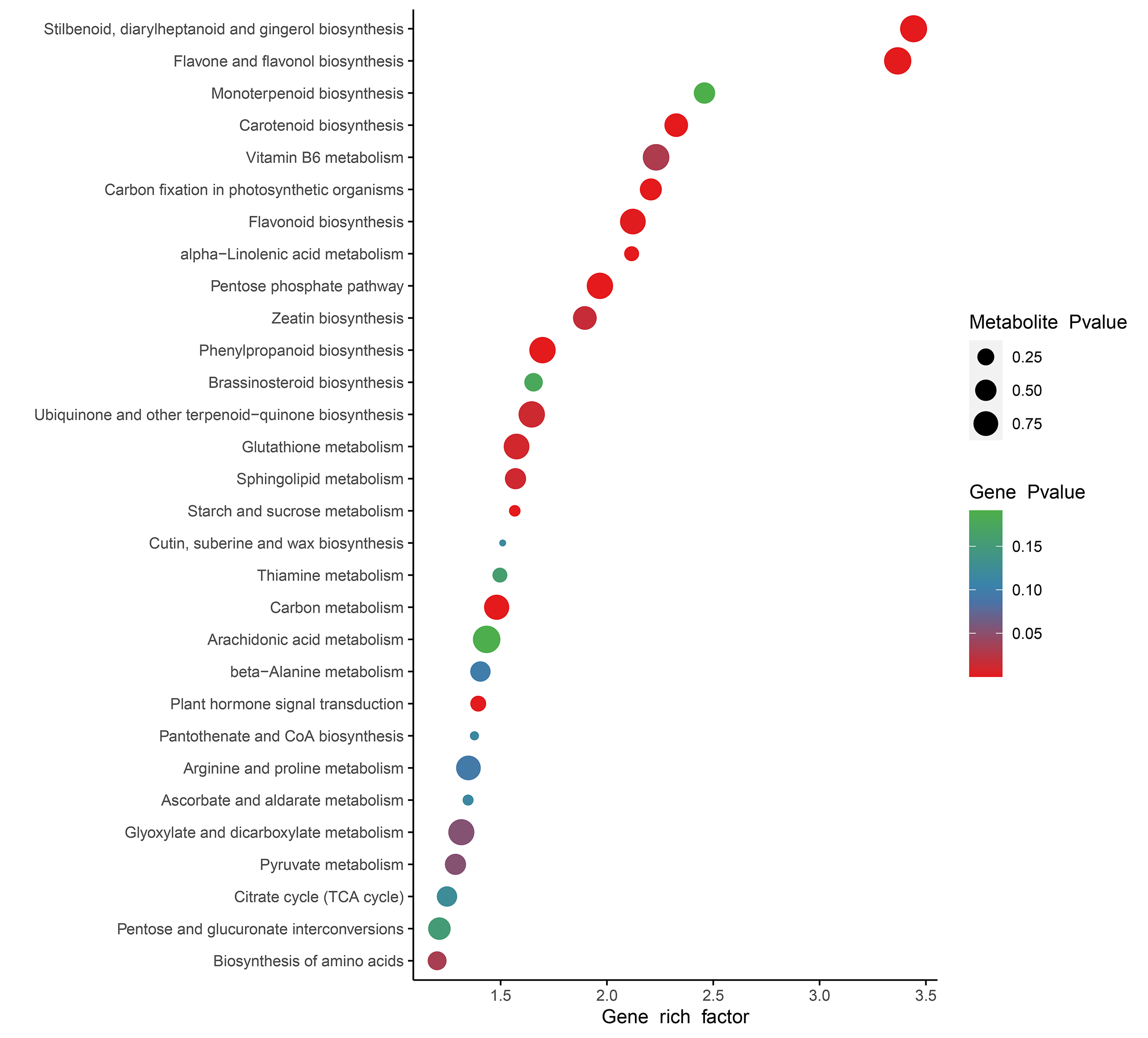


**A**


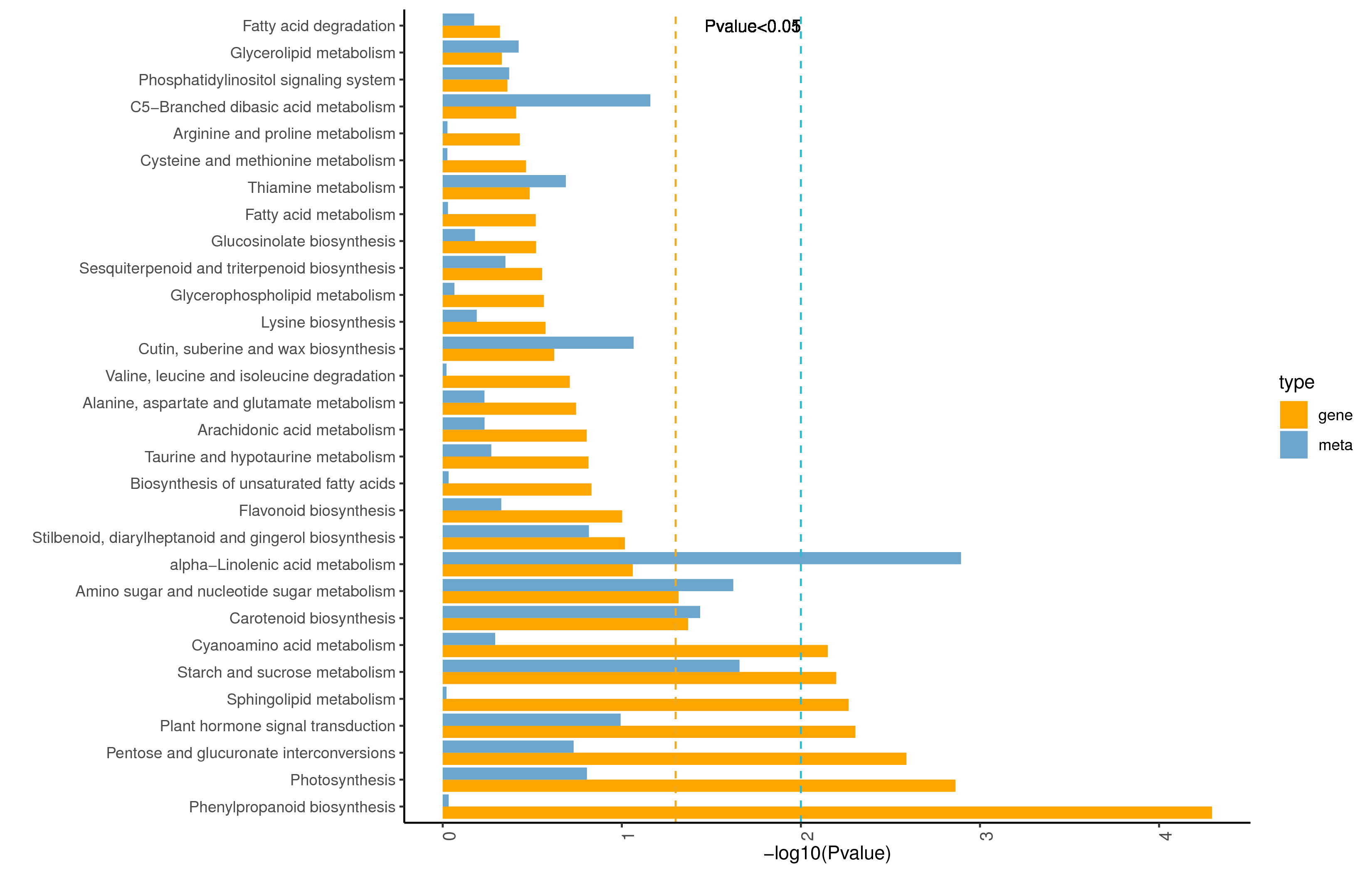

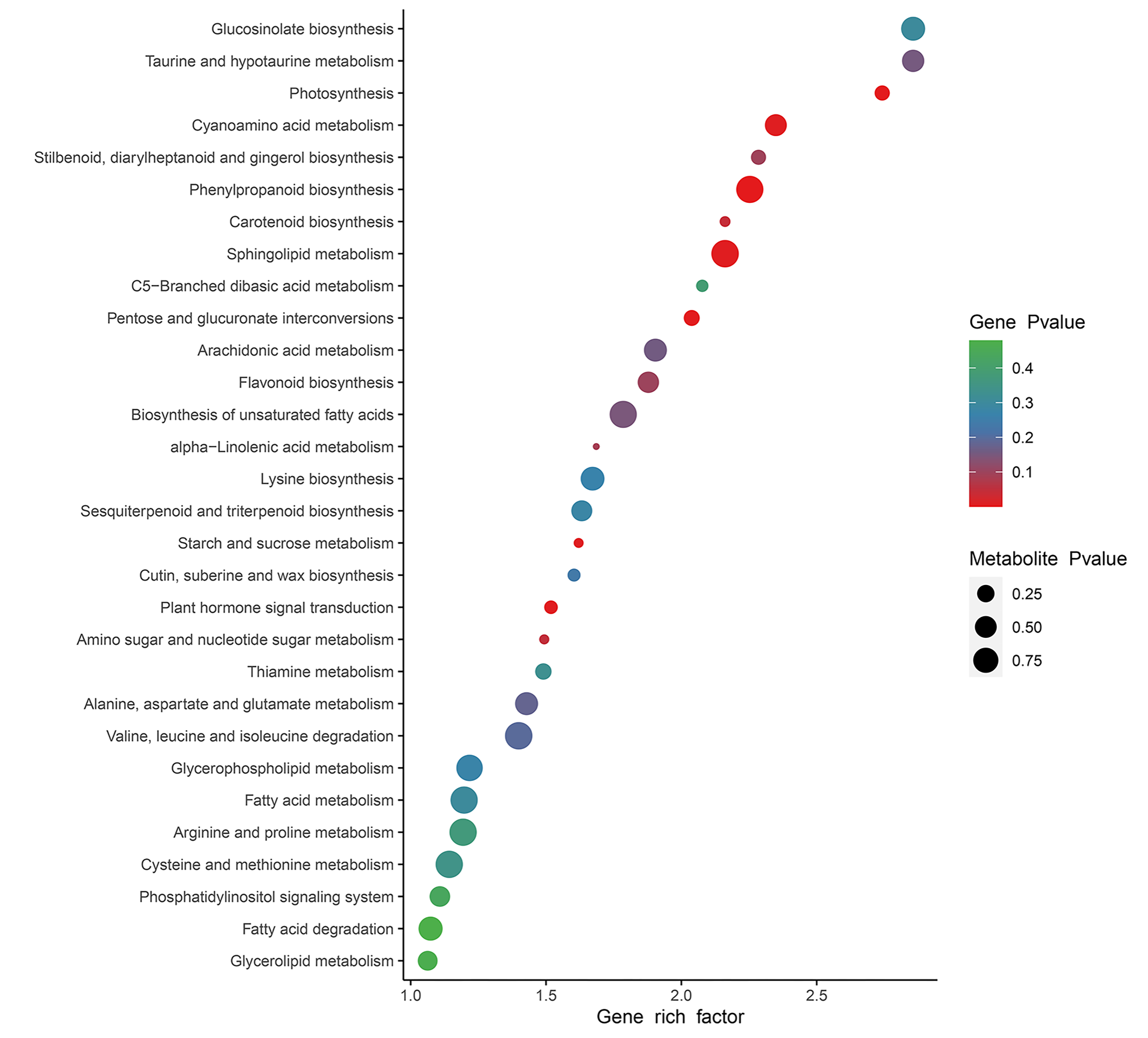


**C**


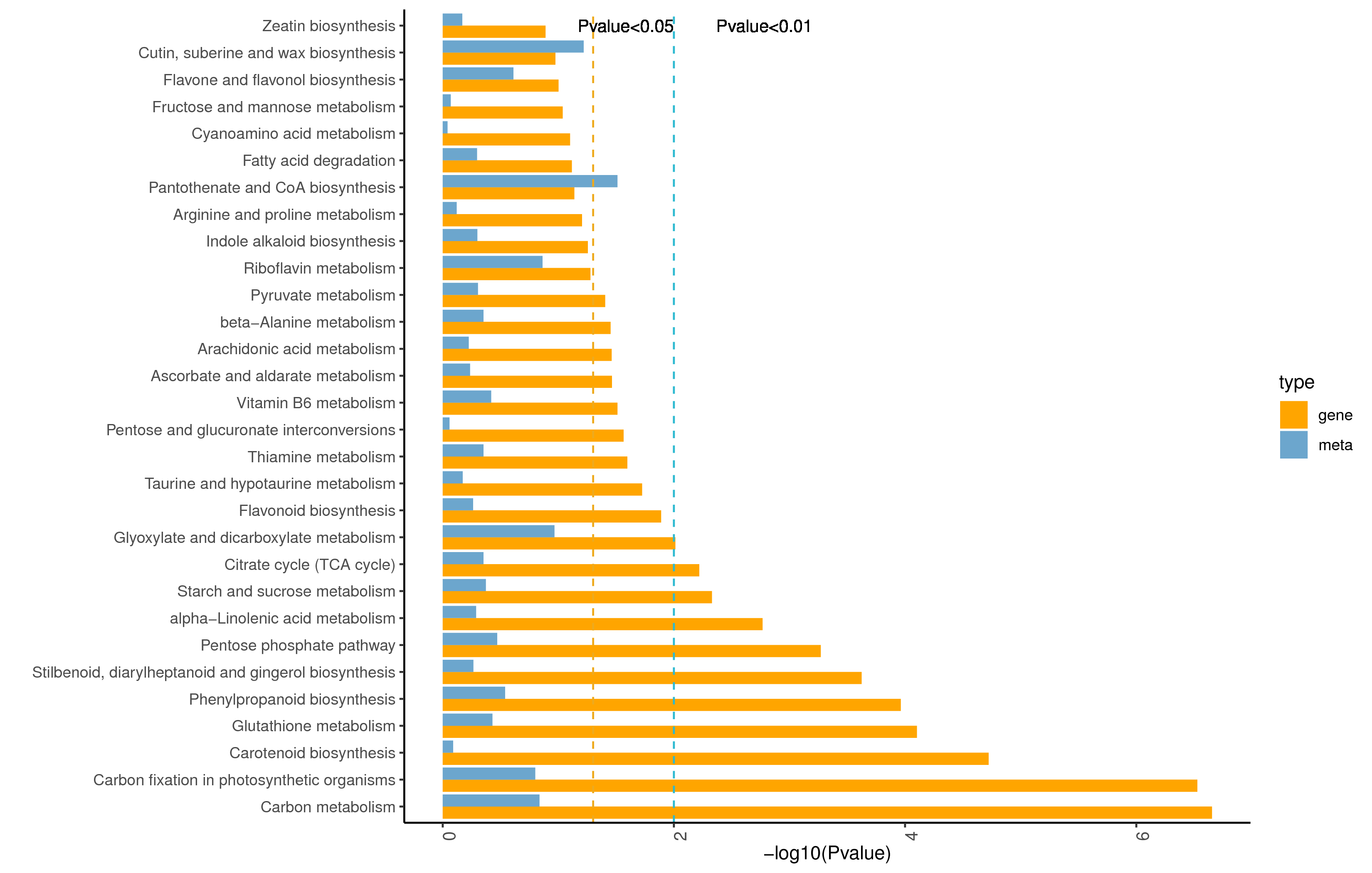

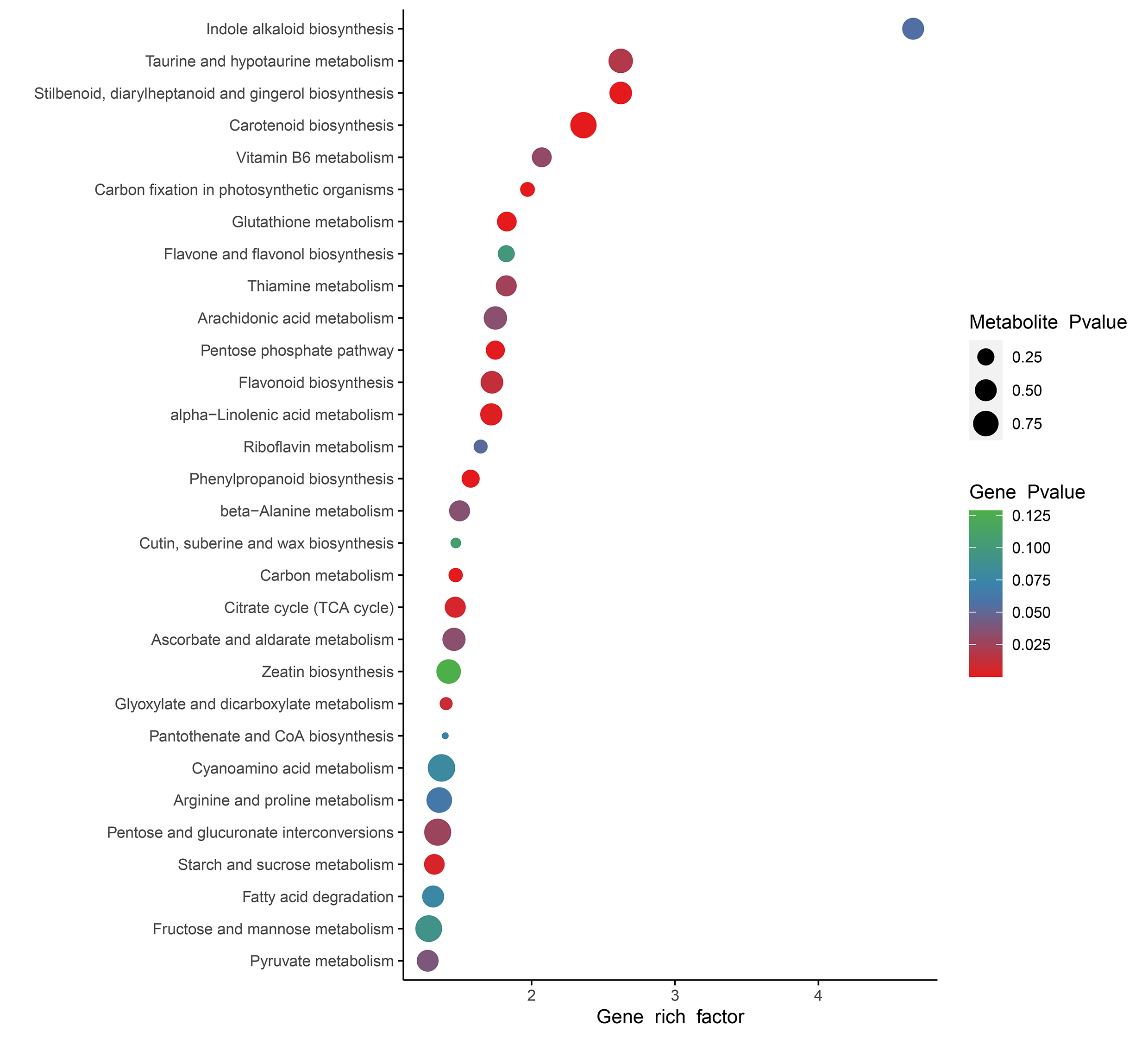


**B**

**Figure. S13** Enrichment analysis of differentially expressed genes and metabolites KEGG in the comparison group. A. G1 difference comparison group; B. G2 difference comparison group; C. G3 difference comparison group. Each column represents a KEGG pathway in the left, with different colors representing different omics. Yellow represents the transcriptome, and blue represents the metabolome; The Fig. on the right represents the enriched bubble diagram of the top 30 differential pathways in KEGG annotations
