# Supplementary material for: Combined transcriptome and metabolome analysis of Polygonatum cyrtonema Hua in response to Botrytis deweyae infection
Source: Front Plant Sci. 2025 Jul 29;16:1617308. doi: 10.3389/fpls.2025.1617308 (PMC12339532; doi:10.3389/fpls.2025.1617308)
Supplement: Supplementary file 2 [file Table1.docx]

**Table S1**. Sequencing Data Statistics Table

| sample number | Read Number | Base Number | GC Content（%） | %≥Q30 |
| --- | --- | --- | --- | --- |
| Tpoly-1a | 20,888,991 | 6,254,897,664 | 48.20% | 94.88% |
| Tpoly-1b | 21,960,219 | 6,574,041,252 | 48.34% | 94.94% |
| Tpoly-1c | 21,574,860 | 6,458,064,462 | 48.14% | 89.81% |
| Tpoly-2a | 19,778,135 | 5,921,314,070 | 48.92% | 94.79% |
| Tpoly-2b | 21,213,980 | 6,350,746,946 | 49.34% | 95.45% |
| Tpoly-2c | 22,385,546 | 6,700,469,954 | 49.10% | 90.03% |
| Tpoly-3a | 20,170,578 | 6,039,515,302 | 48.87% | 94.76% |
| Tpoly-3b | 21,341,005 | 6,389,291,564 | 48.83% | 95.11% |
| Tpoly-3c | 20,533,386 | 6,147,639,312 | 48.99% | 95.05% |
| Tpoly-4a | 19,270,586 | 5,770,536,192 | 48.88% | 94.52% |
| Tpoly-4b | 21,921,708 | 6,561,661,636 | 49.07% | 90.43% |
| Tpoly-4c | 21,498,582 | 6,436,270,622 | 47.79% | 93.48% |

**Table S2.** Repetitive groups and grouping of each sample

| Sampling time point | | Transcriptome samples | Metabolome samples |
| --- | --- | --- | --- |
| 0 h | Tpoly1a; Tpoly1b; Tpoly1c | | Mpoly1a; Mpoly1b; M poly1c; Mpoly1d |
| 24 h | Tpoly2a; Tpoly2b; Tpoly2c | | Mpoly2a; Mpoly2b; M poly2c; Mpoly2d |
| 48 h | Tpoly3a; Tpoly3b; Tpoly3c | | Mpoly3a; Mpoly3b; M poly3c; Mpoly3d |
| 96 h | Tpoly4a; Tpoly4b; Tpoly4c | | Mpoly4a; Mpoly4b; M poly4c; Mpoly4d |

The samples were collected at 24 h, 48 h, and 96 h post-inoculation as the treatment groups, while the 0 h time point served as the control group.

**Table S3.** Information on the comparison of transcriptome between groups

| Define groups | Comparison groups | Control vs. Treatment samples |
| --- | --- | --- |
| G1 | Tpoly1 (0 h) vs Tpoly2 (24 h) | Tpoly1a; Tpoly1b; Tpoly1c vs Tpoly2a; Tpoly2b; Tpoly2c |
| G2 | Tpoly1 (0 h) vs Tpoly3 (48 h) | Tpoly1a; Tpoly1b; Tpoly1c vs Tpoly3a; Tpoly3b; Tpoly3c |
| G3 | Tpoly1 (0 h) vs Tpoly4 (96 h) | Tpoly1a; Tpoly1b; Tpoly1c vs Tpoly4a; Tpoly4b; Tpoly4c |

The samples were collected at 24 h, 48 h, and 96 h post-inoculation as the treatment groups, while the 0 h time point served as the control group.

**Table S4.** Primer Sequences for qRT-PCR Validation

| Gene | KEGG Orthology accession（KO） | KEGG pathway | Primer sequences 5'—3' | Base numbers |
| --- | --- | --- | --- | --- |
| UBQ-E2F | \ | \ | GGA CCC AGA AGT ACG CAA TG | 20 |
| UBQ-E2R | \ | \ | AAT TAC CAG GGA TAC AGC ACC | 21 |
| EF-1α2F | \ | \ | CCC TTC TTG ACG CTC TTG AC | 20 |
| EF-1α2R | \ | \ | GAG CTT CAT GGT GCA TCT CA | 20 |
| Tp1198F | ko04626 | Plant-pathogen interaction | GGT AGC ATC AAC AAG AGC CTT C | 22 |
| Tp1198R |  |  | GCA ATC CTG GAA CTC GTC ACT | 21 |
| TP1401F | ko00600 | Sphingolipid metabolism | CCC TCC CTT CCT CTT CTC AAT CG | 23 |
| TP1401R |  |  | TTC TTC ACC TTC GCC ACC ATC AA | 23 |
| Tp1051F | ko00270 | Cysteine and methionine metabolism | GCA TCA CGC TCT TGT ACT TG | 20 |
| Tp1051R |  |  | CGG CAT CAT CCT CCT CTT C | 19 |
| Tp648F | K08238 | Unknow | GTC GCT GTC CAT CCA CCA CAA | 21 |
| Tp648R |  |  | ACC ACT ACC TGC TCA AGT CCA TC | 23 |
| Tp883F | ko01212 | Fatty acid metabolism | ATG GCA CGG CTG GAA CTG AG | 20 |
| Tp883R |  |  | GCT TTC CTG CTG TGG GTA GAG A | 22 |
| Tp1979F | Unknow | Unknow | CCA GCG GCA GGA TTC ATC AGT | 21 |
| Tp1979R |  |  | CAG CAG CAG CAG TAG TCA CAG T | 22 |
| Tp96630F | ko04626 | Plant-pathogen interaction | GCC ACA ATC CAA CTC ACC AAT GC | 23 |
| Tp96630R |  |  | TCT CCG ATA CCC AAG CGT TTA CG | 23 |
| Tp2756F | ko04070 | Phosphatidylinositol signaling system | CCA GAA CGC CTC CTC CAT TAT CC | 23 |
| Tp2756R |  |  | CCT CAA GTC CCT CTA CTC CTC CT | 23 |
| TP13372F | ko00940 | Phenylpropanoid biosynthesis | ACA AGG AAG ACA GTG GTG AAG GA | 23 |
| TP13372R |  |  | AGG CAG GAT CTA CAA GGG ACA AA | 23 |
| TP65F | ko04016 | MAPK signaling pathway - plant | GCA TTT ACA GCA GCA CAG ACG AT | 23 |
| TP65R |  |  | CAA CAT CTA CGG CAA CCT CTT CG | 23 |
| TP7014F | ko04626 | Plant-pathogen interaction | GCT GCT GAT GGA AGC ACT GGA G | 22 |
| TP7014R |  |  | AAC ACG CAC ACC TGA CAA TGG C | 22 |
| TP14157F | ko00940 | Phenylpropanoid biosynthesis | TCA CCA CCA CTA CCA CCA CCAT | 21 |
| TP14157R |  |  | TGT CCG CAG CTC CTT CCA TTG | 21 |

The ubiquitin-conjugating enzyme-E2-10 (UBQ-E2-10) and elongation factor 1-alpha isoform 2 (EF-1α2) were used as reference genes, while the remaining genes were considered as target genes. Unknow means that the KEGG annotation library is not annotated to pathways or genes.

**Table S5. Information on the comparison of metabolomics between groups**

| Define groups | Comparison groups | | Control vs. Treatment samples |
| --- | --- | --- | --- |
| M1 | | Mpoly1 (0 h) vs Mpoly2 (24 h) | Mpoly1a; Mpoly1b; M poly1c; Mpoly1d vs. Mpoly2a; Mpoly2b; Mpoly2c; Mpoly2d |
| M2 | | Mpoly1 (0 h) vs Mpoly3 (48 h) | Mpoly1a; Mpoly1b; M poly1c; Mpoly1d vs. Mpoly3a; Mpoly3b; Mpoly3c; Mpoly3d |
| M3 | | Mpoly1 (0 h) vs Mpoly4 (96 h) | Mpoly1a; Mpoly1b; M poly1c; Mpoly1d vs. Mpoly4a; Mpoly4b; Mpoly4c; Mpoly4d |

The samples were collected at 24 h, 48 h, and 96 h post-inoculation as the treatment groups, while the 0 h time point served as the control group.

**Table S6.** Compared groups information of transcriptome and metabolome correlation analysis

| Define groups | Control vs. Treatment samples |
| --- | --- |
| GM1 | Tpoly1 & Mpoly1 vs. Tpoly2 & Mpoly2 |
| GM2 | Tpoly1 & Mpoly1 vs. Tpoly3 & Mpoly3 |
| GM3 | Tpoly1 & Mpoly1 vs. Tpoly4 & Mpoly4 |
